# Chemoselective bicyclobutane-based mass spectrometric detection of biological thiols uncovers human and bacterial metabolites†

**DOI:** 10.1039/d3sc00224a

**Published:** 2023-04-06

**Authors:** Amanpreet Kaur, Weifeng Lin, Vladyslav Dovhalyuk, Léna Driutti, Maria Letizia Di Martino, Miroslav Vujasinovic, J.-Matthias Löhr, Mikael E. Sellin, Daniel Globisch

**Affiliations:** a Department of Chemistry – BMC, Science for Life Laboratory, Uppsala University 75124 Uppsala Sweden Daniel.globisch@scilifelab.uu.se; b Department of Medical Biochemistry and Microbiology, Science for Life Laboratory, Uppsala University 75123 Uppsala Sweden; c Department for Digestive Diseases, Karolinska University Hospital Stockholm Sweden; d Department of Clinical Science, Intervention and Technology (CLINTEC), Karolinska Institute Stockholm Sweden

## Abstract

Sulfur is an essential element of life. Thiol-containing metabolites in all organisms are involved in the regulation of diverse biological processes. Especially, the microbiome produces bioactive metabolites or biological intermediates of this compound class. The analysis of thiol-containing metabolites is challenging due to the lack of specific tools, making these compounds difficult to investigate selectively. We have now developed a new methodology comprising bicyclobutane for chemoselective and irreversible capturing of this metabolite class. We utilized this new chemical biology tool immobilized onto magnetic beads for the investigation of human plasma, fecal samples, and bacterial cultures. Our mass spectrometric investigation detected a broad range of human, dietary and bacterial thiol-containing metabolites and we even captured the reactive sulfur species cysteine persulfide in both fecal and bacterial samples. The described comprehensive methodology represents a new mass spectrometric strategy for the discovery of bioactive thiol-containing metabolites in humans and the microbiome.

## Abstract 2

Thiol-containing metabolites in all organisms are involved in the regulation of diverse biological processes. We have developed a new method comprising bicyclobutane for chemoselective capturing and mass spectrometric analysis of this compound class.

## Graphical abstract

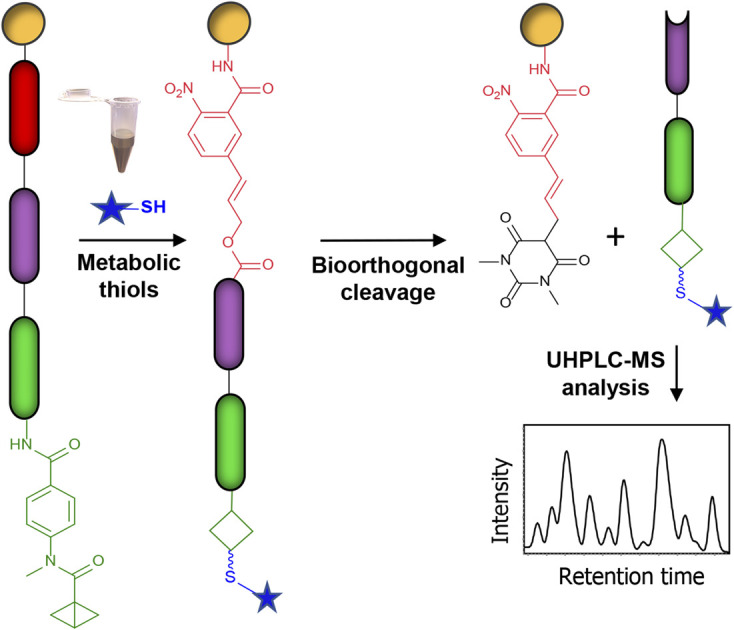

## Introduction

**Fig. 1:**
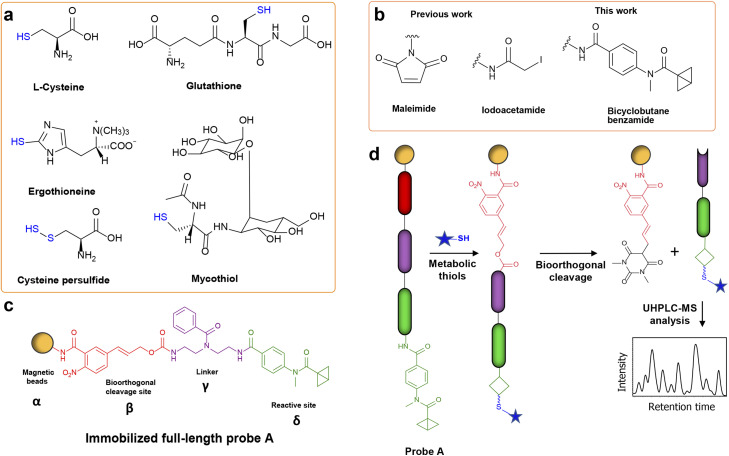
Overview of the benzamide bicyclobutane (BCB)-based thiol-metabolite analysis of biological samples. (a) Chemical structures of biologically important known thiols. (b) Overview of reactive moieties for derivatizing thiol-containing metabolites. (c) Design of immobilized probe A. The reactive site (green, δ) bearing the benzamide BCB is connected to the Noc-cleavage site (red, β) *via* a diethylenetriamine linker (purple, γ). The probe is conjugated to magnetic beads (orange, α) by an amide bond. (d) The general workflow of thiol-containing metabolites using the immobilized probe A.

Thiols are an important metabolite class for the regulation of homeostasis.^1^ Cysteine (Cys) and glutathione (GSH) are two important biological thiols that maintain cellular redox balance.^2,3^ Altered levels of these two thiols lead to higher mortality rates through pathological conditions such as coronary artery disease, Crohn's disease, and type 2 diabetes.^4,5^ Besides the vital contribution of the host metabolism, the gut microbiota also has a crucial role in modulating the levels of these metabolites.^6–8^ For example, gut microbes utilize thiol-containing amino acids in the gut, generating reactive sulfur species that are either harmful or beneficial for the host.^9^ Especially, the production of the metabolite butyrate and the survival of anaerobic gut microbiota are dependent on the glutathione concentration (Fig. 1a).^10^ In addition to the host-microbiota mutualism, these microorganisms also synthesize and maintain specific thiol-containing molecules to serve essential cellular functions.^11^ Mycothiol, an intracellular *N*-acetylcysteine-containing thiol in Actinomycetales, including *Mycobacterium tuberculosis*, chelates antimycobacterial drugs and provides drug resistance.^12^ Apart from local gut effects, microbiota-mediated thiol-modulation impacts other organs such as the brain *via* the gut–brain axis.^13,14^ While ergothioneine is mainly acquired through diet, it was also found at increased levels in the mouse feces of a depressive state model. It was upregulated by the gut commensal *Lactobacillus reuteri* to exert anti-inflammatory effects on the mouse brain.^15^ Moreover, in a recent sleep-wake study, we detected higher levels of ergothioneine in the cerebellum during the wake state of mice.^16^

Thiols also form an essential part of the human exposome. According to the Human Metabolome Database (HMDB), a vast majority of these thiols still remain to be identified in human samples.^17^ The determination of metabolism, storage, and elimination of thiols would help understand their exposure effects on human health. Although structurally diverse thiols are present ubiquitously, the discovery rate of new thiol-containing metabolites or natural products remains low. One of the reasons for these low detection rates is the presence of thiols at low concentrations in highly complex human specimens and the limitation of tools to enrich and selectively analyze this compound class with poor ionization properties.^18,19^ These unknown thiols can be products of microbial biosynthetic gene clusters (BGCs).^20,21^ In a study by Donia *et al.*, more than 3000 BGCs were identified in the microbiomes of healthy individuals.^7^ Importantly, the small molecules produced by BGCs are largely unknown and many BGCs remain silent in *in vitro* cultures.^22^ To activate these silent BGCs, strategies such as epigenetic perturbation, the upregulation of transcription factors, and the introduction of competing species have emerged.^23,24^ Although *in vitro* activation of BGCs can yield new metabolites, it fails to uncover the metabolites of microbe–microbe or microbe–host communication that exert bioactive effects on the pathological or healthy states of complex systems. Therefore, the analysis of biological samples from the physiological environment, such as feces, will provide a more comprehensive understanding of biochemical processes within the human host.^25^

Mass spectrometry (MS) is the method of choice for the investigation of known and detection of unknown metabolites.^26^ However, the MS-based analysis of complex biological samples poses challenges such as interference by the sample matrix, low mass spectrometric intensities due to poor ionization properties and low concentrations of the analytes.^27^ Metabolite enrichment *via* derivatization is one method to enhance the ionization properties.^28^ In the MS-based analyses of biological components, the most common moieties used to derivatize thiols are maleimide analogues and α-iodoacetamide (Fig. 1b).^29–31^ The main caveat of derivatizing thiols with maleimide is the instability of the maleimide–thiol conjugate under physiological pH.^32^ The reversible opening of the conjugate in the presence of free thiols is another drawback. While the moiety α-iodoacetamide has been utilized due to a high chemoselectivity in proteomics by forming an irreversible covalent bond with the thiol, it releases an iodide during the nucleophilic substitution reaction that is unfavorable for mass spectrometric analysis of small molecules.^33,34^ These simple modification reagents are thus limited due to unfavorable chemical properties, yielding poor separation of thiols from the sample matrix. Most studies have investigated plasma and urine thiols, but a comprehensive thiol analysis of fecal and microbial samples is missing.^35–37^

To overcome analytical limitations and develop a robust method for the targeted analysis of thiol-containing metabolites, we sought to utilize strained bicyclobutane (BCB) as a new chemoselective moiety for mass spectrometric metabolomics analysis (Fig. 1b). The BCB moiety was developed by Gianatassio *et al.* in 2016 and has recently only been applied as a cysteine-thiol selective electrophile for proteomics analysis.^38,39^ Towards the discovery of new thiols, we have now activated our previously reported chemical probe immobilized onto magnetic beads (α) with the BCB moiety (δ/Fig. 1c).^40^ Immobilized probe A also contains the bioorthogonal cleavage site *p*-nitrocinnamyloxy-carbonyl (Noc/β) that can be cleaved under mild conditions using Pd(0) without altering the structure of the captured metabolites.^41–44^ These two moieties are connected *via* a benzoylated diethylenetriamine linker (γ).^40^ Our advanced probe design allows for the efficient magnetic separation of captured metabolites from the sample matrix as demonstrated for amine and carbonyl analysis.^40,43,44^ The incorporated BCB moiety was utilized to capture thiols by incubating biological samples with the activated probe. After separation from the matrix, the captured metabolites were released from the magnetic beads under mild conditions (Fig. 1d). The separation of captured thiols from the sample matrix avoids sample matrix interference, leading to a more sensitive, simplified, and cleaner MS analysis of the captured metabolites.

Our chemoselective probe-based study of pathogenic bacterial cultures, human plasma and fecal samples led to the discovery of previously undetected thiols. The advantages of our probe design and the first application of the chemoselective BCB moiety in metabolomics analysis allowed for the broad-scale analysis of thiol-containing metabolites. Our new strategy overcomes the limitations of the previous reactive moieties. Unexpectedly, we also identified the highly reactive sulfur compound cysteine persulfide in bacterial and human fecal samples. Our newly developed chemical biology methodology lays the foundation for the discovery of yet unknown bioactive thiol-containing microbiome and human metabolites to gain novel insights into biological modulation by this compound class.

## Results and discussion

### Design and optimization of the BCB-based probe

**Scheme 1:**
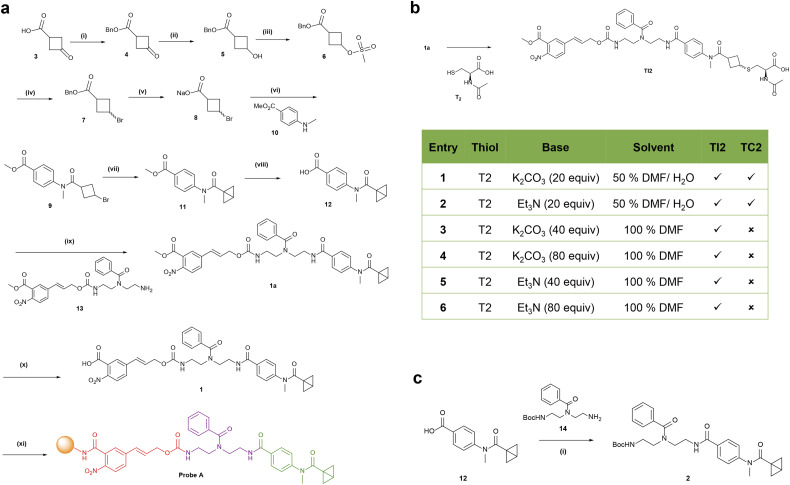
Chemical synthesis of probe A, intermediate 2 and conjugation optimization. (a) Synthesis of probe A: (i) BnOH, EDC·HCl, DMAP, and CH_2_Cl_2_; (ii) NaBH_4_ and MeOH; (iii) MsCl, Et_3_N, and CH_2_Cl_2_; (iv) LiBr and DMF; (v) NaOH and THF; (vi) T3P, DIPEA, and EtOAc/DMF; (vii) LiHMDS and toluene; (viii) Na_2_CO_3_ and MeOH/H_2_O; (ix) T3P, DIPEA, and DMF; (x) LiOH and MeOH/H_2_O (1 : 1); (xi) amine-derivatized beads, PyBOP, HOBt, DIPEA, and DMF. (b) Optimization of thiol conjugation with the probe under different conditions. Compound 1a was treated with *N*-acetyl-l-cysteine (T2) as a test substrate under six conditions to obtain the conjugated product TI2 with or without the carbamate hydrolysis by-product TC2. (c) Synthesis of simplified probe 2. Reaction conditions: (i) T3P, DIPEA, and DMF.

The design of the full-length probe 1 is based on our recently developed method for capturing carbonyl metabolites.^40^ Probe 1 was synthesized using an optimized synthetic route, followed by immobilization onto magnetic beads to yield the final probe A that is activated for direct sample treatment (Scheme 1a). Carboxylate 8 was prepared from commercially available carboxylic acid 3 in 5 steps (Scheme 1a).^38^ The amide coupling of the diastereomeric mixture of carboxylate 8 with methylbenzoate 10 using propanephosphonic acid anhydride (T3P) formed isomerically pure 9. Bromocyclobutane 9 was activated with LiHMDS to yield bicyclobutane 11. Basic hydrolysis of methylbenzoate 11 yielded the carboxylate 12, which was coupled with 13 to form 1a. The saponification of 1a resulted in compound 1 that was coupled with magnetic beads to obtain the final product A.

BCB was described to react with cysteine thiols using K_2_CO_3_ in an aqueous DMF solution.^38,39^ As our probe contains the base-labile carbamate moiety that hydrolyzed under these conditions (Scheme 1b), we optimized the conjugation conditions of 1a with substrate *N*-acetyl-l-cysteine (T2, Scheme 1b). The reaction mixtures were analyzed using ultrahigh-performance liquid chromatography-mass spectrometry (UHPLC-MS). Our condition screening identified triethylamine as a base in dry DMF to form the desired conjugate TI2 without the hydrolysis by-product TC2. These conditions were utilized for the conjugation chemistry of thiols in biological samples.

**Fig. 2:**
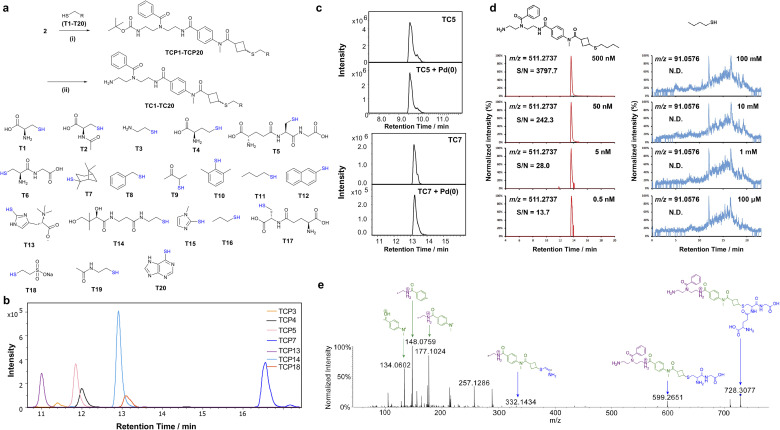
Capture efficiency and analysis of the conjugated thiols. (a) Reactivity of simplified probe 2 with thiols T1–T20. Reaction conditions: (i) 2, thiol (T1–T20), Et_3_N, DMF or K_2_CO_3_, and DMF/H_2_O (1 : 1); (ii) TFA. (b) Representative mass spectrometric extracted ion chromatograms of 7 selected thiol conjugates that were successfully captured (TCP3, TCP4, TCP5, TCP7, TCP13, TCP14, and TCP18) after treatment with 2. Conditions: Et_3_N in DMF at 28 °C for 16 h. (c) Stability of two Boc-deprotected thiol conjugates [TC5 (glutathione) and TC7 (2,3,10-mercaptopinane)] after Pd(0) treatment at 25 °C for 18 h. Top: extracted ion chromatograms (EICs) of the corresponding thiol conjugate before Pd(0) treatment. Bottom: unchanged EICs after Pd(0) treatment. (d) EICs of the LOD measurements comparing 1-butanethiol with conjugated 1-butanethiol. (e) MS/MS fragmentation of the thiol glutathione conjugate TC5 with CID (30–75 eV).

To evaluate the efficiency of thiol conjugation with BCB, we synthesized probe 2 through amide coupling of BCB analogue 12 and the Boc-protected diethylenetriamino-linker 14 (Scheme 1c). Compound 2 was separately incubated with commercially available thiols of diverse chemical structures under basic conditions for 16 h and analyzed *via* UHPLC-MS (Fig. 2a). All 20 thiols T1–T20 reacted efficiently with probe 2 to form the desired thiol conjugates T1–T20 as illustrated by the extracted ion chromatograms (Fig. 2B and S1†). These thiol substrates representing the diversity of metabolites in biological samples include l-cysteine T1, dietary compound 1-propanethiol T16, metabolic intermediates, cysteamine T3, Cys–Gly T6 and (R)–pantetheine T14, flavoring agents, 2,3,10-mercaptopinane T7 and 2-naphthalene thiol T12 as well as the microbiome-derived amino acid l-ergothioneine T13 (Fig. 2a/b and S2†). T13 and 6-mercaptopurine T20 predominantly exist as the thione tautomer at physiological pH and are also conjugated under our experimental conditions.^45^ For lipoic acid, we reduced the disulfide bond with NaBH_4_ prior to the treatment with simplified probe 2 (Scheme S1, Fig. S3†). Using an NMR based assay, BCB-intermediate 11 reacted with *N*-acetylcysteamine within 8 h to completion (Fig. S4/S5†). To assess the chemoselectivity of the thiol conjugation, we treated probe 2 with thiol T2 in the presence of two other nucleophiles, serine and lysine. The LC-MS analysis only detected the thiol conjugate TCP2 with no corresponding conjugates of lysine and serine, confirming the chemoselectivity of the BCB moiety (Fig. S6†). We also tested the selectivity of the common thiol-conjugation moiety iodoacetamide and identified reaction with all three substrates (Fig. S7†). As we employed Pd(0) catalysis in the final step of the analysis, we tested the stability of the thiol-conjugates T1-T3, T7, T10, T13, T15 and T19 in the presence of all cleavage reagents (Fig. 2c). No change in the LC-MS spectra of the treated and the untreated conjugates was observed, confirming the stability of the conjugates throughout our entire procedure. The only exception was the cysteine conjugate TC1 (Fig. S8†). This retention time shift is presumably due to the reported coordination of palladium(ii) with sulfur-containing amino acids.^46^ To evaluate the MS-sensitivity of the conjugated metabolites, we synthesized 1-butanethiol conjugate TC11 and performed limit of detection (LOD) measurements. The LOD analysis revealed a high mass spectrometric sensitivity of 0.5 nM for the conjugated metabolite (Fig. 2d). No MS signal was detectable even at 100 mM for the unconjugated 1-butanethiol. The high sensitivity of the conjugate in these LOD experiments is in a similar range to the captured and released conjugates in the biological sample due to the reconstitution after removal of the sample matrix into a similar solvent system.

Upon determining the properties of the conjugates, we performed MS/MS fragmentation experiments for nine thiol conjugates TC2–TC5, TC13, TC14, TC16, TC17, and TC20. For example, the glutathione conjugate TC5 demonstrates the high resolution of our fragmentation-based structure elucidation (Fig. 2e). This spectrum depicts four tag-specific MS fragments (134.0602, 148.0759, 177.1024, and 257.1286) that can be used as diagnostic markers for captured thiols. We observed at least two fragments in all tested MS/MS fragmentation spectra. These fragmentation spectra were utilized for comparison with the natural metabolite in the biological sample.

Before applying our BCB-probe to biological samples, we tested the treatment sequence of the immobilized probe A with two individual thiols. We conjugated the full-length probe 1 to amine-derivatized Magnabind™ beads through amide coupling to obtain the immobilized probe A. The beads were then individually incubated with pantetheine and cysteamine using triethylamine in DMF at 30 °C for 18 h. These beads were washed and treated using our Pd(0) cleavage conditions in THF.^40^ The beads were then magnetically separated from the reaction mixture and the subsequent LC-MS analysis demonstrated the successful conjugation of the two test metabolites (Fig. S9†).

### Analysis of thiol-containing metabolites in human plasma and fecal samples

**Fig. 3:**
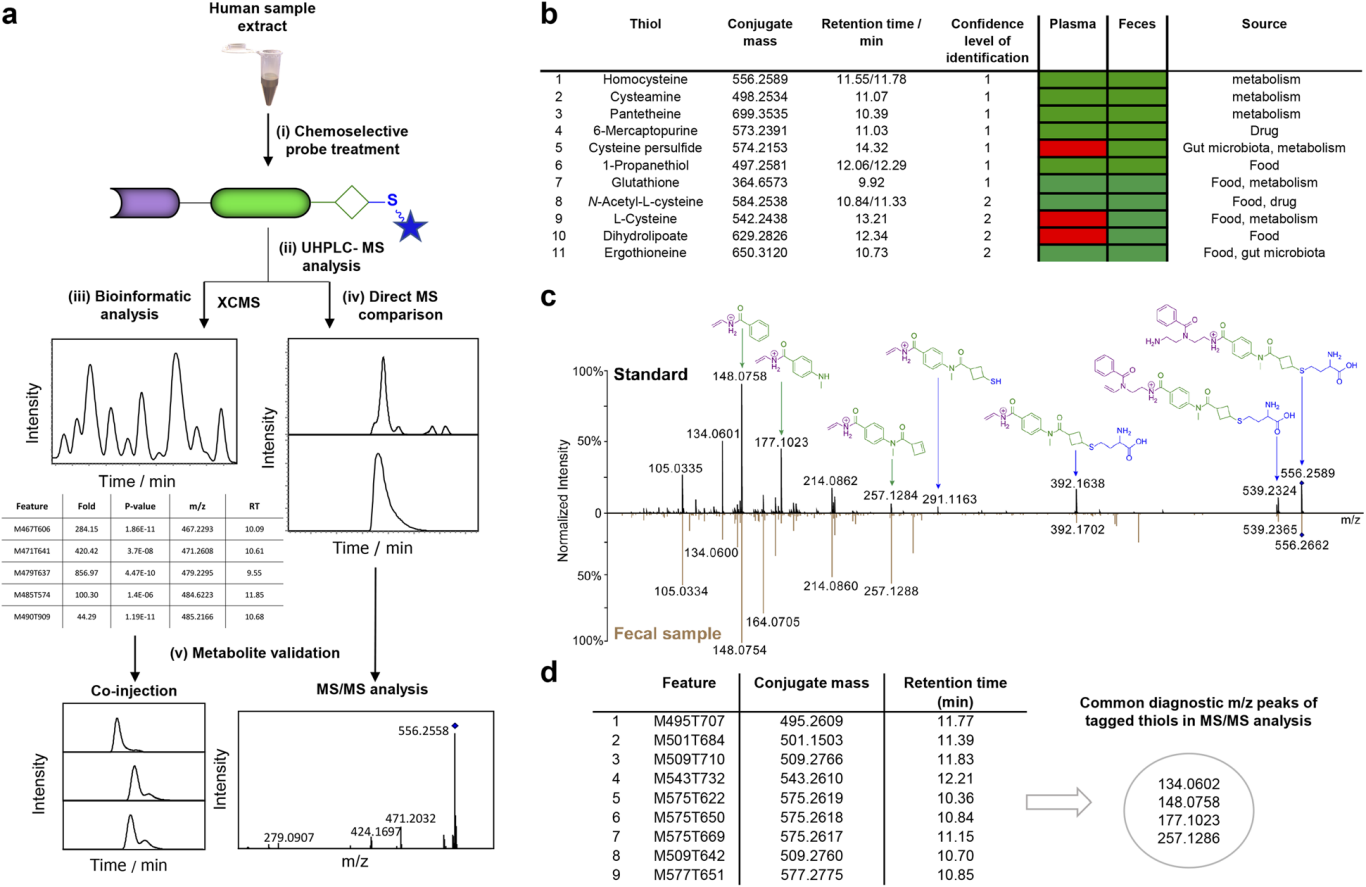
Thiol-based analysis of human samples. (a) Overview of the UHPLC-MS analysis of biological samples. (i) Treatment of samples with chemoselective probe A; (ii) UHPLC-analysis of captured thio-metabolites; (iii) data analysis *via* bioinformatic analysis using XCMS or (iv) *via* direct MS comparison; (v) metabolite structure validation by co-injection and MS/MS experiments. (b) Overview of identified metabolites in human plasma and fecal samples. (c) MS/MS fragmentation pattern comparison between the synthetic standard of homocysteine and the detected metabolite in the fecal sample. (d) Representative *m*/*z* values for nine metabolites that have at least two of the common MS/MS fragments derived from the probe tag.

Following the successful conjugation and stability assessment of individual thiols, we treated one human plasma sample with immobilized BCB-probe A as this is the most commonly studied sample type for human thiols (Fig. 3a).^47^ Prior studies have mainly focused on investigating redox changes related to the common thiol-containing cysteine, glutathione or homocysteine.^35,48–50^ Overall, only a few studies have aimed for a broad-scale analysis of biological thiols and were hampered in metabolite coverage due to analytical limitations.

The sample extract was treated with probe A using triethylamine in DMF at 30 °C for 22 h (Fig. 3a). A parallel incubation of beads without immobilized probe A with the plasma extract under the same conditions served as the control experiment. All magnetic beads were processed using our optimized conditions. Upon cleavage of the captured and tagged metabolites from the beads, the solvent was removed and the mixture was reconstituted for analysis *via* UHPLC-MS. The MS-data pre-processing using the XCMS platform in *R* identified 32 376 features that differed between the probe-treated plasma sample and the control sample.^51^ Filtering the raw data with the following criteria reduced the features to 3517: (i) *p*-value < 0.05; (ii) fold-change ≥ 5; (iii) *m/z* > 421.2235 (*i.e.* higher than the *m/z* value of the unconjugated probe). Further removal of isotopologues and unspecific features compared to the control sample yielded 81 features that represent thiol-containing metabolites.

In the next step, we focused on validating the chemical structure for the detected mass spectrometric features. The six thiols cysteamine, the anticancer and immunosuppressant drug 6-mercaptopurine, 1-propanethiol, homocysteine, pantetheine, and glutathione were validated at the highest confidence either through comparison of MS/MS fragmentation spectra, retention times or co-injection experiments with authentic synthetic reference standards (confidence level 1, Fig. 3b). MS/MS fragmentation comparison is demonstrated for the validation of homocysteine-conjugate TC4 (Fig. 3c). Additionally, the MS/MS fragmentation spectra for nine metabolites contained at least two of the specific fragments for the probe tag, which further confirms the presence of a thiol (Fig. 3d, Table S1†). Among the validated thiol structures, 1-propanethiol and pantetheine (also known as the *Lactobacillus bulgaricus* factor) were detected for the first time in human plasma. Pantetheine is a precursor for CoA synthesis and an essential growth factor not only for humans but also for most lactic acid-producing bacteria and bifidobacteria. Recently, the deficiency of pantetheine was linked to patients with cystic fibrosis.^52^ Moreover, detecting the food-derived metabolite 1-propanethiol is a first step towards the development of dietary biomarkers.^53,54^

Encouraged by the high coverage of different thiol-containing metabolites and discovery of unexpected metabolites in human plasma, we next focused our investigation on human fecal samples. This sample type contains microbiome-derived metabolites, dietary thiols, and pharmaceuticals. Although microbiota-mediated sulfur metabolism is important for human physiology and fecal samples contain microbiota-derived sulfur metabolites, to the best of our knowledge this sample type has remained unexplored for comprehensive analyses of thiol-containing metabolites.^25^ To maximize the detection of thiol-containing metabolites, we pooled fecal samples collected from eight different individuals and extracted the metabolites. This metabolite extract was treated with probe A, followed by UHPLC-MS analysis as described for the plasma sample analysis (Fig. 3a). Employing the same MS data analysis, we identified 61 features in this pooled fecal sample. By comparing the co-injection experiments and MS/MS fragmentation data of conjugated fecal thiols with synthetic standards, we validated the chemical structure for 11 thiols (Table S2†). These thiols included the previously reported cysteine, homocysteine, *N*-acetyl-l-cysteine, pantetheine, and 1-propanethiol. Importantly, we detected cysteamine, dihydrolipoate, and 6-mercaptopurine for the first time experimentally in human feces. These metabolites have only been predicted by HMDB as potential fecal metabolites. Besides thiols, our method is suitable for capturing thiones such as ergothioneine and 6-mercaptopurine. As our BCB-based method uses non-aqueous conditions to conjugate thiols, the thiol tautomer of thiones is easier to capture than for other reported analytical methods.^45^

**Fig. 4:**
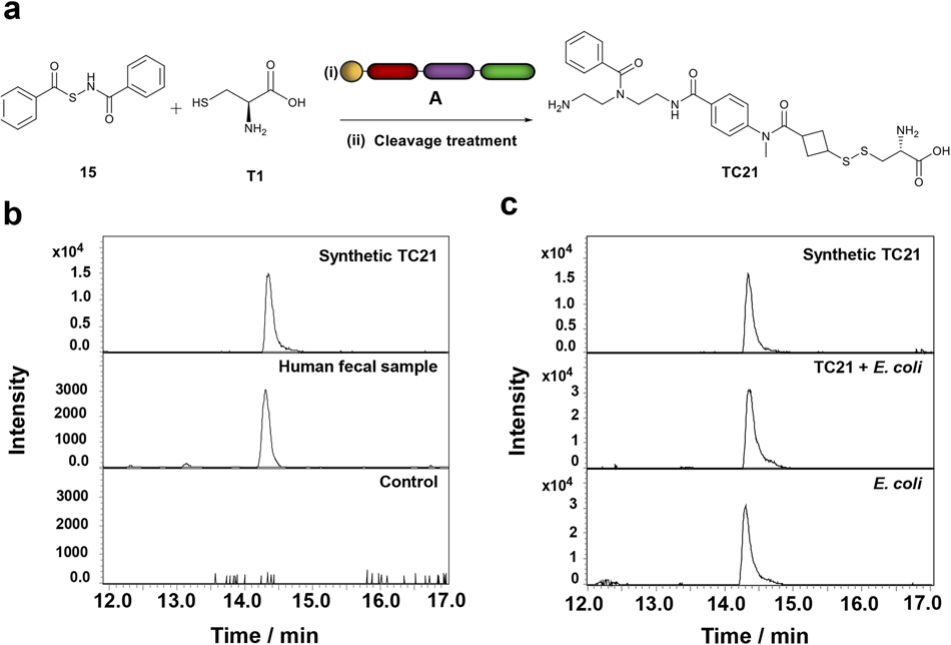
Validation of cysteine persulfide in biological samples. (a) Synthesis of cysteine persulfide–probe conjugate TC21. Reaction conditions. (i) 15, T1, probe A, THF, and PBS; (ii) 1,3-dimethylbarbituric acid, triphenylphosphine, Pd(OAc)_2_, and THF. (b) Comparison of extracted ion chromatograms (EICs) of synthetic TC21 and natural cysteine persulfide in the human fecal sample and the control sample. (c) Co-injection experiment: EIC comparison of the synthetic TC21, the co-injected synthetic TC21 and the *E. coli* lysate as well as the metabolite in *E. coli*.

Interestingly, among these features that correspond to thiol-containing metabolites, we detected cysteine persulfide (CysSSH), which is of biological importance but has not yet been detected in human feces. To validate CysSSH, we synthesized a reference conjugate by reacting our immobilized probe with a hydrogen sulfide donor developed by Zhao *et al.* as no synthesis for isolation of this metabolite has been reported.^55^ This study proposed the production of cysteine persulfide as an intermediate in the synthesis of H_2_S. Therefore, we treated the H_2_S donor 15 with cysteine, followed by our probe A to capture the intermediate cysteine persulfide (Fig. 4a and S10†). The LC-MS analysis of this mixture revealed a clean mass spectrometric signal of the conjugate TC21 that perfectly matched the natural cysteine persulfide conjugate from the fecal sample (Fig. 4a/b). In contrast to the conjugation conditions for thiols, we captured cysteine persulfide under non-basic conditions, likely due to the higher nucleophilicity of persulfides than the corresponding thiols.^56^ This result demonstrates that we can even capture reactive and short-lived sulfur-containing intermediates.

We predicted the presence of CysSSH in fecal samples based on a *in vivo* study by Uchiyama *et al.* in a mouse model, which demonstrated that CysSSH strengthens the host antioxidant capacity by reducing the generation of reactive oxygen species.^9^ They also demonstrated that the Ruminococcaceae and Lachnospiraceae species have a high capacity to produce CysSSH due to a PLP-dependent enzyme that converts cystine to CysSSH. H_2_S was earlier considered the sole reactive sulfur species eliciting the biological effects. Several studies have now linked the formation of persulfides through the reaction of H_2_S with free thiols or disulfide-containing proteins to the protection of cells.^57–61^ Furthermore, the role of persulfides to protect bacteria against external stress has been elucidated, such as antibiotics or host-generated reactive oxygen species.^62,63^ Recently, Ono *et al.* elucidated the inactivation of β-lactam antibiotics by CysSSH in *Escherichia coli*.^64^

### Analysis of thiols in bacterial samples

Thiol-containing metabolites have been linked to virulence modulation in bacterial pathogens.^65^ Furthermore, important bioactive compounds from the microbiome, including sulfur-containing colibactin and biosynthetic intermediates, have been proven challenging to isolate and analyze.^66,67^. To explore the capacity of our chemoselective probe in this context, we utilized the BCB-based methodology for the investigation of thiol-containing metabolites in three related enterobacterial species: *Escherichia coli* (*E. coli*), *Salmonella enterica* Typhimurium (*Salmonella*) and *Shigella flexneri* (*Shigella*). These enterobacteria exemplify prominent Gram-negative gut bacteria that vary in their virulence potential.

As a validation experiment, we first assayed *E. coli* cell lysates in regular culture or exposure to oxidative stress, which has been investigated previously in proteomics research.^68^ We observed several qualitative differences between the cultures and also identified decreased levels of glutathione (GSH) as a result of oxidative stress (Fig. S11 and Tables S3–S5†). The reason that GSH is consumed and dimerized in the process of neutralizing reactive oxygen species (ROS) has been described to protect the organism from oxidative damage.^69^

**Fig. 5:**
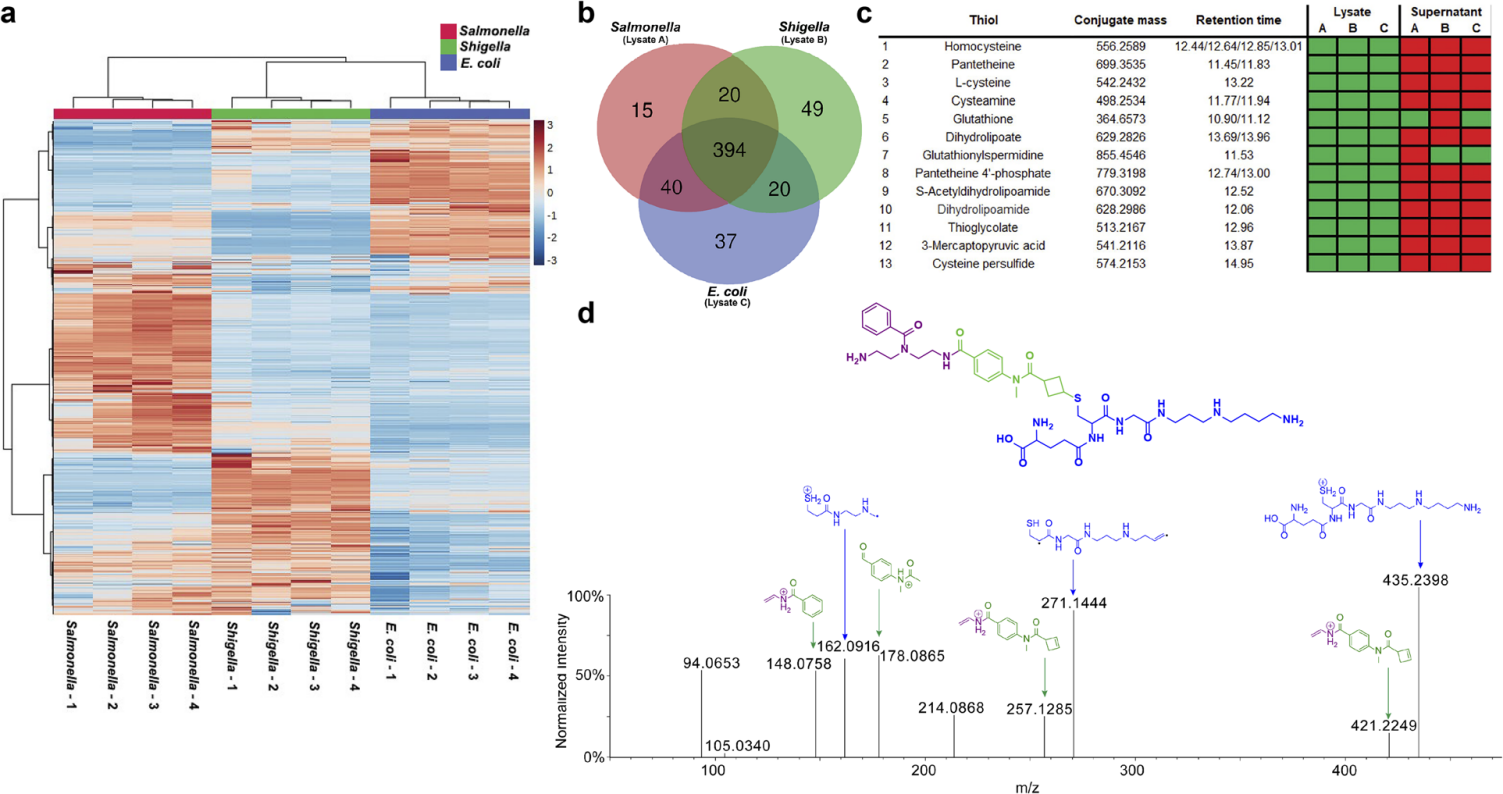
Bacterial thiol-containing metabolites. (a) Heatmap illustrating different levels of thiols in the bacterial lysates. (b) Venn diagram depicting the distribution of common and different features among the three bacterial lysates. (c) Overview of annotated thiols in the lysates of *Salmonella* (Lysate A), *Shigella* (Lysate B) and *E. coli* (Lysate C). (d) MS/MS fragmentation spectrum of the detected conjugate of glutathionylspermidine with annotations of fragments.

Next, the cell lysates of stationary phase *E. coli*, *Salmonella* and *Shigella* cultures were incubated with probe A under the same conditions as described for the human samples. The comparison of all samples provided a global overview of the distribution of intracellular thiol-containing metabolites for each bacterium (Fig. 5a, Table S6†). This heatmap demonstrates quantitative differences of thiols between the three genetically similar bacteria with substantial differences in *Salmonella* compared to *E. coli* and *Shigella*. We also identified the core bacterial thiol metabolome of 394 features and specific bacterial metabolites for each bacterium (Fig. 5b). The targeted analysis based on entries in the *E. coli* metabolite database (ECMDB) validated 13 thiol-containing metabolites in *E. coli*.^70^ For example, we captured the common thiols homocysteine, pantetheine, cysteamine, and glutathione in all three enterobacteria (Fig. 5c).

Interestingly, we detected CysSSH in all three single cultures as well (Fig. 4c/5c). Of further importance, our analysis detected glutathionylspermidine (Gsp) in all three cultures. Gsp was first isolated from *E. coli* where it accumulates during the stationary phase and under anaerobic conditions.^71^ For the other two pathogenic bacteria, *Shigella* and *Salmonella*, only the bifunctional enzyme glutathionylspermidine synthetase/amidase has been identified without any experimental isolation of Gsp.^72,73^ The chemical structure of glutathionylspermidine was validated using MS/MS fragmentation analysis at confidence level 2 due to the lack of an internal standard. The mass spectrometric fragments of the tag as well as the specific fragments for the conjugated Gsp were detected (Fig. 5d).

We also investigated thiol-containing metabolites in bacterial supernatants. As expected, our analysis yielded fewer compounds than the intracellular analysis, possibly due to dilution and potential intracellular utilization without excretion. Glutathione was captured in the supernatants of *E. coli* and *Salmonella* that confirms the reports of an active glutathione export system in *E. coli* and *Salmonella* under aerobic growth conditions.^74,75^ Furthermore, we detected glutathionylspermidine in the supernatants of *E. coli* and *Shigella* cultures.

## Conclusions

The gut microbiome produces a plethora of bioactive metabolites that can either interact beneficially or adversely with the human host. These metabolites can potentially be future drug lead structures, serve as disease or dietary biomarkers or lead to the discovery of unknown drug targets. We have here developed a new Chemical Biology method that overcomes analytical limitations and complex experimental microbiology setups. Herein, we report the first broad-scale analysis of thiol-containing metabolites in human and microbiome samples. Charging our probe with the recently introduced reactive site bicyclobutane (BCB) allowed for the first targeted MS-based metabolomics and chemoselective investigation of thiol-containing metabolites. These results demonstrate that our approach is superior to any other method reported. We further exemplified the robust and versatile application of this chemoselective probe immobilized to magnetic beads for mass spectrometric investigation of metabolites across three different biological sample types. A series of previously undetected thiol-containing metabolites of dietary, bacterial, and human origin, and the reactive sulfur species cysteine persulfide were identified. The major advantages of our method are simplified LC-MS spectra, irreversible reaction with thiol-containing metabolites, mild cleavage conditions as well as structure elucidation *via* MS/MS fragmentation. This new Chemical Biology tool paves the way for comprehensive mass spectrometric investigations of bioactive thiol-containing metabolites.

## Data availability

The datasets supporting this article have been uploaded as part of the ESI.†

## Author contributions

D. G. conceived, designed and supervised the study. A. K. and W. L. designed experiments. A. K. and L. D. performed chemical synthesis. A. K. and W. L. performed bioinformatic analysis. A. K. analyzed data. A. K. and V. D. performed mass spectrometric experiments. D. G. and M. E. S. acquired funding. M. V. and J.-M. L. were responsible for ethical approval, and human patient sample collection and selection. M. L. D. M. and M. E. S. prepared bacterial culture samples. D. G. and A. K. wrote the manuscript with contributions from all other authors.

## Conflicts of interest

There are no conflicts to declare.

## Supplementary Material

SC-014-D3SC00224A-s001
