# Supplementary material for: Chemoselective bicyclobutane-based mass spectrometric detection of biological thiols uncovers human and bacterial metabolites
Source: Chem Sci. 2023 Apr 6;14(20):5291–301. doi: 10.1039/d3sc00224a (PMC10207876; doi:10.1039/d3sc00224a)

Electronic Supplementary Material (ESI) for Chemical Science.
This journal is © The Royal Society of Chemistry 2023

SUPPLEMENTARY INFORMATION

Chemoselective bicyclobutane-based mass spectrometric detection of biological thiols uncovers human and bacterial metabolites

Amanpreet Kaur,[a] Weifeng Lin,[a] Vladyslav Dovhalyuk,[a] Léna Driutti,[a] Maria Letizia Di Martino,[b] Miroslav Vujasinovic,[c] J.-Matthias Löhr,[c,d] Mikael E. Sellin,[b] and Daniel Globisch*[a]

[a] Department of Chemistry - BMC, Science for Life Laboratory, Uppsala University, 75124 Uppsala, Sweden

[b] Department of Medical Biochemistry and Microbiology, Science for Life Laboratory, Uppsala University, 75123 Uppsala, Sweden

[c] Department for Digestive Diseases, Karolinska University Hospital, Stockholm, Sweden

[d] Department of Clinical Science, Intervention and Technology (CLINTEC), Karolinska Institute, Stockholm, Sweden

Table of Contents

1. Supporting Figures and Supporting Scheme.......... 4

Figure S1. Extracted ion chromatograms of the Boc-protected thiol conjugates **TCP1- TCP2**, **TCP6**, **TCP8- TCP12**, **TCP15- TCP17**, **TCP17**, **TCP19- TCP20**.......... 4

Figure S2. Extracted ion chromatograms of the thiol conjugates **TC1-TC20**.......... 6

Figure S3. Extracted ion chromatograms of the lipoic acid conjugates **TCLA1** and **TCLA2**.......... 7

Figure S4. Time-based NMR monitoring of the conversion rate of intermediate **11** with *N*-acetylcysteamine. 7

Figure S5. Conversion rate of thiol capture based on integration of NMR experiment in Figure S4.8

Figure S6. Extracted ion chromatograms of the conjugates detected in the chemoselectivity test demonstrating selectivity for thiols.......... 8

Figure S7. Extracted ion chromatograms of the conjugates detected in the chemoselectivity test using iodoacetamide.......... 9

Figure S8. Extracted ion chromatograms of thiol conjugates before and after the stability test. 10

Figure S9. Extracted ion chromatograms of the captured cysteamine conjugate (**TC3**) and pantetheine (**TC14**),.......... 11

Figure S10. Extracted ion chromatograms of the synthetic cysteine persulfide conjugate **TC21** captured using probe **A**. 11

Figure S11. **A**) Heatmap of oxidative stress comparison experiment in *E. coli*; **B)** Volcano plot of oxidative stress comparison. 12

Scheme S1. Synthesis of standard lipoic acid probe conjugates **TCLA1** and **TCLA2** for the LC-MS based analysis.......... 13

2. Supporting Tables.......... 14

Table S1. Features of thiol-containing metabolites in the human plasma.......... 14

Table S2. Features of thiol-containing metabolites in the pooled human feces.......... 16

Table S3. Features only detected in the control samples compared to the oxidative stress sample. 17

Table S4. Features only detected in the oxidative stress sample.......... 17

Table S5. Semi-quantitative analysis of GSH in the comparison between control samples and oxidative stress samples through comparison of an internal standard.......... 18

Table S6. Detected Top100 features from bacteria lysates with intensities.......... 19

Table S7. Metadata of patients for the pooled fecal and plasma sample. 22

3. General.......... 23

4. Synthetic procedures.......... 24

*Preparation of compound **5*** 24

*Preparation of compound **6*** 24

*Preparation of compound **7*** 25

*Preparation of compound **8*** 26

Preparation of compound **10** 26
*Preparation of compound **9*** 27
*Preparation of compound **11*** 28
*Preparation of compound **12*** 29
*Preparation of compound **2*** 30
*Preparation of compound **1a*** 31
*Preparation of compound **1*** 32
Synthesis of reduced lipoic acid 32
*Preparation of 1-butanethiol-probe conjugate **TC11** for the LOD determination* 33
*Preparation of Boc-protected cysteine persulfide conjugate **TCP21** for the LC-MS analysis* 34
5. Methods 35
5.1 Preparation of immobilized probe A 35
5.2 Preparation of LC-MS standard of TC21 35
5.3 Preparation of fecal metabolite extracts 36
5.4 Preparation of plasma metabolite extracts 36
5.5 Treatment of fecal metabolite extract 36
5.6 Treatment of plasma metabolite extract 36
5.7 Preparation of bacterial strain lysates and supernatants 37
5.8 Cleavage of the bead-bound probe A 37
5.9 Preparation of probe-conjugated thio-intermediates TCP1-TCP20 38
5.10 Preparation of probe-conjugated standards TC1-TC20 38
5.11 Stability of thiol conjugates under Pd(0) conditions 38
5.12 Chemoselectivity test 38
5.13 LOD experiment 38
6. Ethical approval 39
7. References 40
8. NMR spectra 40

1. Supporting Figures and Supporting Scheme

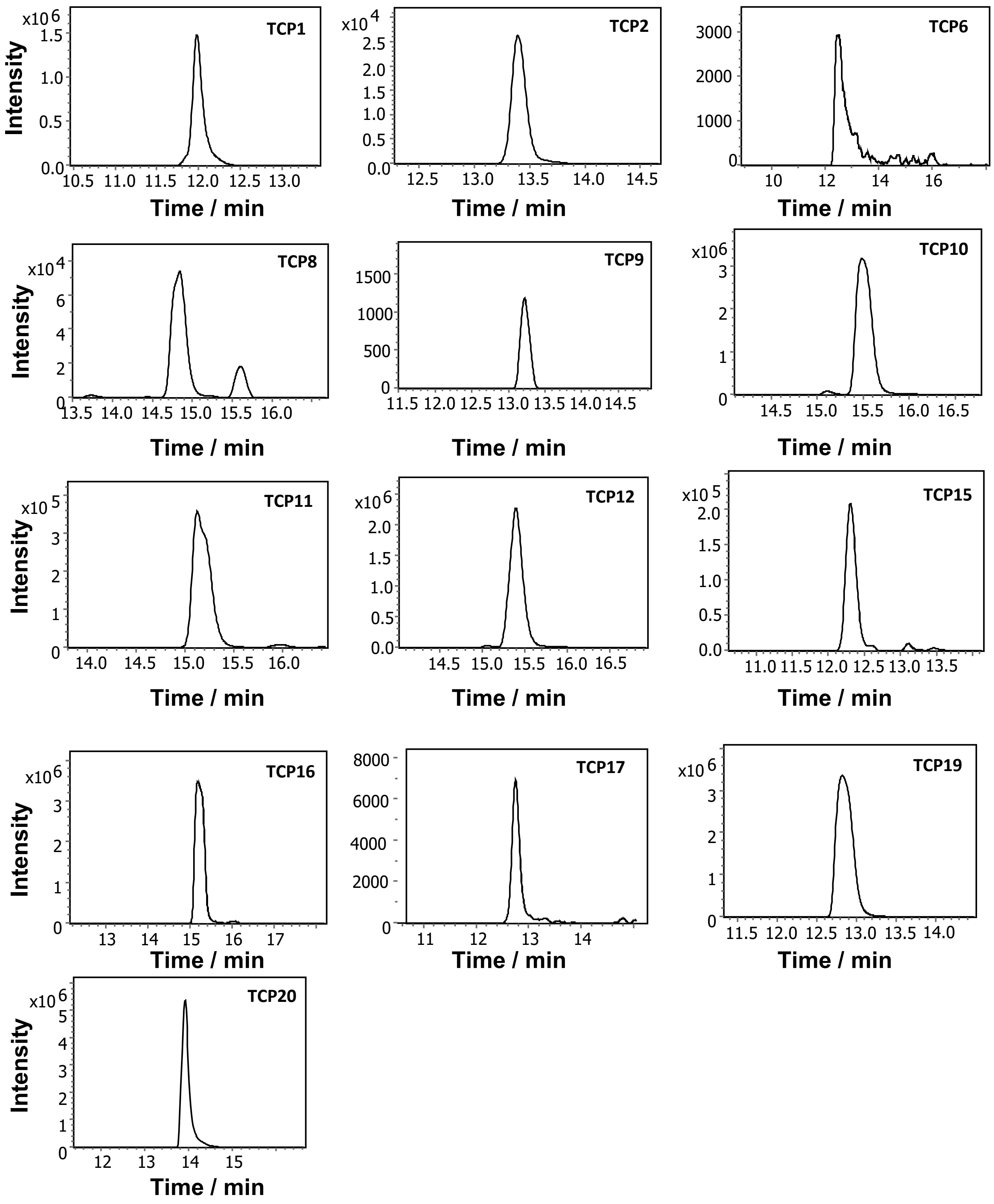

Figure S1. Extracted ion chromatograms of the Boc-protected thiol conjugates **TCP1- TCP2**, **TCP6**, **TCP8- TCP12**, **TCP15- TCP17**, **TCP17**, **TCP19- TCP20**.

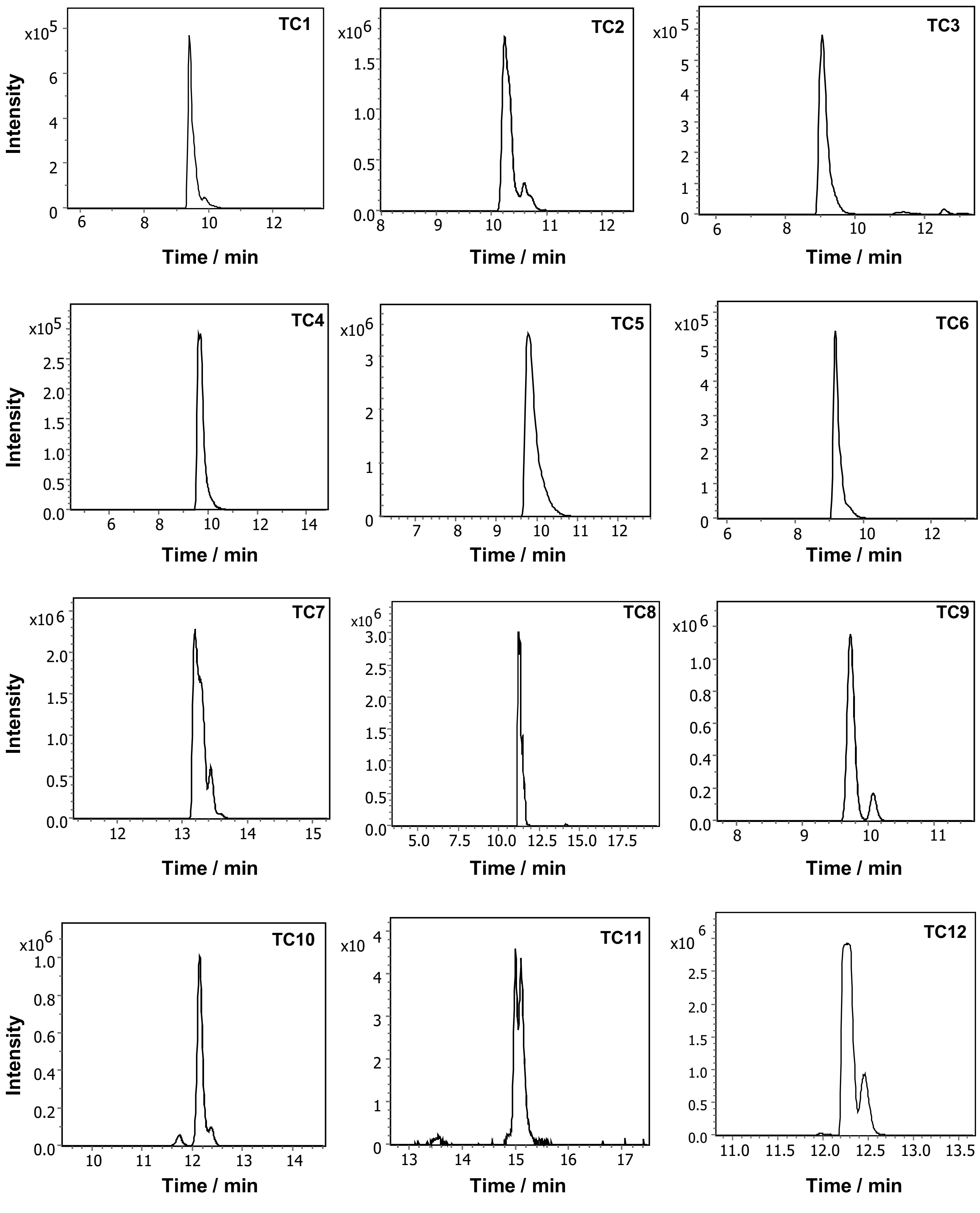
TC1
x10^5
Intensity
Time / min
TC2
x10^6
Time / min
TC3
x10^5
Time / min
TC4
x10^5
Intensity
Time / min
TC5
x10^6
Time / min
TC6
x10^5
Time / min
TC7
x10^6
Intensity
Time / min
TC8
x10^6
Time / min
TC9
x10^6
Time / min
TC10
x10^6
Intensity
Time / min
TC11
x10^4
Time / min
TC12
x10^6
Time / min

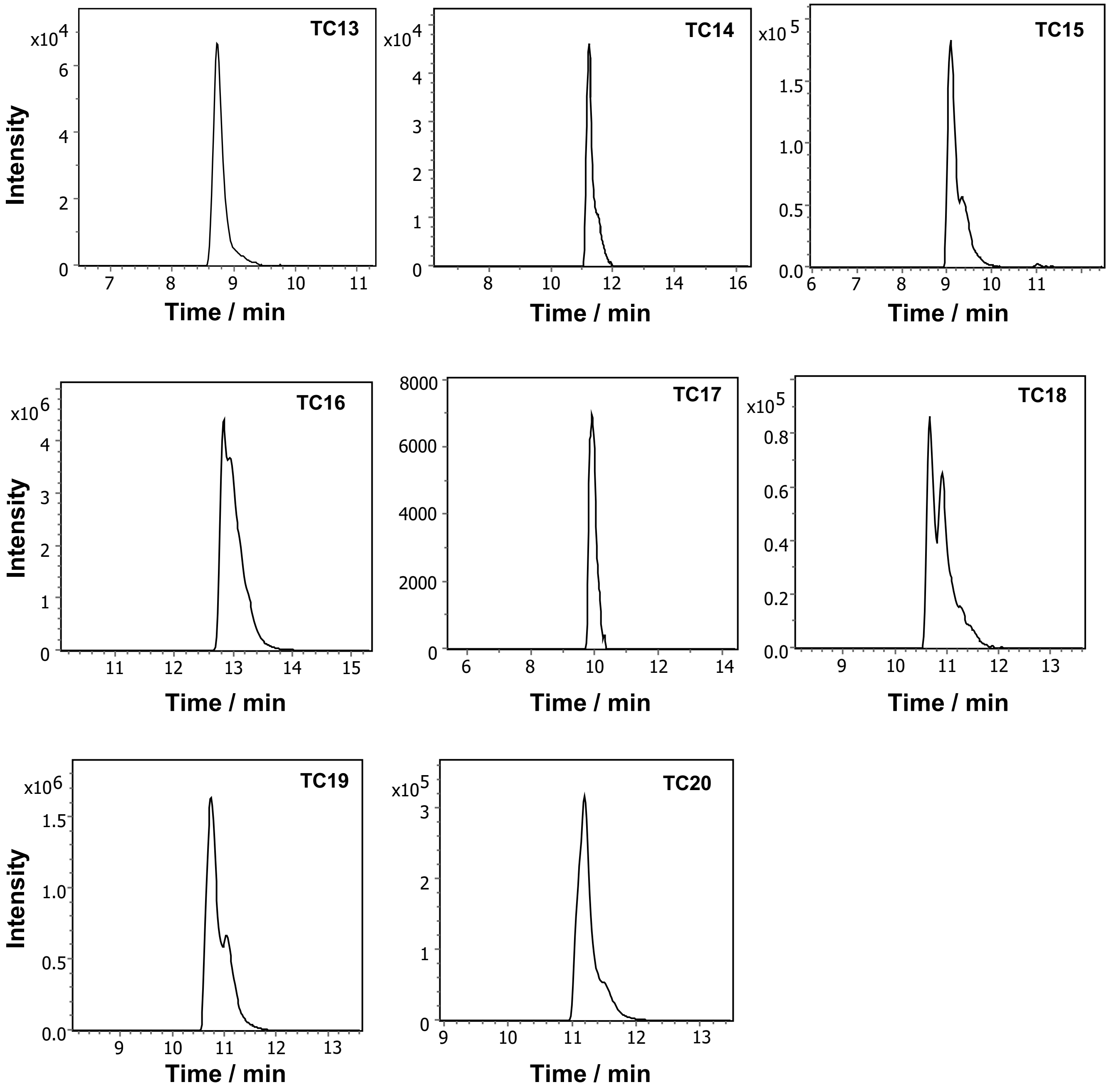

Figure S2. Extracted ion chromatograms of the thiol conjugates **TC1-TC20**.

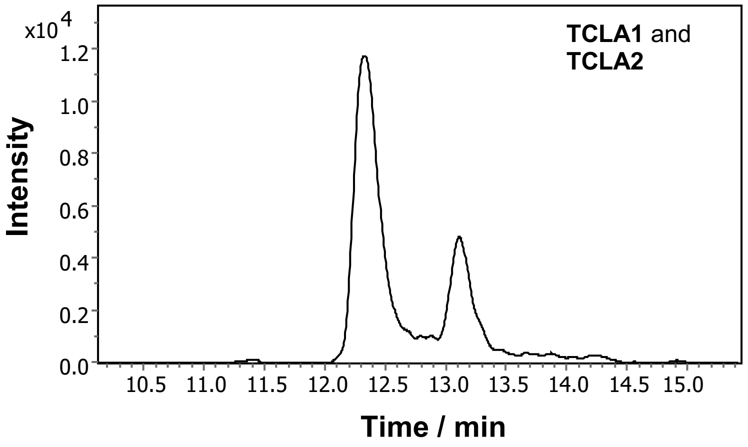

Figure S3. Extracted ion chromatograms of the lipoic acid conjugates **TCLA1** and **TCLA2**.

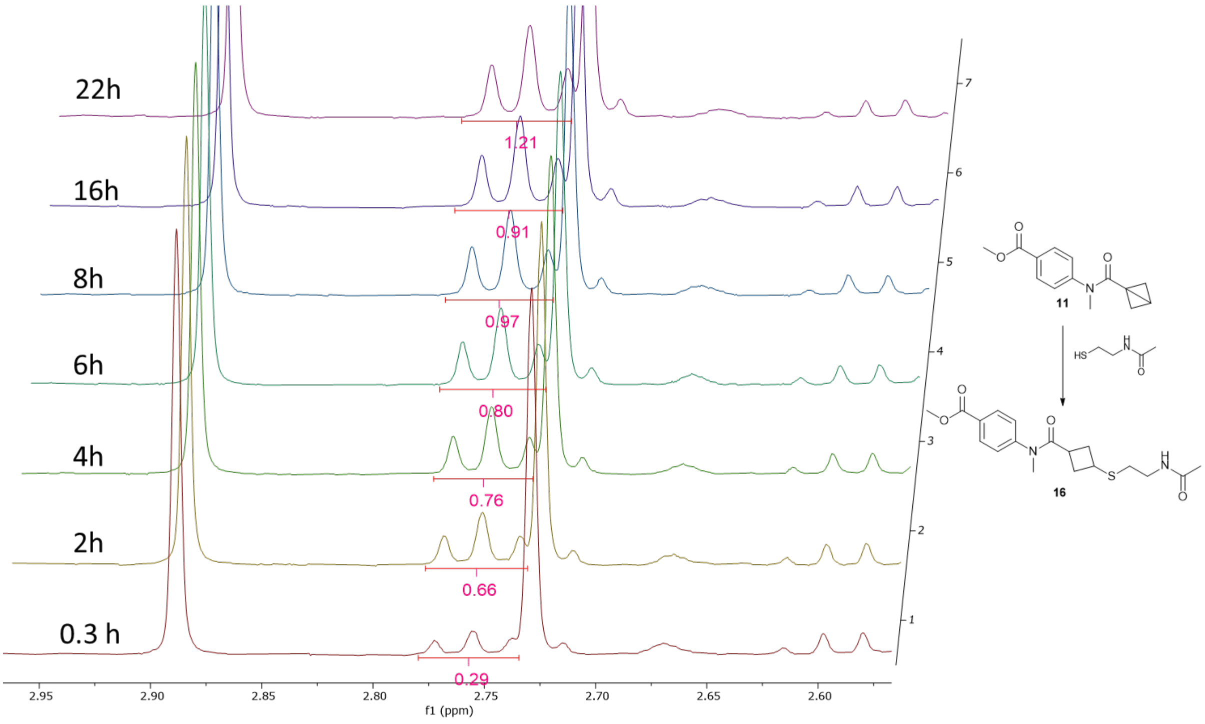

Figure S4. Time-based NMR monitoring of the conversion rate of intermediate **11** with *N*-acetylcysteamine.

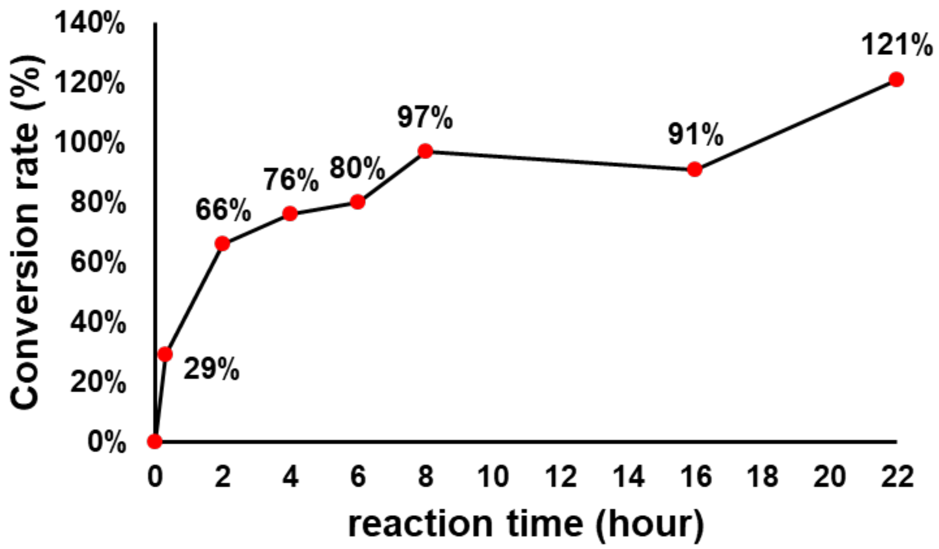

Figure S5. Conversion rate of thiol capture based on integration of NMR experiment in Figure S4.

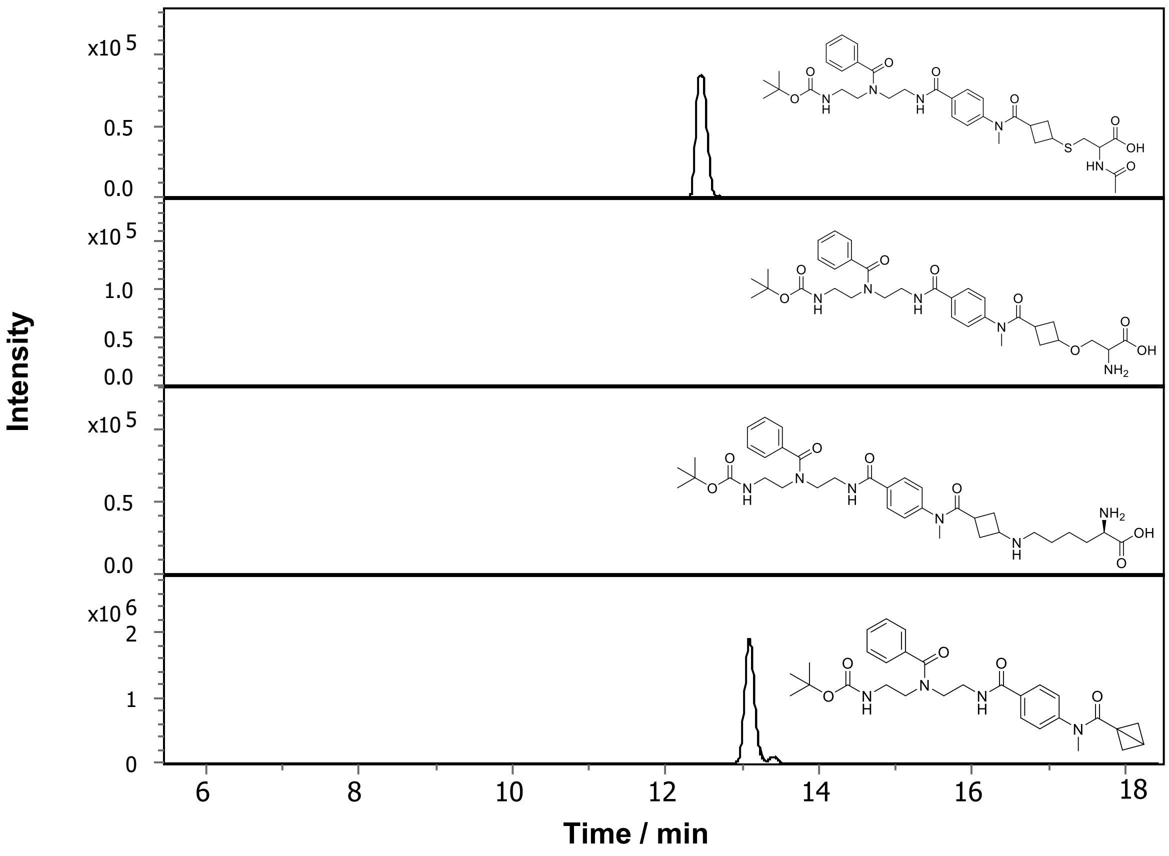

Figure S6. Extracted ion chromatograms of the conjugates detected in the chemoselectivity test demonstrating selectivity for thiols.

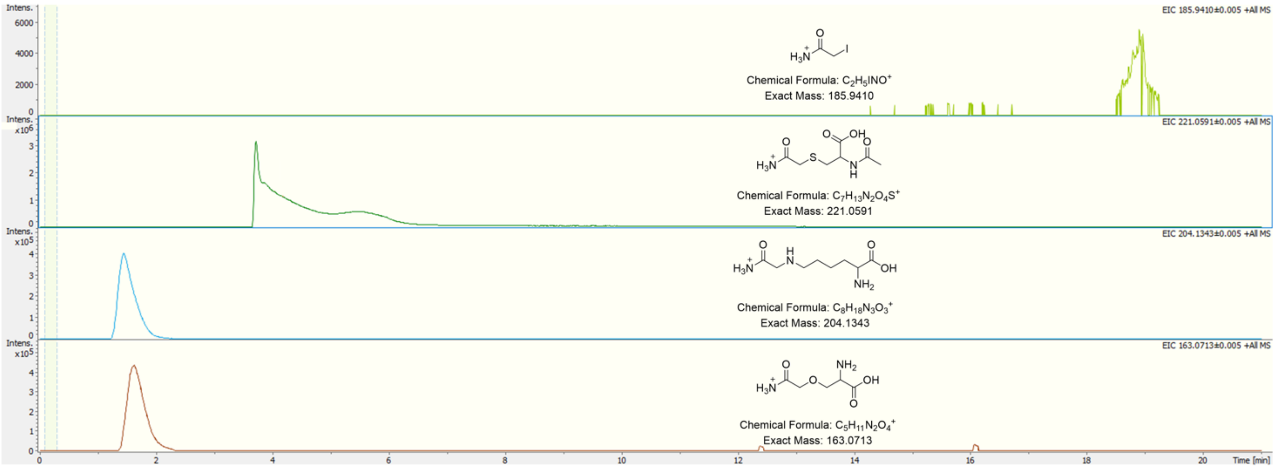

Figure S7. Extracted ion chromatograms of the conjugates detected in the chemoselectivity test using iodoacetamide.

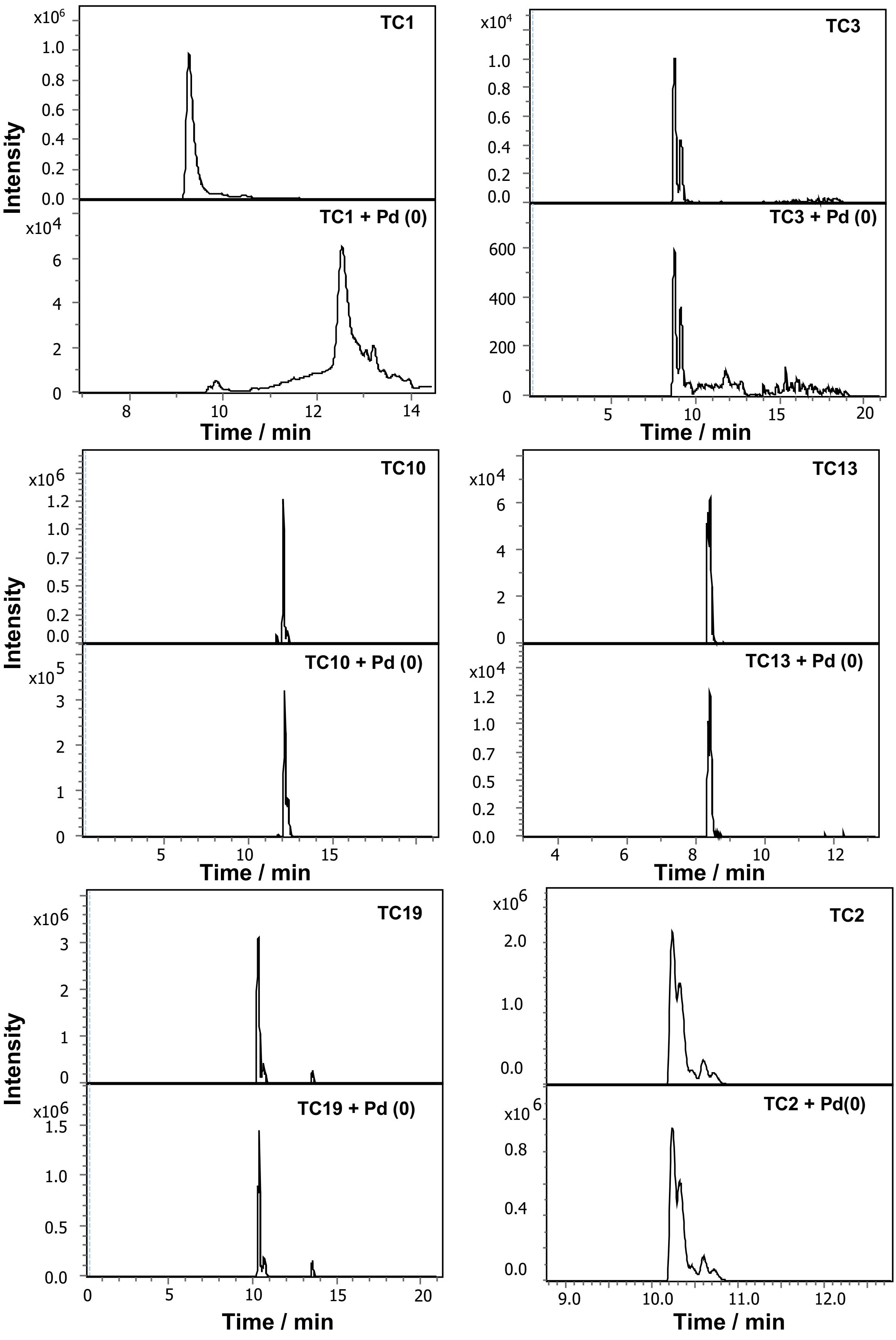

Figure S8. Extracted ion chromatograms of thiol conjugates before and after the stability test.

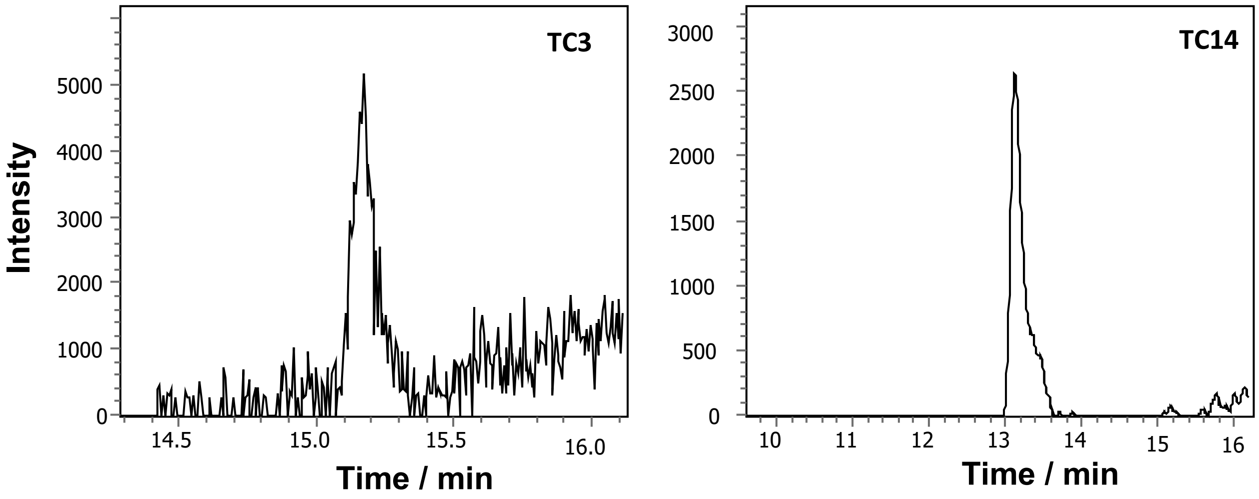

Figure S9. Extracted ion chromatograms of the captured cysteamine conjugate (**TC3**) and pantetheine (**TC14**),

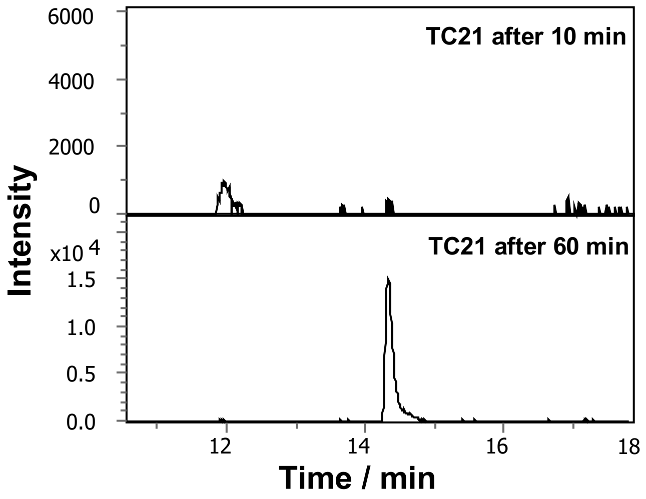

Figure S10. Extracted ion chromatograms of the synthetic cysteine persulfide conjugate **TC21** captured using probe **A**.

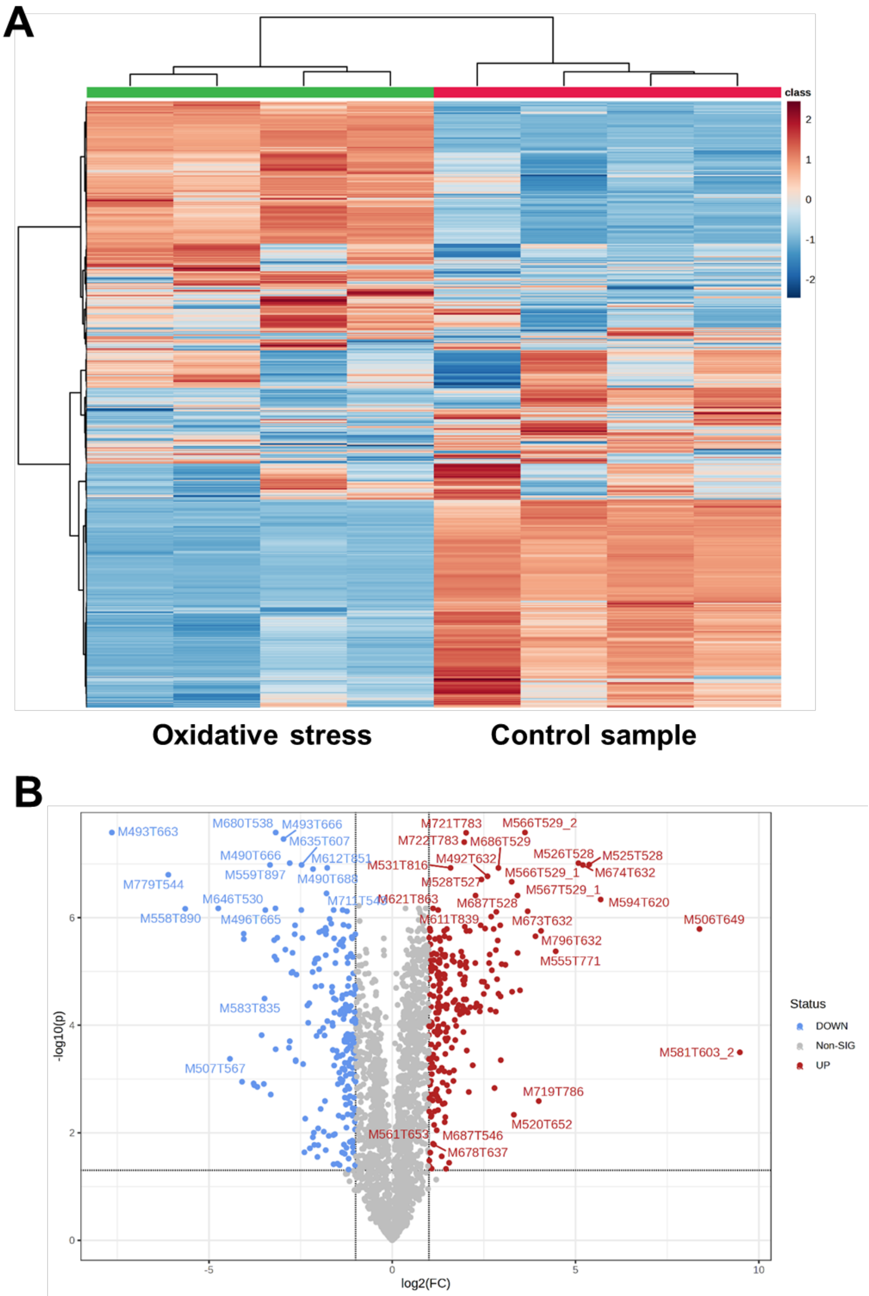

Figure S11. **A**) Heatmap of oxidative stress comparison experiment in *E. coli*; **B)** Volcano plot of oxidative stress comparison.

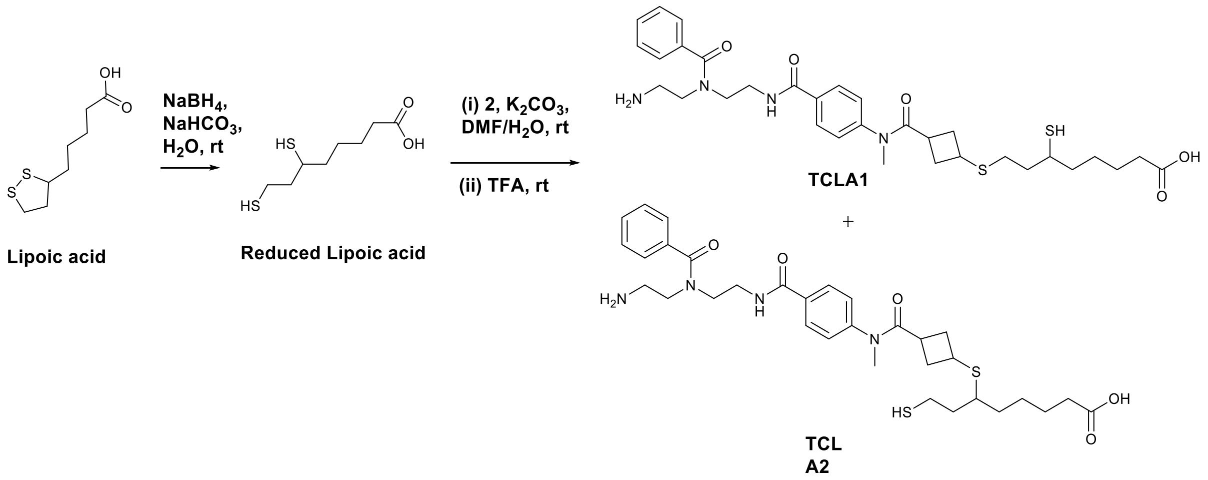

Scheme S1. Synthesis of standard lipoic acid probe conjugates **TCLA1** and **TCLA2** for the LC-MS based analysis.

2. Supporting Tables

Table S1. Features of thiol-containing metabolites in the human plasma.

The tentative chemical structure names are based on HMDB annotation (confidence level 3) and the correct structure without higher level of confidence can possibly be a regioisomer. Color indicators are related to the possible origin of metabolite according to HMDB database:

Color	Meaning
Blue	This compound is not a naturally occurring metabolite.
Green	Diet related.
Yellow	Naturally occurring metabolite.

Feature ID	Monoisotopic mass	Conjugate mass	retention time (min)	Metabolite name	HMDB MF
M485T711	63.9930	485.2165	11.84		CH4OS
M493T676	71.9965	493.2200	11.25	Thioacrolein	C3H4S
M497T724	76.0346	497.2552	12.07	1-Propanethiol	C3H8S
M498T653	77.0299	498.2424	10.87	Cysteamine	C2H7NS
M507T731	86.0121	507.2356	12.18	Divinyl sulfide/ 2,3-Dihydrothiophene	C4H6S
M513T578	92.0478	513.2713	9.63		C2H8N2S
M529T731	107.9942	529.2177	12.18	Vinylsulfonic Acid	C2H4O3S
M537T635	116.0479	537.2714	10.58		C4H8N2S
M543T733	122.0372	543.2607	12.21	Diethyl sulfone/ Thiodiglycol	C4H10O2S
M556T708	135.0354	556.2669	11.78	Homocysteine	C4H9NO2S
M572T692	151.0387	572.2622	11.53	Thioacetanilide	C8H9NS
M572T716	151.0638	572.2873	11.93		C5H13NO2S
M573T663	152.0156	573.2463	11.03	Mercaptopurine	C5H4N4S
M575T670_1	154.0378	575.2613	11.16	1-(5-Methyl-2-thienyl)-1-propanone	C8H10OS
M575T651	154.0380	575.2615	10.85	3-Acetyl-2,5-dimethylthiophene	C8H10OS
M577T625	156.0153	577.2388	10.42	Methyl phenyl disulfide	C7H8S2
M577T642_2	156.0538	577.2773	10.69	2-[(Isopropylthio)methyl]furan	C8H12OS
M587T767	166.0385	587.2620	12.78	2-Methyl-6-thiopurine	C6H6N4S
M587T767	166.0385	587.2620	12.78	6-Methylmercaptopurine	C6H6N4S
M589T620_2	168.0176	589.2411	10.32	(Phenylthio)acetic acid	C8H8O2S
M603T662	182.0698	603.2933	11.03		C5H14N2O3S
M605T721	184.0489	605.2724	12.01	(+/-)-3-[(2-methyl-3-furyl)thio]-2-butanone	C9H12O2S
M607T602	186.0279	607.2514	10.02		C8H10O3S
M609T767	188.0206	609.2441	12.78		C7H8O4S
M609T569	188.0439	609.2674	9.47	2-(Sec-butyldisulfanyl)-1h-imidazole	C7H12N2S2
M609T600	188.0436	609.2671	9.99	2-(Sec-butyldisulfanyl)-1h-imidazole	C7H12N2S2

M619T758	198.0651	619.2886	12.71	4-[(2-Furanylmethyl)thio]-2-pentanone	C10H14O2S
M625T767	203.9946	625.2181	12.78		C4H4N4O4S
M627T721	206.0310	627.2545	12.02		C5H10N4OS2
M641T758	220.0469	641.2704	12.70		C7H12N2O4S
M647T643	226.0960	647.3195	10.72	3-[(2-Methyl-3-furanyl)thio]-4-heptanone	C12H18O2S
M649T646	228.0752	649.2987	10.69	Antigastrin/ Phosphonol	C13H12N2S/ C6H17N2O3PS
M652T753	231.0902	652.3137	12.28	L-Proline, 1-(2-methyl-3-(methylthio)-1-oxopropyl)-, (S)-	C10H17NO3S
M653T727	232.0867	653.3102	12.12		C10H16O4S
M669T708_2	248.0805	669.3040	11.72	(5'-methylthio)pentylmalate	C10H16O5S
M688T647	267.1590	688.3825	10.77		C10H25N3O3S
M699T625	278.1300	699.3103	10.41	Pantetheine	C11H22N2O4S
M717T576	296.0975	717.3210	9.60	Bentazepam	C17H16N2OS
M724T771	303.1591	724.3826	12.85	1-Oxononan-4-yl (2R)-2-acetamido-3-sulfanylpropanoate	C14H25NO4S
M727T668	306.0607	727.2842	11.13	Monoacetyldapsone hydroxylamine	C14H14N2O4S
M729T690	308.0760	729.2995	10.85	phenylacetohydroximoyl-cysteinylglycine	C13H14N3O4S
M731T664	310.0919	731.3154	11.05		C13H18N4OS2
M731T614	310.1126	731.3361	10.23	Dibutyl sulfosuccinate	C12H22O7S
M731T626	310.1114	731.3349	10.43	Dibutyl sulfosuccinate	C12H22O7S
M747T641	326.0870	747.3105	10.67		C12H22O6S2
M751T652	330.0583	751.2818	10.85		C6H14N6O8S
M757T715	336.1078	757.3313	11.91	Firocoxib	C17H20O5S
M757T677	336.1079	757.3314	11.27	Firocoxib	C17H20O5S
M773T677	352.1030	773.3265	11.28	Hexythiazox/ Penicilloic acid	C17H21ClN2O2S/ C16H20N2O5S
M785T785	364.1397	785.3632	13.09	Pyridaben/ Desacetyl-alacepril	C19H25ClN2OS/ C18H24N2O4S
M787T708	366.1182	787.3417	11.78	Ilaprazole	C19H18N4O2S
M789T642	368.0979	789.3214	10.69	Phenoxomethylpenicilloyl	C16H20N2O6S
M798T718	377.0869	798.3104	11.96	S-Pyruvylglutathione	C13H19N3O8S
M817T653	396.1289	817.3524	10.88		C17H24N4O3S2
M818T624	396.1287	817.3522	10.39		C17H24N4O3S2
M841T625	420.2168	841.4403	10.40		C24H36O2S2
M863T625	442.1989	863.4224	10.40		C17H34N2O9S
M879T633	458.0831	879.3066	10.54		C15H18N6O9S

Table S2. Features of thiol-containing metabolites in the pooled human feces.

The tentative chemical structure names are based on HMDB annotation (confidence level 3) and the correct structure without higher level of confidence can possibly be a regioisomer. Color indicators are related to the possible origin of metabolite according to HMDB database:

- (blue) This compound is not a naturally occurring metabolite.
- (green) Diet related.
- (yellow) Naturally occurring metabolite.

Feature ID	Monoisotopic mass	Conjugate mass	retention time (min)	Metabolite name	HMDB MF
M483T726	61.9066	483.1301	12.11		
M485T480_2	63.9650	485.1885	7.98		
M485T669	63.9932	485.2167	11.76		CH4OS
M493T687	71.9968	493.2203	11.44	Thioacrolein	C3H4S
M513T586	92.0482	513.2717	9.77		C2H8N2S
M529T726	107.9945	529.2180	12.11		C2H4O3S
M542T793	121.0197	542.2746	13.21	Cysteine	C3H7NO2S
M543T755	122.0378	543.2613	12.42		C4H10O2S
M543T766	122.0377	543.2612	12.75		C4H10O2S
M543T755	122.0378	543.2613	12.59		C4H10O2S
M551T691	130.0640	551.2875	11.52	6-Methyl-5,6-dihydro-4H-1,3-thiazin-2-amine	C5H10N2S
M553T558	131.9817	553.2052	9.3		C3H4N2S2
M556T720	135.0354	556.2674	11.99	Homocysteine	C4H9NO2S
M572T703	151.0392	572.2627	11.73	Thioacetanilide	C8H9NS
M575T675	154.0389	575.2624	11	1-(5-Methyl-2-thienyl)-1-propanone	C8H10OS
M575T622	154.0387	575.2622	10.37	3-Acetyl-2,5-dimethylthiophene	C8H10OS
M575T634	154.0388	575.2623	10.56	1-(2-Thienyl)-1-butanone	C8H10OS
M575T692	154.0387	575.2622	11.25		C4H10O4S
M577T651	156.0542	577.2777	10.83	2-[(Isopropylthio)methyl]furan	C8H12OS
M587T766	166.0386	587.2621	12.74	2-Methyl-6-thiopurine	C6H6N4S
M593T612	172.0491	593.2726	10.2		C4H8N6S
M593T628	172.0492	593.2727	10.47		C4H8N6S
M593T668	172.0495	593.2730	11.14		C4H8N6S
M603T671	182.0703	603.2938	11.19		CH10N8OS
M605T717	184.0496	605.2731	11.96	(+/-)-3-[(2-methyl-3-furyl)thio]-2-butanone	C9H12O2S
M609T766	188.0207	609.2442	12.76		C7H8O4S
M609T611	188.0442	609.2677	10.15	2-(Sec-butyldisulfanyl)-1h-imidazole	C7H12N2S2
M615T549	193.9432	615.1667	9.14		C3H2N2O4S2
M621T550	199.9926	621.2161	9.17		C3H8N2O4S2

M631T763	209.9096	631.1331	12.72		H2O9S2
M641T756	220.0470	641.2705	12.68		C7H12N2O4S
M645T791	224.0813	645.3048	13.18	Propyl 1-(propylthio)propyl disulfide	C9H20S3
M647T651	226.0963	647.3198	10.85	3-[(2-Methyl-3-furanyl)thio]-4-heptanone	C12H18O2S
M651T669	230.1072	651.3307	11.16	Dicyclohexyl disulfide	C12H22S2
M652T758	231.0908	652.3143	12.63	1-(2-methyl-3-(methylthio)-1-oxopropyl)-	C10H17NO3S
M655T727	233.9347	655.1582	12.11		C6H2O6S2
M661T397	239.9467	661.1702	6.62		C7N2O6S
M667T791	246.0631	667.2866	13.18	S-Nitrosocaptopril/ 9-Deaza-9-(3-thienylmethyl)guanine	C9H14N2O4S
M699T634	278.1300	699.3109	10.54	Pantetheine	C11H22N2O4S
M709T980	288.1203	709.3438	16.33	Soterenol	C12H20N2O4S
M717T585	296.0986	717.3221	9.75	Bentazepam	C17H16N2OS
M729T647	308.0770	729.3005	10.73	Dansylglycine/ phenylacetohydroximoyl-cysteinylglycine	C14H16N2O4S
M774T688	352.1036	773.3271	11.47	Hexythiazox	C17H21ClN2O2S
M798T758	377.0878	798.3113	12.25	S-Pyruvylglutathione	C13H19N3O8S

Table S3. Features only detected in the control samples compared to the oxidative stress sample.

Ctrl	Conjugated mass	RT
M493T663	493.0626	11.06
M779T544	779.1509	9.07
M507T567	507.2501	9.44
M742T662	742.3156	11.03
M590T707	590.0541	11.79
M595T707	595.0536	11.79
M611T557	611.2542	9.28
M583T835	583.0559	13.91

Table S4. Features only detected in the oxidative stress sample.

OS	Conjugated mass	RT
M506T649	506.1175	10.82
M520T652	520.2656	10.87
M526T528	526.1745	8.80
M548T527_1	548.1085	8.79
M555T771	555.3034	12.85
M566T529_2	566.1390	8.82
M581T603_2	581.2938	10.05
M594T620	594.3142	10.33
M673T632	673.3456	10.54
M719T786	719.1918	13.10
M738T528	738.1378	8.81
M795T632	795.4018	10.53

M894T651	894.3784	10.85

Table S5. Semi-quantitative analysis of GSH in the comparison between control samples and oxidative stress samples through comparison of an internal standard.

**AUC**	**Ctrl-1**	**Ctrl-2**	**Ctrl-3**	**Ctrl-4**	
**GSH**	35654	41399	45417	33873	
**IS**	651750	516590	463422	860325	**Mean**
**Ratio**	0.054705	0.080139	0.098004	0.039372	0.068055

**AUC**	**OS-1**	**OS-2**	**OS-3**	**OS-4**	
**GSH**	20058	17490	19998	23349	
**IS**	653478	610691	530855	870360	**Mean**
**Ratio**	0.030694	0.02864	0.037671	0.026827	0.030958

Table S6. Detected Top100 features from bacteria lysates with intensities.

m/z	p.value	FDR	Salmonella-1	Salmonella-2	Salmonella-3	Salmonella-4	Shigella_1	Shigella_2	Shigella_3	Shigella_4	E.coli_1	E.coli_2	E.coli_3	E.coli_4
574.2154	7.28E-20	1.33E-16	310341.75	310271.57	311239.96	310336.52	52104.20	52292.37	51736.64	51682.96	349699.06	352972.32	353739.17	350634.38
816.4107	1.88E-18	1.72E-15	301634.50	306535.80	307700.98	308910.41	6351.54	5222.96	6168.30	5663.91	462838.43	458495.94	457812.63	457785.94
815.4073	3.28E-18	2.00E-15	639293.30	642874.57	646652.70	643348.38	11979.32	11763.80	12332.68	12797.66	973021.93	987756.34	979723.26	992620.36
558.2414	2.29E-17	9.25E-15	5637974.39	5677237.15	5632748.13	5680283.04	805020.29	793383.77	790970.45	802026.68	6433519.31	6524083.51	6473879.83	6587086.45
553.2951	2.53E-17	9.25E-15	393414.30	400784.61	399367.30	401477.35	7458.62	10770.81	6105.01	5304.61	591208.09	578995.33	582664.08	587147.67
515.3390	4.77E-17	1.45E-14	7127510.31	7138006.14	7118841.96	7155885.38	30704.70	29254.00	29947.10	30038.40	13543565.21	13549737.28	13474675.99	13855009.68
536.2595	6.61E-17	1.73E-14	3633401.38	3706466.96	3684780.90	3759647.80	326401.73	310705.94	317452.00	311084.35	5235936.09	5144359.83	5189461.22	5202272.62
516.3420	1.10E-16	2.51E-14	1838142.16	1829778.31	1845902.34	1794622.47	7709.47	8458.45	8290.25	9157.34	3684782.47	3661756.20	3679150.74	3587892.27
537.3207	1.51E-16	3.06E-14	6651147.55	6662970.56	6637853.78	6620528.78	66354.40	66285.78	65059.89	69081.86	9736876.25	9886080.44	9757827.93	10043055.62
554.2981	1.76E-16	3.22E-14	108557.06	110579.31	110109.34	112145.36	7210.50	8524.54	8873.51	8127.09	158220.13	158581.87	155786.28	159191.56
539.3271	3.78E-16	6.29E-14	248137.72	246376.43	244036.92	246011.00	2666.07	3545.08	3739.65	2881.89	364460.36	371372.92	371415.65	360041.76
553.7589	1.38E-15	2.10E-13	24197.52	25015.24	25357.14	24945.96	0.00	117.33	98.52	112.75	47835.65	46598.59	47722.20	46524.50
557.6790	1.81E-15	2.54E-13	9097.52	8749.85	9282.21	9536.21	115507.85	111414.81	115248.04	114494.91	25100.31	27190.00	25546.36	25598.91
517.3449	2.30E-15	3.01E-13	259552.32	261975.70	263870.65	257148.50	7955.18	8631.71	9095.82	1077.90	506925.70	506409.88	499936.78	489183.43
537.2627	2.88E-15	3.51E-13	1251052.46	1261258.39	1268383.70	1247369.10	109304.06	112149.83	110799.08	112033.29	1864556.30	1840154.49	1852783.66	1788792.96
604.2980	4.40E-15	5.03E-13	26868.74	25522.31	25405.12	25042.21	221959.64	224860.14	224777.98	228674.59	138075.82	131155.44	136380.98	136201.33
647.3415	5.89E-15	6.15E-13	25106.24	24163.67	22730.23	23236.91	22898.93	22368.45	21665.38	23522.77	154941.65	152416.54	151274.49	157948.23
684.3936	6.05E-15	6.15E-13	1114.64	1283.95	1318.35	1824.55	53201.14	53989.40	53441.62	51873.41	10612.86	11322.55	11848.65	12226.97
560.2480	6.39E-15	6.15E-13	303675.37	305875.78	307694.92	304049.08	51966.60	50861.13	51485.34	52926.35	369633.44	368837.89	367604.60	354994.28
696.2081	7.60E-15	6.95E-13	14410.11	16038.99	14923.46	13905.95	3464.81	3225.52	3701.25	3729.42	109259.82	110029.52	114723.16	112059.14
538.2661	8.76E-15	7.63E-13	227457.78	227843.24	227124.13	230657.53	19789.61	20897.48	19805.66	20347.72	339932.04	331153.82	341561.39	327040.69
508.2513	1.92E-14	1.59E-12	633.15	1259.09	874.57	1071.40	53834.38	56651.99	54265.37	55364.31	7582.59	7550.22	7426.05	8794.68
554.7585	2.50E-14	1.99E-12	12156.15	12386.33	12490.25	12599.68	0.00	0.00	96.51	0.00	24607.05	23495.47	24580.03	24753.39
491.2173	2.74E-14	2.09E-12	7229.00	6126.09	6594.47	7013.56	87789.93	89509.68	92857.76	90218.99	24315.33	23229.04	24360.55	24446.28
479.2291	3.51E-14	2.57E-12	46821.35	47515.06	49690.75	46229.57	7745.08	7884.26	7833.13	7643.32	68661.03	68558.93	69128.99	67796.17
543.2580	3.67E-14	2.59E-12	81304.25	78335.94	77290.52	76725.98	10868.93	10175.81	10053.29	10487.29	108802.90	110195.00	110193.44	106692.78
697.2068	3.82E-14	2.59E-12	17647.08	19002.54	18707.32	19100.75	2412.35	2404.72	1784.88	2481.83	135219.21	142572.80	142916.61	143625.13
506.3052	5.07E-14	3.31E-12	0.00	0.00	223.02	0.00	15071.54	14391.57	15005.25	14332.98	1837.38	1434.64	1676.02	1728.57
569.8405	6.42E-14	4.05E-12	5609.64	6005.79	4772.15	5913.47	48118.16	48218.82	49430.27	50340.42	14386.55	14078.32	14169.19	13304.94
543.8002	7.20E-14	4.39E-12	9996.02	9086.77	9275.85	9093.82	0.00	98.68	0.00	108.24	21828.17	21409.49	22452.69	22144.31
699.2065	8.24E-14	4.86E-12	15785.16	15188.46	15654.37	15976.32	1333.04	1944.90	1351.03	1569.58	112542.62	120094.30	120009.24	119098.75
559.2447	8.56E-14	4.90E-12	1752508.16	1769793.30	1771210.43	1808163.42	284408.81	281170.09	288278.25	289478.44	2160256.82	2112991.55	2126311.73	2039760.07
518.3477	1.31E-13	7.24E-12	28249.08	27252.76	27408.58	27755.42	3372.73	4078.53	3766.19	3604.69	52662.88	52021.58	53999.76	50701.44
685.2521	1.64E-13	8.61E-12	2762.67	1442.69	2365.69	2364.54	27978.87	27024.23	27263.79	27820.40	5431.09	4590.37	4322.24	4622.05
555.2956	1.65E-13	8.61E-12	36309.06	36197.13	37209.24	37317.87	5128.89	4020.80	4718.98	5495.16	49638.87	51494.43	51819.81	52416.44
504.2453	1.87E-13	9.49E-12	0.00	103.25	0.00	0.00	27841.57	25780.06	27226.93	27382.23	2442.53	2175.58	2875.66	2637.95
642.3459	1.99E-13	9.68E-12	1899.86	1269.65	1242.25	1052.38	27009.71	27951.98	27537.02	26372.90	6623.94	7412.73	7036.17	6631.79
767.1938	2.01E-13	9.68E-12	920.94	884.33	1229.79	876.93	446.92	194.80	224.84	200.29	10400.25	10752.29	10804.42	10174.70

m/z	p.value	FDR	Salmonella-1	Salmonella-2	Salmonella-3	Salmonella-4	Shigella_1	Shigella_2	Shigella_3	Shigella_4	E.coli_1	E.coli_2	E.coli_3	E.coli_4
575.2185	2.11E-13	9.91E-12	103527.70	106861.19	105191.44	109441.15	23699.42	21609.57	25372.99	21978.50	119259.05	121889.29	121826.14	119693.96
700.2091	3.10E-13	1.42E-11	5977.48	5339.15	6095.26	6059.88	484.37	294.15	433.23	319.92	40587.45	42664.69	41457.78	43958.62
502.2141	4.79E-13	2.14E-11	30436.54	29914.52	30785.01	30575.56	7680.50	6376.03	6794.48	6579.40	38073.97	38125.13	37567.06	39690.02
560.2930	5.25E-13	2.19E-11	1387.58	1751.72	2204.75	2413.87	50664.81	51295.42	47945.50	48169.46	10710.02	10159.10	10400.28	10736.92
659.3405	5.35E-13	2.19E-11	2001.24	2316.98	2157.08	2682.56	38280.77	37433.58	40138.02	39755.23	11716.71	11330.28	11247.80	12025.22
441.2345	5.36E-13	2.19E-11	1444.88	1331.49	1120.39	1870.52	29149.81	31106.72	30452.77	29939.77	24077.89	22659.91	23172.10	23359.58
486.2923	5.40E-13	2.19E-11	0.00	0.00	93.66	0.00	18627.94	17046.14	17737.29	17774.99	1825.09	1408.72	1810.14	1351.15
759.4119	5.75E-13	2.29E-11	4251.56	2689.78	3670.88	4167.02	40814.86	42507.56	43085.52	42719.97	13991.31	13843.23	13335.17	15024.60
489.7844	6.16E-13	2.40E-11	0.00	0.00	0.00	194.03	16531.59	17192.33	17730.45	17259.75	2161.15	2153.88	1376.90	2419.73
687.1278	6.64E-13	2.53E-11	7557.33	7658.88	7725.24	7794.17	1656.26	1538.71	1761.20	1914.15	756.93	1127.39	1151.36	1103.99
559.6797	6.86E-13	2.56E-11	3102.34	2843.17	3891.32	1941.03	49059.24	46757.54	49910.30	48616.63	11169.83	10984.51	12795.90	11949.11
521.2759	8.74E-13	3.20E-11	24479.38	23519.27	23868.49	22950.85	5148.60	5518.88	5086.87	5265.39	35436.27	33457.28	34059.69	33802.11
719.2808	9.09E-13	3.25E-11	11283.29	11900.05	12057.71	12137.71	1049.40	503.93	653.42	983.04	26856.38	25258.50	26546.94	25321.09
943.3336	9.23E-13	3.25E-11	211.48	225.40	291.17	419.95	4458.59	4330.18	4424.87	4275.18	658.49	475.00	719.53	522.34
558.6791	9.75E-13	3.37E-11	7144.43	6844.40	5892.29	7686.25	94412.86	90414.71	87120.77	88141.73	20099.62	20878.54	21112.82	21413.56
698.2093	1.00E-12	3.39E-11	5661.01	6566.83	6789.46	6742.16	103.30	620.26	307.07	417.72	45071.66	47516.24	47439.38	49613.34
634.3051	1.03E-12	3.41E-11	13452.26	13061.54	13099.87	12569.56	4151.64	3884.50	4307.52	3732.89	1258.47	1369.66	1232.69	1534.89
470.2975	1.16E-12	3.78E-11	678.78	534.36	521.40	789.98	23434.18	23271.70	22453.64	22624.37	2823.60	3967.83	2085.96	3385.43
843.2016	1.37E-12	4.41E-11	1747.15	2369.17	2593.25	3275.30	18920.42	18530.17	18953.82	18699.39	5862.39	5608.35	5816.22	5939.88
539.2696	1.40E-12	4.42E-11	28639.85	29598.54	29332.73	30196.10	1822.34	4775.54	2923.52	2501.22	41177.21	42746.34	42279.51	41840.16
628.3304	1.72E-12	5.32E-11	1616.16	1595.64	1887.02	1607.61	18493.98	18358.47	17846.83	18874.61	6452.92	7187.78	7345.82	7687.63
675.3143	2.34E-12	7.12E-11	7831.07	7895.98	7688.87	7867.64	1125.79	910.44	1001.45	799.66	1005.58	535.94	1216.66	839.67
697.2987	2.55E-12	7.65E-11	6488.87	6461.94	6129.75	5876.24	2124.19	1855.93	2967.04	1933.77	18392.14	19306.22	18493.65	18537.42
534.8141	2.77E-12	8.17E-11	1480.00	1540.20	1252.10	1452.80	0.00	110.23	95.14	0.00	4854.47	5203.31	5148.80	5246.22
632.2983	4.24E-12	1.23E-10	121566.14	126280.15	130354.55	120061.78	41109.46	37271.20	36478.84	37359.53	14514.34	15513.76	14200.45	13995.78
696.2846	4.60E-12	1.32E-10	23132.78	23865.56	25176.18	25235.78	3354.43	3510.21	3140.01	3117.28	25237.23	26241.05	26072.78	25862.16
682.1248	4.95E-12	1.39E-10	11703.48	11267.32	11705.64	11343.46	2685.98	3434.64	3347.06	3271.12	1175.96	1416.93	1605.59	1779.58
638.1850	5.11E-12	1.42E-10	18291.68	19779.58	19453.89	19820.97	4078.31	2844.46	3745.06	3026.42	22783.24	23269.03	23209.10	23212.03
479.2291	5.32E-12	1.45E-10	36483.80	35572.58	32915.74	34168.96	7586.77	8087.27	7291.56	7353.98	50655.67	49179.51	50187.25	48199.13
841.2024	5.98E-12	1.61E-10	3178.55	2491.91	2833.89	3231.12	24698.67	22600.28	23781.53	23109.06	6838.28	6778.54	7355.55	6731.92
753.2994	6.22E-12	1.65E-10	694.35	881.54	531.08	536.17	702.88	190.20	827.28	678.33	10919.20	11071.51	10090.86	10680.84
#######	7.03E-12	1.84E-10	337.44	411.79	424.03	418.27	10284.96	10230.90	10475.89	10241.37	892.73	1759.29	1412.54	2048.07
633.3018	7.28E-12	1.88E-10	50711.59	55392.73	54893.63	53383.41	17984.90	17027.33	15827.80	16345.32	6382.45	7116.84	7057.91	6425.91
821.4402	7.75E-12	1.95E-10	150.13	675.31	385.61	103.26	12548.32	12776.48	12712.02	13756.78	1917.50	1378.86	1708.84	1376.49
583.3279	7.79E-12	1.95E-10	211.40	92.18	105.41	95.17	8180.79	7854.56	7580.56	7802.82	655.83	518.98	1217.39	579.59
501.2110	7.95E-12	1.97E-10	98762.16	98433.55	100806.19	95658.29	17302.41	17092.15	16711.85	20187.83	134188.01	131521.06	135371.99	124405.22
#######	9.97E-12	2.43E-10	1162.92	843.06	959.49	1327.26	15399.65	14243.17	15041.78	15659.93	3027.22	3276.49	2949.41	2416.29
505.2044	1.12E-11	2.69E-10	12047.93	11752.10	12245.30	11836.92	13085.81	12018.01	12507.49	12992.23	50074.10	49923.18	46572.72	46533.64
697.2875	1.23E-11	2.93E-10	10829.45	10602.66	11023.48	10145.01	1424.25	1878.23	1172.45	1075.93	11228.31	10839.46	10960.75	10937.23

m/z	p.value	FDR	Salmonella-1	Salmonella-2	Salmonella-3	Salmonella-4	Shigella_1	Shigella_2	Shigella_3	Shigella_4	E.coli_1	E.coli_2	E.coli_3	E.coli_4
839.2022	1.35E-11	3.13E-10	91.63	391.88	384.23	407.30	7232.61	7265.22	6587.62	6900.73	1630.27	1616.00	1556.20	1714.54
688.1265	1.35E-11	3.13E-10	12822.90	13378.15	13278.36	12734.30	3717.48	3894.85	3387.43	4103.16	1559.35	1031.18	2072.23	1366.49
425.0440	1.39E-11	3.16E-10	5497.88	5966.26	6205.84	5968.94	21137.41	22109.11	22526.17	22075.40	8099.15	9006.40	9347.23	9237.57
842.2051	1.40E-11	3.16E-10	702.96	735.98	741.83	642.31	8908.74	9738.27	9399.30	8851.54	2885.19	2798.08	2518.46	2787.98
949.4322	1.46E-11	3.26E-10	3754.44	4229.86	3859.70	4173.37	14570.35	15287.49	15033.03	15971.89	4305.52	4024.61	4444.22	4327.32
686.1251	1.53E-11	3.37E-10	25810.93	26046.04	27270.30	26989.63	8081.00	8655.25	7594.66	8971.52	3767.14	3831.07	4794.29	5352.77
564.7208	1.59E-11	3.47E-10	13983.59	15655.34	14856.04	16290.44	201.57	0.00	143.41	0.00	22039.32	22935.23	23330.63	23528.49
993.3564	1.84E-11	3.96E-10	2442.41	2205.81	1803.37	2407.98	20605.51	21130.66	20732.99	21803.36	3909.03	5298.36	5906.75	5518.63
631.3279	1.96E-11	4.12E-10	118.51	0.00	125.55	98.39	6074.05	6344.30	6757.76	6009.19	382.70	506.24	362.07	644.77
561.4274	1.96E-11	4.12E-10	5561.67	5430.25	5668.20	5318.70	573.61	807.90	852.53	582.12	5493.43	5460.62	5178.67	5659.69
776.3454	2.00E-11	4.16E-10	0.00	0.00	98.74	93.35	10772.01	9712.19	10984.36	10737.79	2205.32	2436.99	2410.55	2465.75
471.3004	2.07E-11	4.25E-10	0.00	0.00	108.48	0.00	5253.22	6000.52	5697.71	5922.13	115.66	119.31	201.47	0.00
822.4432	2.11E-11	4.30E-10	1756.93	1979.71	1838.67	2236.32	5398.06	5613.04	5492.66	5341.05	711.77	835.67	931.51	805.41
544.1194	2.23E-11	4.48E-10	58609.53	66841.16	71400.27	76818.28	350579.28	339277.12	346836.06	376564.01	41224.60	44561.34	49103.17	49563.82
745.2965	2.65E-11	5.27E-10	9802.16	9666.72	9504.67	9245.17	195.31	540.65	403.22	440.23	13477.36	15092.96	14902.91	14294.54
464.1396	2.75E-11	5.41E-10	79654.13	85955.95	81154.73	87867.95	58562.14	56103.59	56227.99	58088.27	4660.26	3983.09	5888.11	7475.01
836.3278	2.87E-11	5.58E-10	464823.10	471604.76	471851.33	477076.06	107826.53	102390.00	105225.99	106214.84	667584.39	656182.54	605508.72	619250.64
534.3078	3.85E-11	7.42E-10	22130.24	20465.86	19892.30	20567.42	1632.95	1754.70	2169.10	1249.00	32651.97	30956.66	30488.56	29438.66
562.7862	4.09E-11	7.80E-10	105.51	266.00	320.56	302.76	9541.95	8921.28	9927.80	10198.73	1199.45	1070.19	1464.39	1403.66
495.0902	4.71E-11	8.85E-10	9316.46	10129.00	9986.77	10463.40	1961.36	1640.75	1662.45	2263.33	961.59	733.25	665.75	945.67
534.2690	4.74E-11	8.85E-10	13989.45	13523.85	14706.99	14580.20	1075.01	1026.95	527.31	664.08	15099.09	16093.11	16195.25	14732.73
874.3415	5.39E-11	9.96E-10	16777.55	16874.13	17220.53	16638.12	10489.60	10564.11	9002.23	9218.09	2229.89	2283.27	2037.28	2293.77

Table S7. Metadata of patients for the pooled fecal and plasma sample.

Patient	Gender	Age	MRI	Other
**Fecal**				
060	Male	52	NAD	
043	Male	62	BD-IPMN	Lynch Syndrome
056	Female	66	Cyst	
046	Female	37	NAD	Hepatomegaly and splenomegaly
063	Male	68	NAD	
068	Male	67	MF BD-1PMN	Polyarthritis
058	Female	62	MF BD-1PMN	
047	Female	53	BD-1PMN	
**Plasma**				
039	Female	52	N/A	N/A

NAD- No acute distress

IPMN- Intraductal Papillary Mucinous Neoplasm

BD- Branch Duct

MF- Multifocal

3. General

All reagents and solvents were purchased from Sigma-Aldrich or Fischer Scientific and were used without further purification. Mass spectrometry grade solvents were used for UHPLC-ESI-MS analysis. Solutions were concentrated *in vacuo* on a Heidolph. Thin Layer Chromatography (TLC) was performed on silica gel 60 F-254 plates. Visualization of the developed chromatogram was performed using fluorescence quenching. Chromatographic purification of products was accomplished using flash column chromatography on Merck silica gel 60 (40−63 μm). NMR spectra were recorded on Agilent 400 MHz spectrometer (1H NMR: 400 MHz, 13C NMR: 100 MHz). Chemical shifts are reported in parts per million (ppm) on the δ scale from an internal standard. Multiplicities are abbreviated as follows: s = singlet, d = doublet, t = triplet, q = quartet, m = multiplet. Glass vials used for handling magnetic beads were microwave vials from Biotage (0.2-0.5 mL or 0.5-2.0 mL). The UHPLC-MS/MS analysis was performed in a Maxis II ETD Q-TOF mass spectrometer using an electrospray ionization (ESI) source with an Elute UHPLC system and equipped with a Waters ACQUITY UPLC BEH C18 column (2.1 × 75 mm, 1.7 μm particle size) or Waters ACQUITY UPLC HSS T3 column (1.8 × 100 mm, 2.1 μm particle size). Milli-Q water with 0.1% formic acid was used as mobile phase A and LC-MS grade methanol with 0.1% formic acid was used as mobile phase B. The column temperature was kept at 40 °C, and the autosampler was kept at 4 °C. The flow rate was set to 0.22 mL/min. The gradient used was as follows: 0–2 min, 0% B; 2–15 min, 0–100% B; 15–16 min, 100% B; 16–17 min, 100-0% B; 17–23 min, 0% B. The system was controlled using the Compass HyStar software package from Bruker. High-resolution mass spectra were acquired in positive mode at a mass range of m/z 50–1200. Data acquisition was performed in MSE mode or AutoMSMS.

4. Synthetic procedures

*Preparation of compound **5***

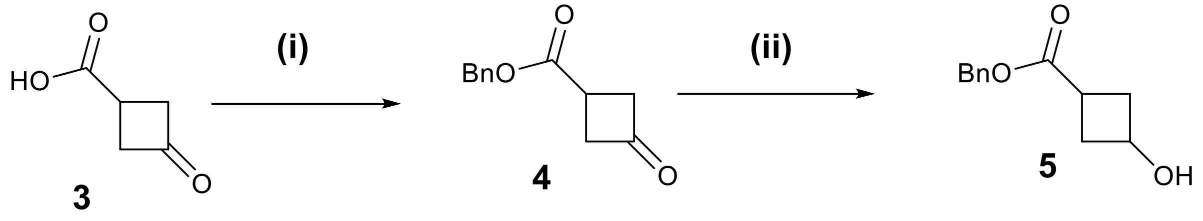

Compound **5** was prepared from the carboxylic acid **3** according to procedure described in literature.[1]

^{1}H NMR (400 MHz, $CDCl_3$) of **5**: δ 2.15-2.26 (m, 2H), 2.57-2.71 (m, 3H), 4.15-4.24 (m, 1H), 5.12 (s, 2H), 7.30-7.39 (m, 5H).

*Preparation of compound **6***

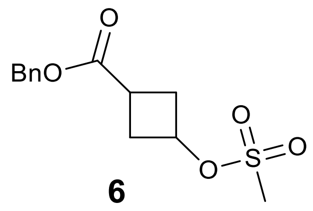

Benzyl 3-((methylsulfonyl)oxy)cyclobutane-1-carboxylate (6)

The solution of **5** (1.5 g, 7.47 mmol, 1 equiv.) in dry DCM (15 mL) was cooled to 0 ºC and triethylamine (1.56 mL, 11.20 mmol, 1.5 equiv.) was added. Methanesulfonyl chloride (693 uL, 8.96 mmol, 1.2 equiv.) was added dropwise at 0 ºC. This reaction mixture was stirred under nitrogen for 30 minutes while allowing it to warm to room temperature. The TLC analysis at this point showed the complete exhaustion of the starting material. Half-saturated sodium bicarbonate was added and the aqueous phase was extracted four times with DCM. The combined organic layers were dried over Na_2SO_4, filtered and concentrated *in vacuo*. The yellow oil was column chromatographed on silica gel (gradient 5% to 25% ethyl acetate/hexane) to give a slight yellow solid of **6** (1.8 g). Yield = 85%.

^{1}H NMR (400 MHz, $CDCl_3$): δ 2.55-2.64 (m, 2H), 2.67-2.84 (m, 3H), 2.99 (s, 3H), 4.89-4.97 (m, 1H), 5.14 (s, 2H), 7.31-7.40 (m, 5H).

Preparation of compound 7

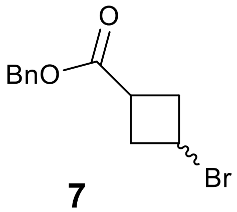

7

Benzyl 3-bromocyclobutane-1-carboxylate (7)

A mixture of **6** (1.8 g, 6.33 mmol, 1 equiv.) and lithium bromide (713 mg, 8.21 mmol, 1.3 equiv.) in dry DMF (20 mL) was stirred overnight at 80 ºC. The TLC analysis of the reaction mixture in toluene: acetone (3:1) indicated the complete exhaustion of the starting material. The reaction was cooled down to the room temperature. Saturated sodium bicarbonate was added and the mixture was stirred for 30 minutes at room temperature. The aqueous layer was extracted thrice with ethyl acetate. The combined organic layers were dried over Na_2SO_4, filtered and concentrated *in vacuo*. The yellow oil was column chromatographed on silica gel (gradient 1 % to 2 % ethyl acetate/hexane) to give an oil of **7** (559 mg). Yield = 33 %. The H-NMR indicated the product **7** contained the diastereomers in the ratio of 2:5.

^{1}H NMR (400 MHz, $CDCl_3$) of diastereomeric mixture: δ 2.67- 3.06 (m, 7H), 3.39-3.47 (m, 0.43H), 4.34-4.42 (m, 1H), 4.62-4.69 (m, 0.43H), 5.13-5.14 (m, 3H), 7.31-7.40 (m, 7H).

^{1}H NMR (400 MHz, $CDCl_3$) of major diastereomer: δ 2.73- 2.91 (m, 4H), 2.98- 3.06 (m, 1H), 4.34-4.42 (m, 1H), 5.13 (s, 2H), 7.31-7.40 (m, 5H).

^{1}H NMR (400 MHz, $CDCl_3$) of minor diastereomer: δ 2.67- 2.74 (m, 2H), 2.91-2.97 (m, 2H), 3.40-3.47 (m, 1H), 4.63-4.70 (m, 1H), 5.14 (s, 2H), 7.32- 7.39 (m, 5 H).

*Preparation of compound **8***

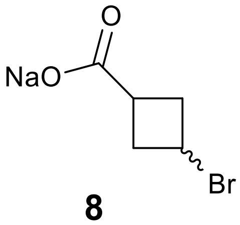

8

Sodium 3-bromocyclobutane-1-carboxylate (8)

To the solution of **7** (559 mg, 2.08 mmol, 1 equiv.) in THF (7 mL) was added 5N NaOH (420 µL) and the mixture was stirred overnight at 45 ºC. The mixture was cooled to room temperature and the volatiles were removed *in vacuo* to give an off-white solid of **8**. This solid was used in the next step without further purification.

^{1}H NMR (400 MHz, CD_3OD): δ 2.52-2.68 (m, 3.33H), 2.77-2.93 (m, 4.20H), 3.28-3.36 (m, 0.33H), 4.45-4.53 (m, 1H), 4.63-4.70 (m, 0.40 H).

Preparation of compound **10**

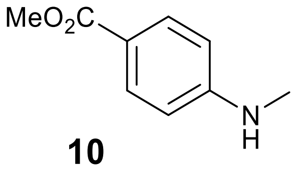

10

Methyl 4-(methylamino)benzoate (10)

A mixture of 4-(methylamino)benzoic acid (1.0 g, 6.62 mmol, 1 equiv.) and 4-methylbenzenesulfonic acid (1.5 g, 8.71 mmol, 1.3 equiv.) in methanol (20 mL) was refluxed overnight at 70 ºC. The TLC analysis of the reaction mixture indicated the complete exhaustion of the starting material after 16 h of refluxing. Methanol was removed *in vacuo*. Saturated sodium bicarbonate was added and the aqueous layer was extracted thrice with ethyl acetate. The combined organic layers were dried over Na_2SO_4, filtered and concentrated *in vacuo*. The residue was column chromatographed on silica gel (gradient 5 % to 10 % ethyl acetate/hexane) to give a slight yellow solid of **10** (808 mg). Yield = 74%.

^{1}H NMR (400 MHz, $CDCl_3$): δ 2.88 (s, 3H), 3.85 (s, 3H), 6.55 (d, J = 9.2 Hz, 2H), 7.87 (d, J = 8.8 Hz, 2H).

*Preparation of compound **9***

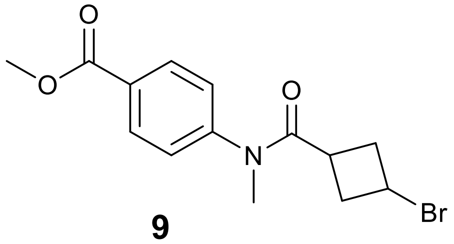

9

Methyl 4-(3-bromo-N-methylcyclobutane-1-carboxamido) benzoate (9)

To a stirred solution of **8** (700 mg, 6.13 mmol, 1.2 equiv.) in dry DMF (15 mL) was added propanephosphonic acid anhydride (50 wt% in ethyl acetate, 14 mL, 22.25 mmol, 7 equiv.) and *N,N*- diisopropylethylamine (4 mL, 22.25 mmol, 7 equiv.). Methyl 4-(methylamino) benzoate **10** (525 mg, 3.18 mmol, 1 equiv.) in ethyl acetate (15 mL) was added. This reaction solution was refluxed overnight at 80 ºC. The TLC and LCMS analyses after the overnight stirring indicated the presence of both the starting material and the desired product. The reaction was allowed to cool down to room temperature and saturated sodium bicarbonate was added. This mixture was stirred for 30 minutes at room temperature. The aqueous layer was extracted five times with ethyl acetate. The combined organic layers were dried over Na_2SO_4, filtered and concentrated *in vacuo*. The residue was column chromatographed on silica gel (gradient 5% to 12.5% ethyl acetate/petroleum ether) to give a yellow oil of **9** (538 mg). Yield = 54%.

^{1}H NMR (400 MHz, $CDCl_3$): δ 2.30-2.52 (m, 2H), 2.73-2.80 (m, 3H), 3.28 (s, 3H), 3.94 (s, 3H), 4.11-4.20 (m, 1H), 7.19 (d, *J* = 8.4 Hz, 2H), 8.09 (d, *J* = 8.6 Hz, 2H).

^{13}C NMR (100 MHz, $CDCl_3$): δ 35.5, 37.2, 37.6, 38.7, 52.5, 127.2, 131.3, 147.5, 166.2, 171.8.

HRMS (ESI) m/z $[M+H]^+$ calcd for $C_{14}H_{17}BrNO_3$ 326.0386; Found 326.0389.

*Preparation of compound **11***

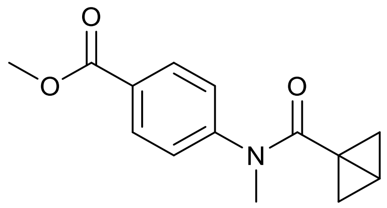

11

Methyl 4-(N-methylbicyclo[1.1.0]butane-1-carboxamido)benzoate (11)

To a stirred solution of **9** (538 mg, 1.65 mmol, 1 equiv.) in dry toluene (10 mL) was added lithium bis(trimethylsilyl)amide (1M in THF, 11.3 mL) at 0 ºC. This mixture was stirred under nitrogen for 1 h at 0 ºC. The TLC analysis of the reaction mixture indicated the complete exhaustion of the starting material. Saturated ammonium chloride was added and the aqueous layer was extracted three times with ethyl acetate. The combined organic layers were dried over Na_2SO_4, filtered and concentrated *in vacuo*. The residue was column chromatographed on silica gel (gradient 5 % to 20 % ethyl acetate/hexane) to give an off-white solid of **11** (345.8 mg). Yield = 85%.

^{1}H NMR (400 MHz, $CDCl_3$): δ 0.85 (d, *J* = 2.8 Hz, 2H), 1.87 (d, *J* = 3.2 Hz, 2H), 2.08-2.11 (m, 1H), 3.39 (s, 3H), 3.93 (s, 3H), 7.35 (d, *J* = 8.4 Hz, 2H), 8.05 (d, *J* = 8.4 Hz, 2H).

^{13}C NMR (100 MHz, $CDCl_3$): δ 10.3, 18.5, 37.6, 52.4, 126.3, 127.8, 130.7, 149.4, 166.5, 172.0.

HRMS (ESI) m/z $[M+H]^+$ calcd for $C_{14}H_{16}NO_3$ 246.1125; Found 246.1127.

*Preparation of compound **12***

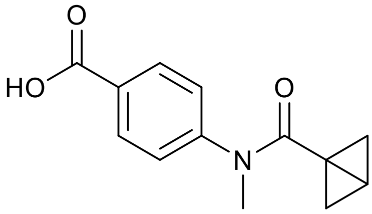

12

4-(*N*-methylbicyclo[1.1.0]butane-1-carboxamido)benzoic acid (12)

To the solution of **11** (44 mg, 0.18 mmol, 1 equiv.) in MeOH/ water (1:1, 10 mL) was added sodium carbonate (400 mg, 3.77 mmol, 21 equiv.). This mixture was stirred overnight at 50 ºC. The volatile components were removed *in vacuo*. The solution was cooled to 0 ºC and acidified to pH 4-5 with 1 M HCl. The cold aqueous layer was extracted five times with ethyl acetate. The combined organic layers were dried over Na_2SO_4, filtered and concentrated *in vacuo* to give an off-white solid of **12** (38 mg). Yield = 92%.

^{1}H NMR (400 MHz, CD_3OD): δ 0.86 (d, J = 2.4 Hz, 2H), 1.85 (d, J = 3.3 Hz, 2H), 2.15-2.18 (m, 1H), 3.38 (s, 3H), 7.45 (d, J = 8.6 Hz, 2H), 8.07 (d, J = 8.6 Hz, 2H).

^{13}C NMR (100 MHz, CD_3OD): δ 10.8, 19.0, 38.0, 38.1, 127.7, 130.3, 131.8, 150.1, 169.1, 174.2.

HRMS (ESI) m/z $[M+H]^+$ calcd for $C_{13}H_{14}NO_3$ 232.0968; Found 232.0965.

Compounds **13** and **14** were synthesized according to procedures described in literature.[2]

Preparation of compound ***2***

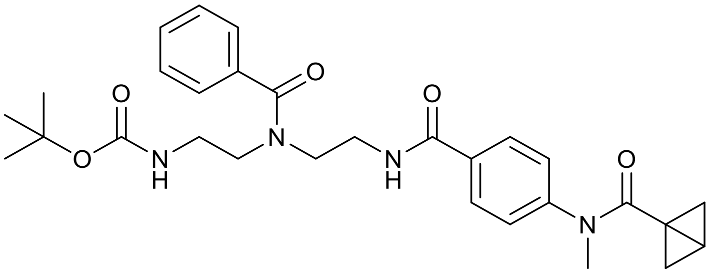

2

***tert*-Butyl (2-(N-(2-(4-(N-methylbicyclo[1.1.0]butane-1-carboxamido) benzamido)ethyl)benzamido)ethyl) carbamate (2)**

To a stirred solution of **12** (30 mg, 0.13 mmol, 1 equiv.) in dry DMF was added propanephosphonic acid anhydride (50 wt% in ethyl acetate, 90 µL, 0.14 mmol, 1.1 equiv.), *N,N*-diisopropylethylamine (70 µL, 0.40 mmol, 3.1 equiv.) and *tert*-butyl (2-(*N*-(2-aminoethyl)benzamido)ethyl)carbamate **14** (48 mg, 0.14 mmol, 1.2 equiv.). After stirring the reaction overnight under nitrogen at room temperature, the TLC analysis showed the complete exhaustion of the starting material. Saturated sodium bicarbonate was added to the reaction mixture and stirring was continued for 30 minutes. The aqueous layer was extracted five times with ethyl acetate. The combined organic layers were dried over Na_2SO_4, filtered and concentrated *in vacuo.* The residue was column chromatographed on silica gel (isocratic 4% methanol/dichloromethane) to give a white solid of **2** (29 mg). Yield = 43%.

^{1}H NMR (400 MHz, $CDCl_3$): δ 0.81 (s, 2H), 1.42 (s, 9H), 1.83 (s, 2H), 2.07 (s, 1H), 3.26-3.88 (m, 11H), 7.31-7.37 (m, 5H), 7.56 (s, 1H), 7.85-7.95 (m, 3H).

^{13}C NMR (100 MHz, $CDCl_3$): δ 10.2, 18.2, 28.5, 29.8, 37.5, 37.8, 39.0, 40.2, 44.9, 49.8, 53.6, 79.9, 126.4, 126.6, 127.2, 128.2, 128.6, 129.8, 131.7, 136.1, 148.1, 148.6, 164.9, 166.9, 171.9, 174.5.

HRMS (ESI) m/z $[M+H]^+$ calcd for $C_{29}H_{37}N_4O_5$ 521.2759; Found 521.2758.

*Preparation of compound **1a***

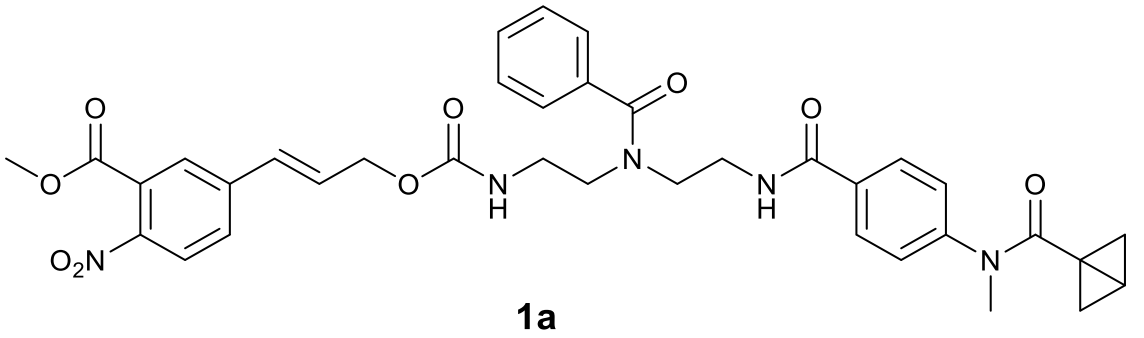

1a

Methyl (E)-5-(5-benzoyl-1-(4-(N-methylbicyclo[1.1.0]butane-1-carboxamido)phenyl)-1,9-dioxo-10-oxa-2,5,8-triazatridec-12-en-13-yl)-2-nitrobenzoate (1a)

To the stirred solution of **12** (25 mg, 0.11 mmol, 1 equiv.) in dry DMF (5 mL) was added propanephosphonic acid anhydride (50 wt% in ethyl acetate, 110 µL, 0.16 mmol, 1.5 equiv.), *N,N*- diisopropylethylamine (57 µL, 0.32 mmol, 3.0 equiv.) and **13** (56 mg, 0.12 mmol, 1.1 equiv.). This reaction mixture was sealed, flushed with nitrogen and stirred for 21 h at room temperature. Saturated sodium bicarbonate was added and stirring was continued for 30 minutes. The aqueous layer was extracted four times with ethyl acetate. The combined organic l ayers were dried over Na_2SO_4, filtered and concentrated *in vacuo.* The residue was column chromatographed on silica gel (gradient 1 % to 4 % methanol/dichloromethane) to give a white solid of **1a** (29 mg). Yield = 23%.

^{1}H NMR (400 MHz, $CDCl_3$): δ 0.79 (s, 2H), 1.83 (s, 2H), 2.06 (s, 1H), 3.35-3.92 (m, 14H), 4.62-4.71 (m, 2H), 6.42-6.46 (m, 1H), 6.65 (d, *J* = 15.6 Hz, 1H), 7.07-7.92 (m, 12H).

^{13}C NMR (100 MHz, $CDCl_3$): δ 10.2, 18.2, 29.8, 37.6, 37.7, 39.7, 40.1, 45.1, 49.9, 53.5, 64.8, 124.4, 124.8, 126.4, 126.6, 127.5, 128.1, 128.7, 128.8, 129.2, 129.8, 131.5, 136.0, 141.7, 146.7, 148.2, 156.1, 166.2, 167.2, 171.9, 174.4.

HR-MS (ESI) m/z $[M+H]^+$ calcd for $C_{36}H_{38}N_5O_9$ 684.2665; Found 684.2655.

*Preparation of compound **1***

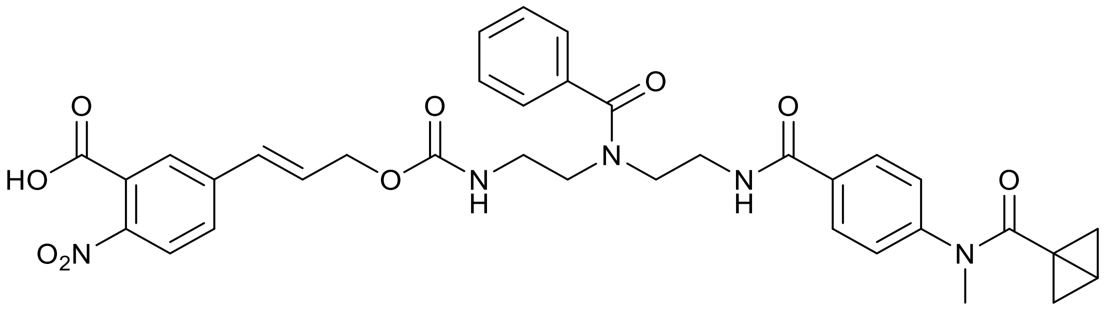

1

(E)-5-(5-benzoyl-1-(4-(N-methylbicyclo[1.1.0]butane-1-carboxamido)phenyl)-1,9-dioxo-10-oxa-2,5,8-triazatridec-12-en-13-yl)-2-nitrobenzoic acid (1)

To the solution of **1a** (6 mg, 8.78 μmol) in methanol/ water (1:1, 2 mL) was added lithium hydroxide (150 uL, 2M solution in water). This was stirred for 30 minutes at room temperature. After the complete consumption of the starting material was confirmed by the TLC analysis, the volatiles were removed *in vacuo.* The reaction solution was cooled to 0 ºC and acidified to pH 4-5 with 1 M HCl. The cold aqueous layer was extracted three times with ethyl acetate. The combined organic layers were dried over Na_2SO_4, filtered and concentrated *in vacuo* to give an off-white solid of **1** (5 mg, yield = 86 %). This solid was treated with the amine-derivatized magnetic beads without further purification.

^{1}H NMR (400 MHz, CD_3OD): δ 0.82 (d, J = 21.6 Hz, 2H), 1.83 (d, J = 15.6 Hz, 2H), 2.14-2.17 (m, 1H), 3.24- 3.84 (m, 11H), 4.67-4.79 (m, 2H), 6.54-6.61 (m, 1H), 6.71-6.78 (m, 1H), 7.26-7.44 (m, 7H), 7.57-7.89 (m, 5H).

HR-MS (ESI) m/z $[M+H]^+$ calcd for $C_{35}H_{36}N_5O_9$ 670.2508; Found 670.2503.

Synthesis of reduced lipoic acid

The method was adapted from procedure reported in literature.[3]

To a solution of (±)-α-lipoic acid (1.0 g) in 0.25 *N* sodium bicarbonate (19.5 mL), a total of 0.2 g of sodium borohydride was added portion wise. The mixture was well stirred and kept below 5 ºC. After 30 minutes of stirring, toluene (16 mL) was added, and the colorless reaction was acidified to pH 1 with ice cold 5 *N* HCl. The content of the toluene layer was concentrated *in vacuo* to yield 0.7 g of reduced lipoic acid: lipoic acid in the ratio of 1:0.23.

^1H NMR (400 MHz, $CDCl_3$): δ 1.29-1.37 (m, 1H), 1.43-1.79 (m, 7H), 1.87-1.95 (m, 1H), 2.36-2.40 (m, 2H), 2.43-2.50 (m, 0.25H), 2.62-2.82 (m, 2H), 2.86-2.97 (m, 1H), 3.08-3.22 (m, 0.45H), 3.54-3.61 (m, 0.23H).

*Preparation of 1-butanethiol-probe conjugate **TC11** for the LOD determination*

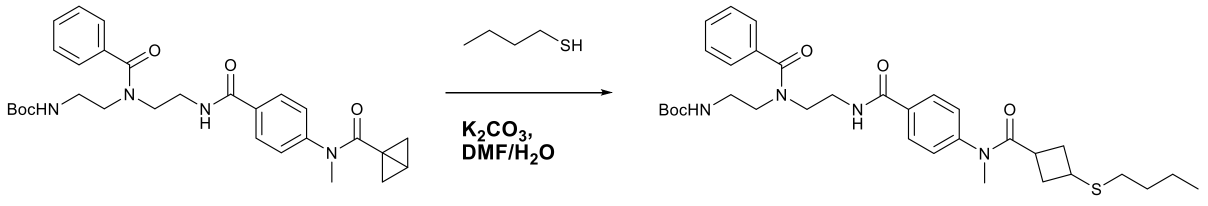

2 **TCP_{11}**

To compound **2** (12 mg, 23 µmol) in DMF (1 mL) was added aqueous potassium carbonate (1 mL, 129 mM). 1-Butanethiol (20 µL, 173 µmol) was added and the reaction was stirred overnight at room temperature. Aqueous sodium bicarbonate solution was added and the mixture was stirred under nitrogen for 1 h. The aqueous layer was extracted four times with ethyl acetate, and the combined organic layers were dried over Na_2SO_4, filtered, and concentrated *in vacuo* to give a yellow oil. This was column chromatographed on silica gel (isocratic 6% methanol/dichloromethane) to give an off-white solid of **TCP11** (7 mg). Yield = 50%.

^1H NMR (400 MHz, $(CD_3)_2SO$): δ 0.83 (t, 3H, J = 7.2 Hz), 1.23-1.44 (m, 13H), 1.98-2.11 (m, 3H), 2.36-2.42 (m, 2H), 2.99-3.04 (m, 2H), 3.16-3.63 (m, 12H), 7.30-7.38 (m, 7H), 7.79-7.89 (m, 2H).

^1H NMR (400 MHz, $CDCl_3$): δ 0.88 (t, 3H, J = 7.2 Hz), 1.42 (s, 9H), 1.50- 3.88 (m, 24H), 7.16-7.90 (m, 9H).

^{13}C NMR (100 MHz, $CDCl_3$): δ 13.8, 22.2, 28.5, 29.9, 30.5, 31.3, 32.0, 32.4, 33.2, 34.5, 34.6, 35.1, 35.7, 37.5, 40.6, 50.0, 50.1, 126.5, 127.2, 127.4, 128.9, 129.9, 135.9, 136.0, 152.6, 173.2.

HRMS (ESI) m/z $[M+H]^+$ calcd for $C_{33}H_{47}N_4O_5S$ 611.3262; Found 611.3255.

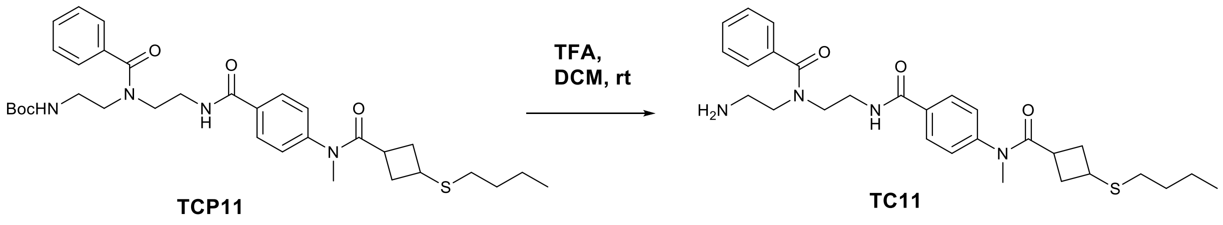

To compound **TCP11** (2.5 mg, 4 μmol) in DCM (2 mL) was added trifluoroacetic acid (100 μL) and stirred overnight at room temperature. After the completion of reaction was confirmed by TLC, the volatiles were removed *in vacuo*. The resulting residue was dissolved in methanol to obtain a solution of 20 mM. This solution was further diluted to different concentrations with 5 % acetonitrile/ water and the LODs of the solutions were measured.

Preparation of Boc-protected cysteine persulfide conjugate ***TCP21*** *for the LC-MS analysis*

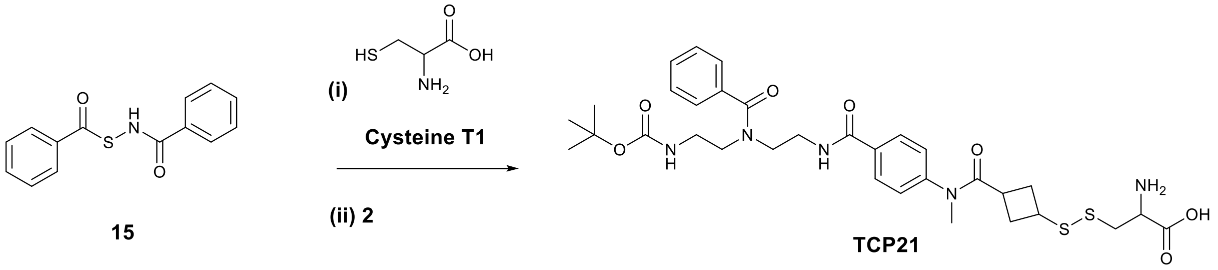

Compound **15** was synthesized according to procedure reported in literature.[4]

^{1}H NMR (400 MHz, $CDCl_3$) of **15**: δ 7.49-7.53 (m, 4H), 7.55-7.61 (m, 1H), 7.63-7.67 (m, 1H), 7.93 (d, J = 7.6 Hz, 1H).

To compound **15** (11.1 μl, 80 mM in THF) was added L-cysteine **T1** (200 μl, 400 mM in PBS) and stirred at room temperature for 2 minutes. Probe **2** (200 μL, 4.8 mM in THF) was added dropwise over 10 minutes. The reaction mixture was analyzed using UHPLC-MS at time-points 5 min, 10 min, 15 min, 20 min and 1 h.

5. Methods

5.1 Preparation of immobilized probe A

Magnabind amine derivatized beads (50 µL, Thermo Scientific™) solution was taken in a 1.5 mL Eppendorf tube. Original solution from the supplier was removed using magnetic separation. The beads were washed with THF (2 x 200 µL) followed by DMF (1 x 200 µL). To the washed beads, DMF (200 µL) and DIPEA (5 µL) were added, and vortexed for 30 s. This solution was removed using the magnetic separation. The beads were washed with DMF (1 x 200 µL). To the washed beads, an amide coupling solution (13.5 mM PyBOP, 13.3 mM HOBt, 100 µL DMF), DIPEA (5 µL) and compound **1** (8.9 mM, 100 µL DMF) were added. This mixture was incubated using a Thermomixer (1,500 rpm, 30 ºC, 16 h). The solution was removed using the magnetic separation and the beads were washed with DMF (2 x 200 µL). The beads were suspended in DMF (100 µL) and utilized for the biological sample treatment.

5.2 Preparation of LC-MS standard of TC21

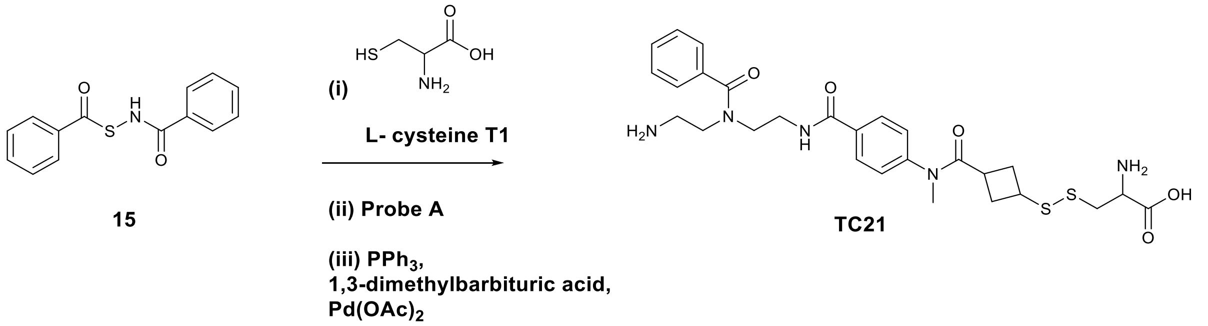

To compound **15** (11.1 µl, 80 mM in THF) was added L- cysteine **T1** (200 µl, 400 mM in PBS) and stirred at room temperature for 2 minutes. Magnetic bead-bound probe **A** (50 µL) was added to the reaction mixture and stirred at 30 ºC. At the reaction intervals of 10 min and 60 min, 25 µL of the reaction mixture was transferred to a glass vial and incubated with triphenylphosphine (48.5 µL, 12.9 mM in THF), 1,3-dimethylbarbituric acid (45.0 µL, 30.7 mM in THF), and $Pd(OAc)_2$ solution (42.0 µL, 6.53 mM in THF) at 25 ºC for 16 h. The supernatants were separated from the beads using magnetic separation. The supernatants were concentrated *in vacuo*. The residues were redissolved in LCMS-grade methanol (15 µL) and triphenylphosphine and triphenylphosphine oxide were precipitated through the addition of water (60 µL). The suspension was centrifuged in a benchtop centrifuge (12,000 g, 5 min). The supernatants were transferred into Eppendorf tubes and concentrated *in vacuo*. The residues were dissolved in 25 µL of LCMS-grade methanol, diluted to 1:3 with 5% v/v acetonitrile/water and analyzed by UHPLC-MS.

5.3 Preparation of fecal metabolite extracts

A scalpel was used to collect approximately 60 mg of the frozen fecal sample from eight different patients (stored at - 80 ºC) in specialized tube D (MP Biomedicals). Ultrapure water (100 μL) and LCMS-grade methanol (400 μL) were added to each tube. The mixtures were vortexed and homogenized by a FastPrep 24 homogenizer (3 cycles, 6 m/s, 40 s, MP Biomedicals). The mixtures were transferred from tube D to Eppendorf tubes and stored at -20 ºC for 1 h. The mixtures were centrifuged (17,500 g, 5 min, 4 ºC). The supernatants were collected, combined and the solvent was removed *in vacuo*. The dried extract was dissolved in DMF (380 μL) and treated with the immobilized probe **A**.

5.4 Preparation of plasma metabolite extracts

Ice cold methanol (200 μL) was added to plasma sample aliquot (50 μL) for protein precipitation. The sample was vigorously shaken for 30 s and cooled at 4 ºC for 30 min. The supernatant was transferred into an Eppendorf tube and methanol was removed *in vacuo*. The dried extract was dissolved in DMF (200 μL) and treated with the immobilized probe **A**.

5.5 Treatment of fecal metabolite extract

A suspension of immobilized probe **A** in DMF (100 μL) was added to the fecal extract solution (380 μL DMF). Triethylamine (60 μL) was added and the mixture was stirred at 30 ºC for 22 h. The fecal extract solution was removed and the beads were washed with THF (2 x 200 μL) and resuspended in THF (300 μL THF). For the control experiment, the amine-derivatized beads (unmodified) were incubated with the fecal extract solution under the same reaction conditions.

5.6 Treatment of plasma metabolite extract

A suspension of immobilized probe **A** in DMF (100 μL) was added to the plasma extract solution (200 μL DMF). Triethylamine (60 μL) was added and the mixture was stirred at 30 ºC for 22 h. The plasma extract solution was removed and the beads were washed with THF (2 x 200 μL) and resuspended in THF (300 μL THF). For the control experiment, the amine-derivatized beads (unmodified) were incubated with the plasma extract solution under the same reaction conditions.

5.7 Preparation of bacterial strain lysates and supernatants

Salmonella enterica serovar Typhimurium SL1344 (*Salmonella*)**,** *Shigella flexneri* M90T (*Shigella*), and *Escherichia coli* DH10B (*E. coli*) were used as bacterial strains in this study. Each bacterium was inoculated in 10 mL Luria broth (LB) and grown for 16 h overnight at 37 ^{o}C. The bacteria in each overnight culture were pelleted by centrifugation (4 ^{o}C, 15 min, 20.000g). For each sample, 2 mL of the supernatant was diluted to 10 mL with mass spectrometry grade methanol. The remaining supernatant was removed, and 4 ml of ethanol:water (3:2) solution was added to the bacterial pellet. The pellet lysate mixtures were heated at 78 ^{o}C for 3 min with rigorous vortexing every minute, cooled, and subsequently cleared by centrifugation (4 ^{o}C, 15 min, 20,000g). The supernatant and pellet lysate samples were dried *in vacuo*. The dried samples were dissolved in dimethylformamide and treated with probe **A**.

In order to induce oxidative stress, *E. coli* DH10b was inoculated in 20 mL of Luria broth (LB) and grown for 16 h overnight at 37 ^{o}C. Subsequently, half of the culture was treated with hydrogen peroxide (H_2O_2) at a final concentration of 1 mM for 30 min at 37 ^{o}C, while the other half was left untreated and used as a control. Bacterial pellets were lysed and collected as specified above.

5.8 Cleavage of the bead-bound probe A

The THF suspension of the treatment and control beads were transferred to glass vials. Triphenylphosphine (97.0 µL, 12.9 mM in THF, 1.25 µmol) and 1,3-dimethylbarbituric acid (90.0 µL, 30.7 mM in THF, 2.76 µmol) solutions were added to the vial, followed by palladium (II) acetate solution (84.0 µL, 6.53 mM in THF, 549 nmol). The vials were sealed and a stream of nitrogen was passed through until approximately half the volume of the suspension remained. The vials were shaken using Thermomixer (700 rpm) at 25 ^{o}C for 16 h. The supernatants were separated from the beads using magnetic separation. The supernatants were concentrated *in vacuo*. The residues were redissolved in LCMS-grade methanol (30 µL) and triphenylphosphine and triphenylphosphine oxide were precipitated through the addition of water (120 µL). The suspension was centrifuged in a benchtop centrifuge (12,000 g, 5 min). The supernatants were transferred into Eppendorf tubes and concentrated *in vacuo*. The residues were dissolved in 100 µL of LCMS-grade methanol, diluted to 1:1 with 5% v/v acetonitrile/water and analyzed by UHPLC-MS.

5.9 Preparation of probe-conjugated thio-intermediates TCP1-TCP20

To the simplified probe **2** (0.2 mg, 1 equiv.) in DMF (200 μL) was added the thiol **T1-T20** (10 equiv.) either dissolved in water or DMF (100 μL). Potassium carbonate (20 equiv.) in water (100 μL) or trimethylamine (80 equiv.) was added. The reaction vials were sealed under nitrogen and stirred overnight at room temperature or 28 ^{o}C. An aliquot (5 μL) was diluted to 100 μL with 5% v/v acetonitrile/ water and analyzed by UHPLC-MS.

5.10 Preparation of probe-conjugated standards TC1-TC20

After confirmation of the successful conjugation under **5.9**, trifluoroacetic acid (100 μL) was added to each reaction mixture (where conjugated using K_2CO_3) and stirred overnight at room temperature. The reaction mixture was concentrated *in vacuo*. An aliquot (5 μL) of the reaction mixture was diluted to 50 μL with LCMS-grade methanol. An aliquot (10 μL) of this solution was further diluted to 60 μL with 5% v/v acetonitrile/ water and analyzed by UHPLC-MS. This sequence of dilution was important for obtaining a clear sample solution.

5.11 Stability of thiol conjugates under Pd(0) conditions

The thiol conjugates **TC1- TC3**, **TC5**, **TC7**, **TC10**, **TC13** and **TC19** prepared above were incubated with Pd(0) reagents according to the method described in **5.8** and analyzed by UHPLC-MS.

5.12 Chemoselectivity test

Compound **2** (0.2 mg, 1 equiv.) or iodoacetamide (0.2 mg, 1 equiv.) was incubated with *N*-acetyl-L-cysteine **T2** (0.6 mg, 10 equiv.), L-serine (0.4 mg, 10 equiv.), L- lysine (0.6 mg, 10 equiv.) and potassium carbonate (0.5 mg, 10 equiv.) in 50 % DMF/H_2O (0.8 mL) at 28 ^{o}C for 22 h. The reaction was analyzed using UHPLC-MS for the formed conjugates.

5.13 LOD experiment

Synthetic conjugated thiol **TC11** was prepared in a solution of water and acetonitrile (95:5 v/v) at a range of concentrations (5 μM, 500 nM, 50 nM, 5 nM, 0.5 nM) before being submitted for UHPLC-MS analysis. The measurement was based on the conventional concepts that signal to noise ratios are at least higher than 3 corresponding to Limit of Detection (LOD). The signal to noise ratios were calculated according to European Pharmacopoeia guidelines by using MassLynx 4.1.

6. Ethical approval

Patient fecal samples were obtained in accordance with the World Medical Association Declaration of Helsinki and all patients gave written informed consent. Approval for the study was obtained from the ethical committee at Karolinska Institutet Hospital (Ethical approval number: Dnr 2017/290-31). Fecal samples were collected using routine clinical collection protocols and all patient codes have been removed in this publication. All samples were stored at -80 °C. Metadata of the patients can be found in Table S7.

7. References

(1) Tokunaga, K.; Sato, M.; Kuwata, K.; Miura, C.; Fuchida, H.; Matsunaga, N.; Koyanagi, S.; Ohdo, S.; Shindo, N.; Ojida, A. Bicyclobutane Carboxylic Amide as a Cysteine-Directed Strained Electrophile for Selective Targeting of Proteins. *J. Am. Chem. Soc.* **2020**, *142* (43), 18522-18531. DOI: 10.1021/jacs.0c07490.

(2) Lin, W.; Conway, L. P.; Vujasinovic, M.; Löhr, J. M.; Globisch, D. Chemoselective and Highly Sensitive Quantification of Gut Microbiome and Human Metabolites. *Angew. Chem. Int. Ed.* **2021**, *60* (43), 23232–23240. DOI: DOI: 10.1002/anie.202107101.

(3) Gunsalus, I. C.; Barton, L. S.; Gruber, W. Biosynthesis and Structure of Lipoic Acid Derivatives. *J. Am. Chem. Soc.* **1956**, *78* (8), 1763–1766. DOI: 10.1021/ja01589a079.

(4) Zhao, Y.; Wang, H.; Xian, M. Cysteine-Activated Hydrogen Sulfide (H2S) Donors. *J. Am. Chem. Soc.* **2011**, *133* (1), 15-17. DOI: 10.1021/ja1085723.

8. NMR spectra

^{1}H NMR (400 MHz, $CDCl_3$) of reduced lipoic acid

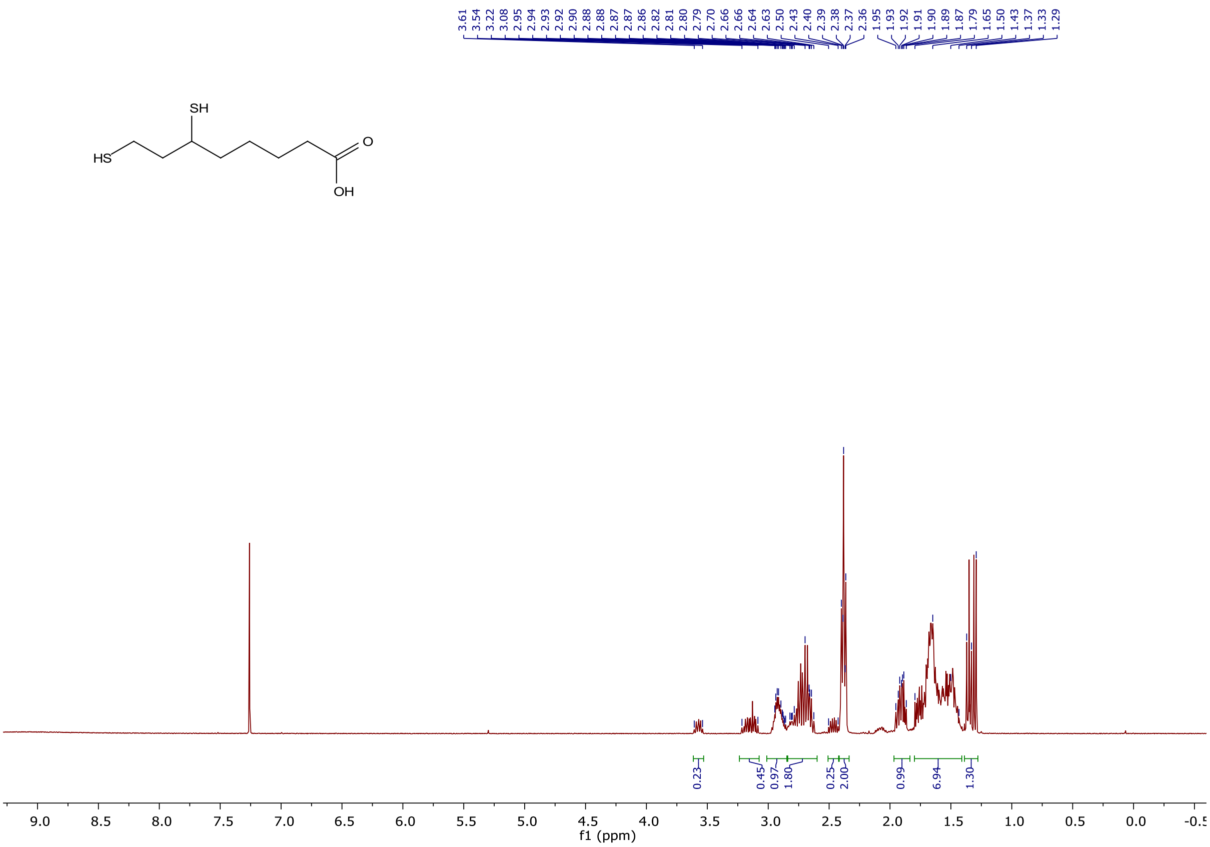

^{1}H NMR (400 MHz, $(CD_3)_2SO$) of **TCP11**

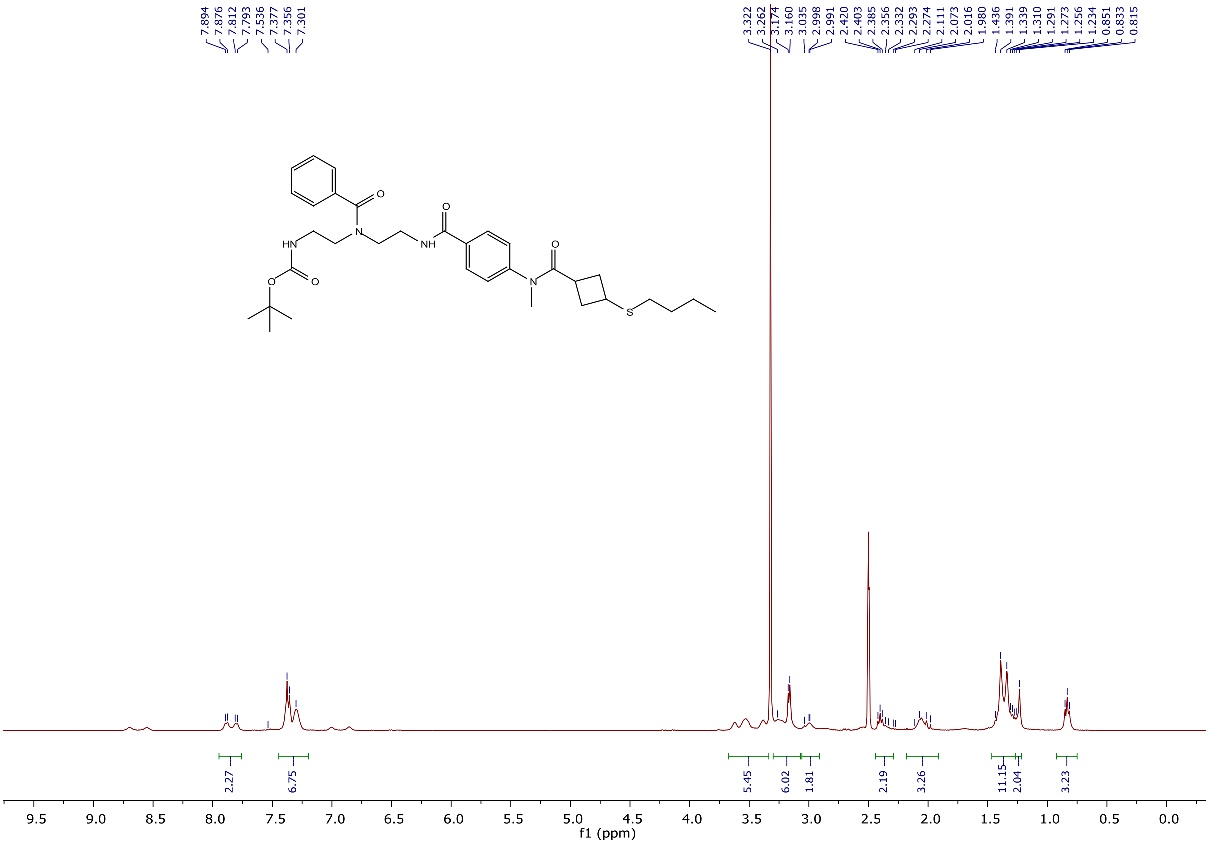

^{1}H NMR (400 MHz, $CDCl_3$) of **TCP11**

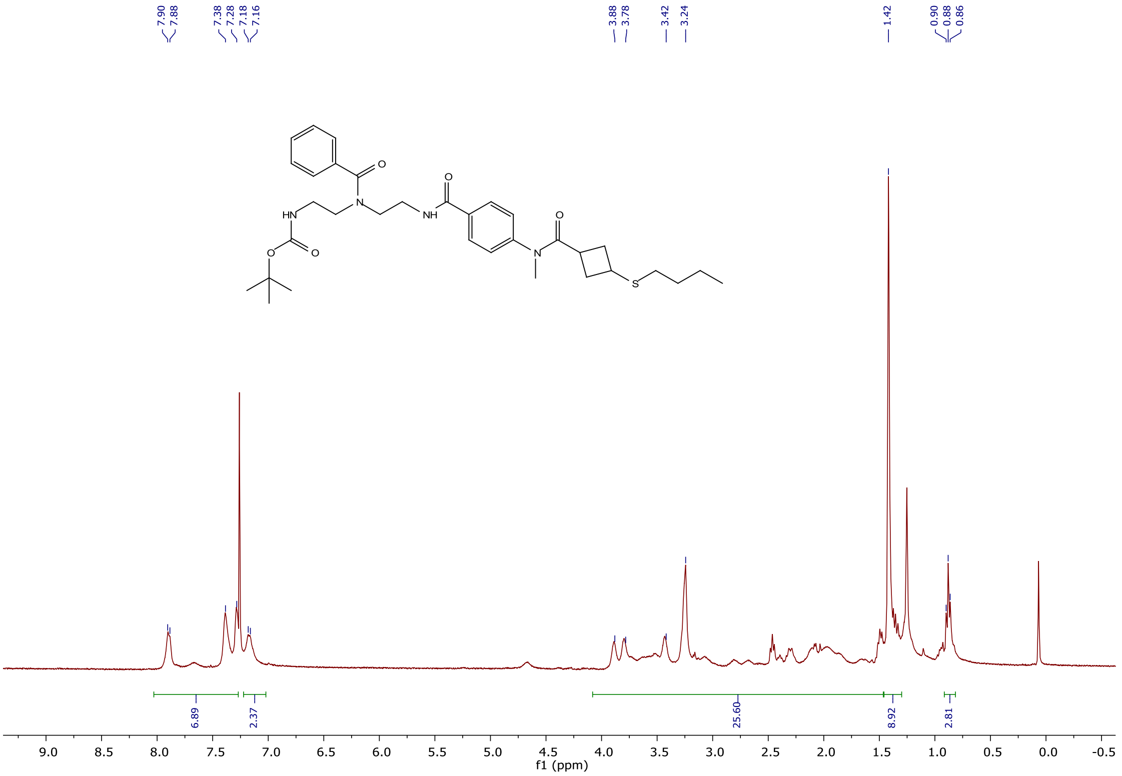

^{13}C NMR (100 MHz, $CDCl_3$) of **TCP11**

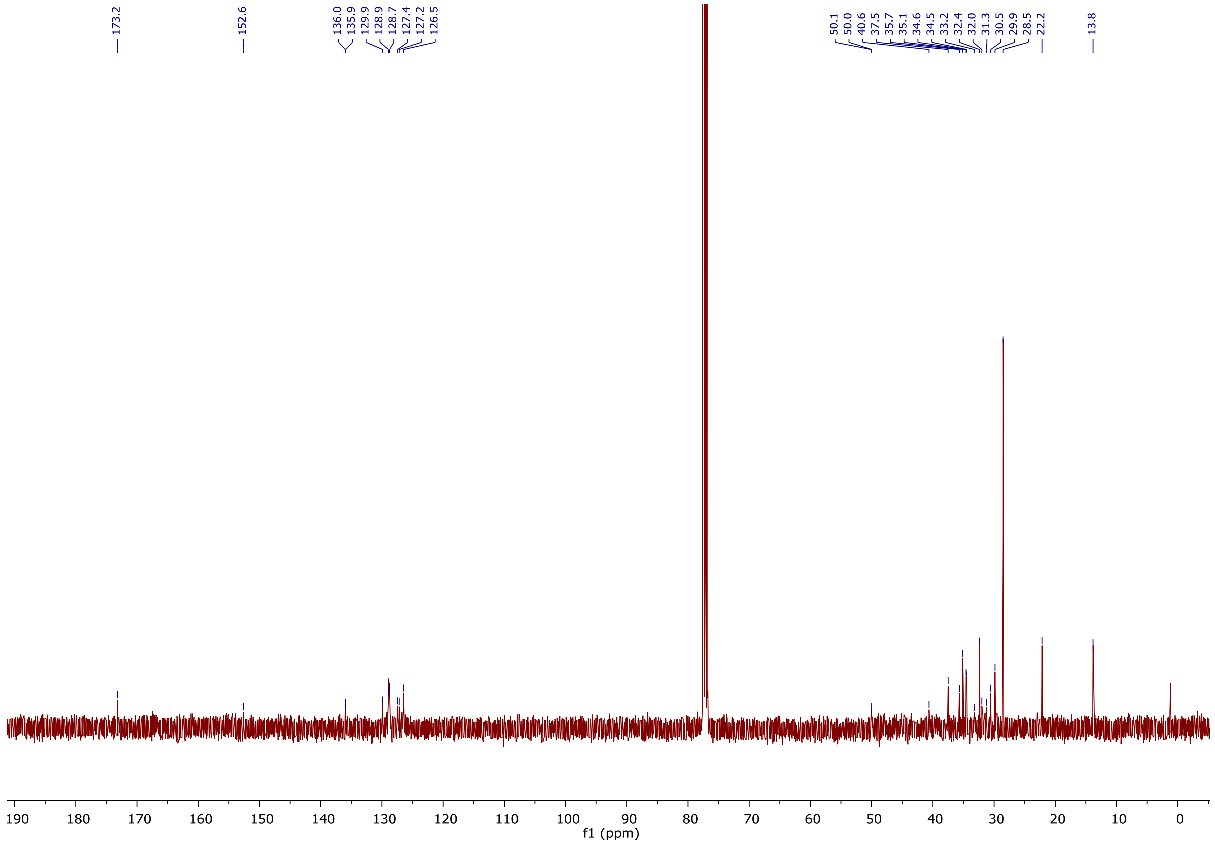

^{1}H NMR (400 MHz, $CDCl_3$) of **15**

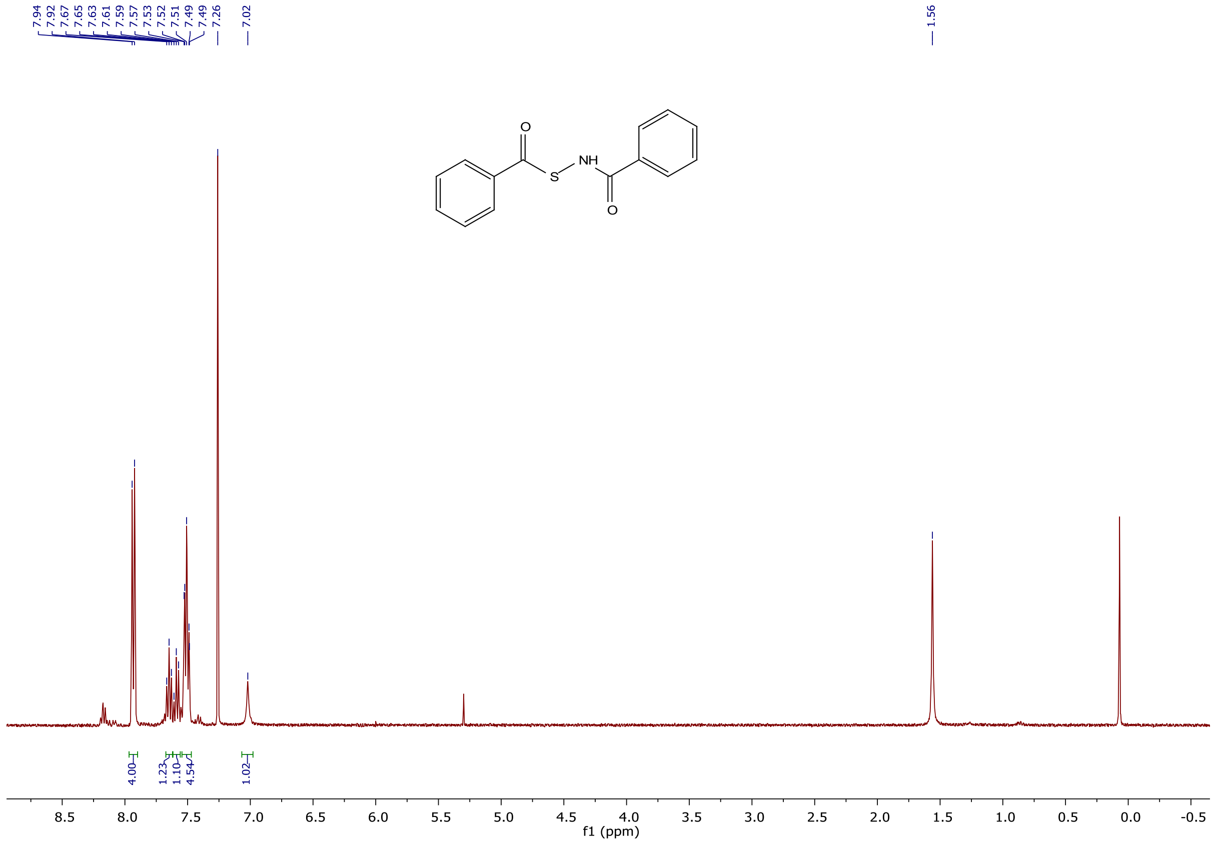

^{1}H NMR (400 MHz, $CDCl_3$) of **5**

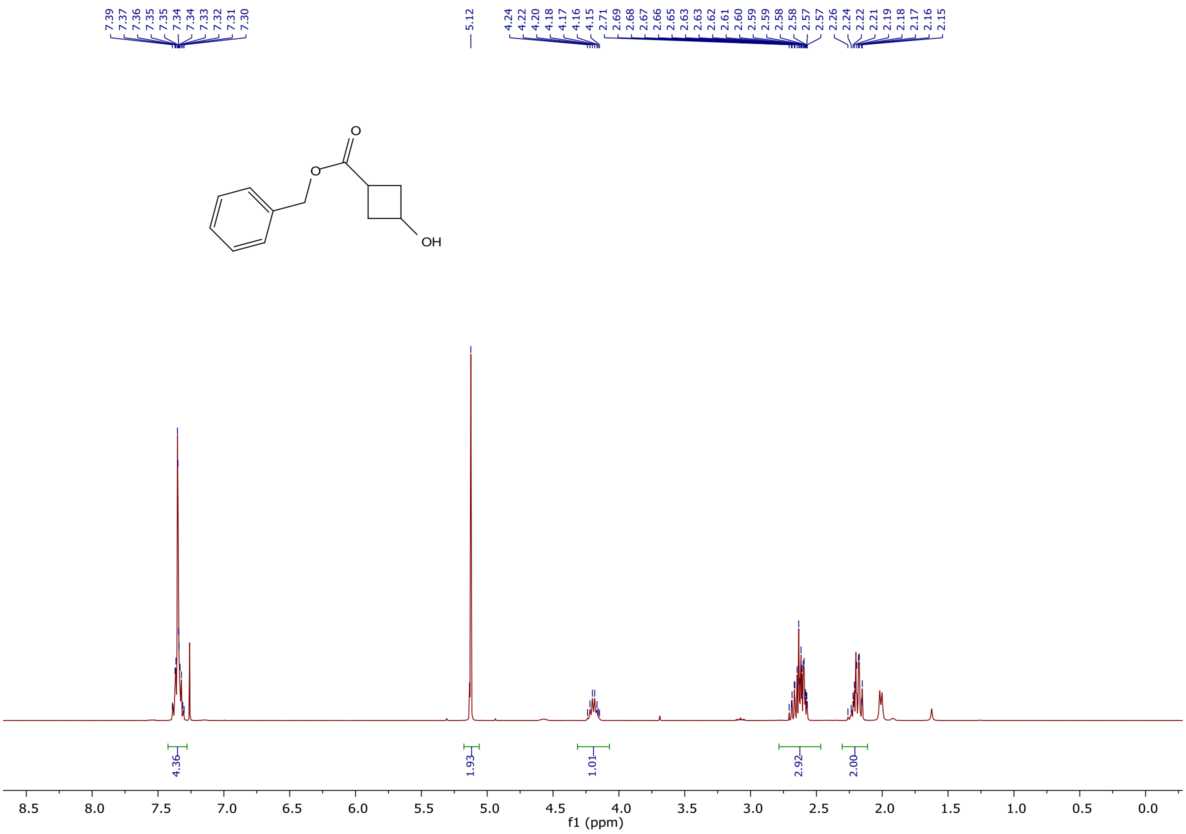

^{1}H NMR (400 MHz, $CDCl_3$) of **6**

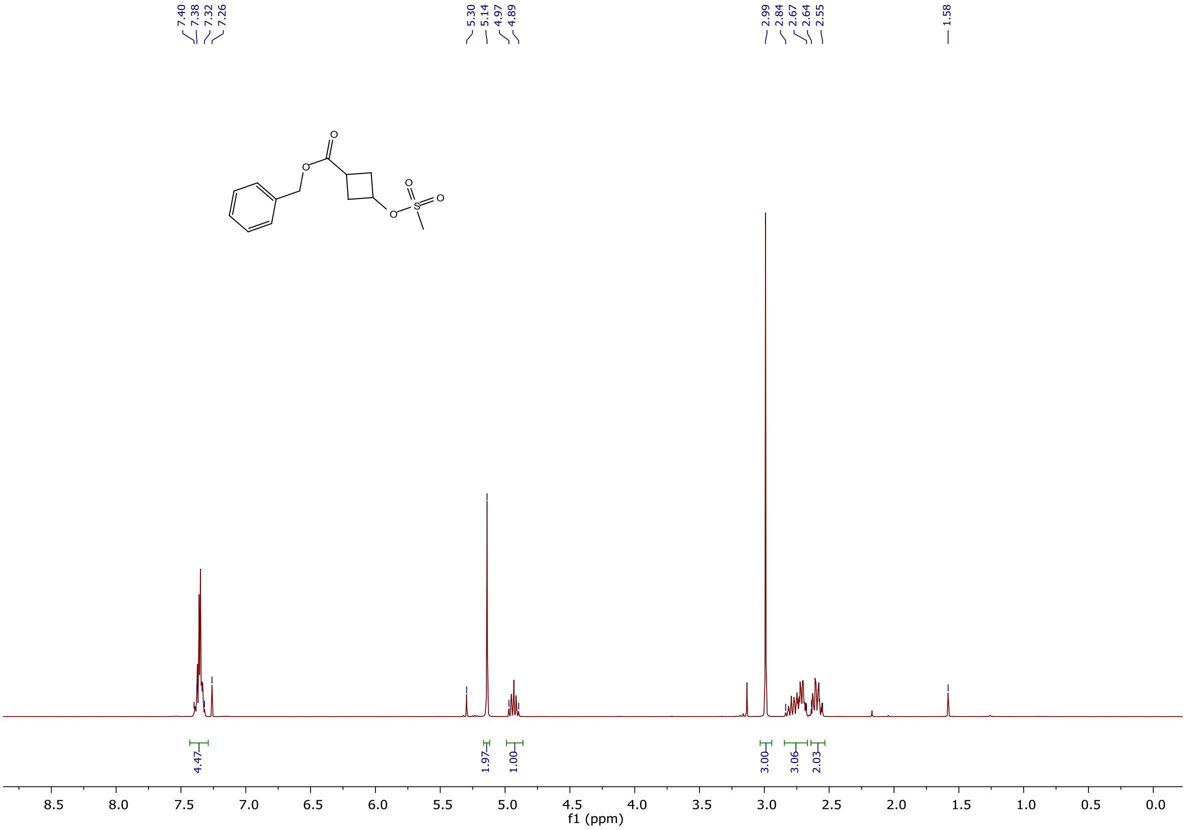

^{1}H NMR (400 MHz, $CDCl_3$) of the diastereomer mixture of **7**

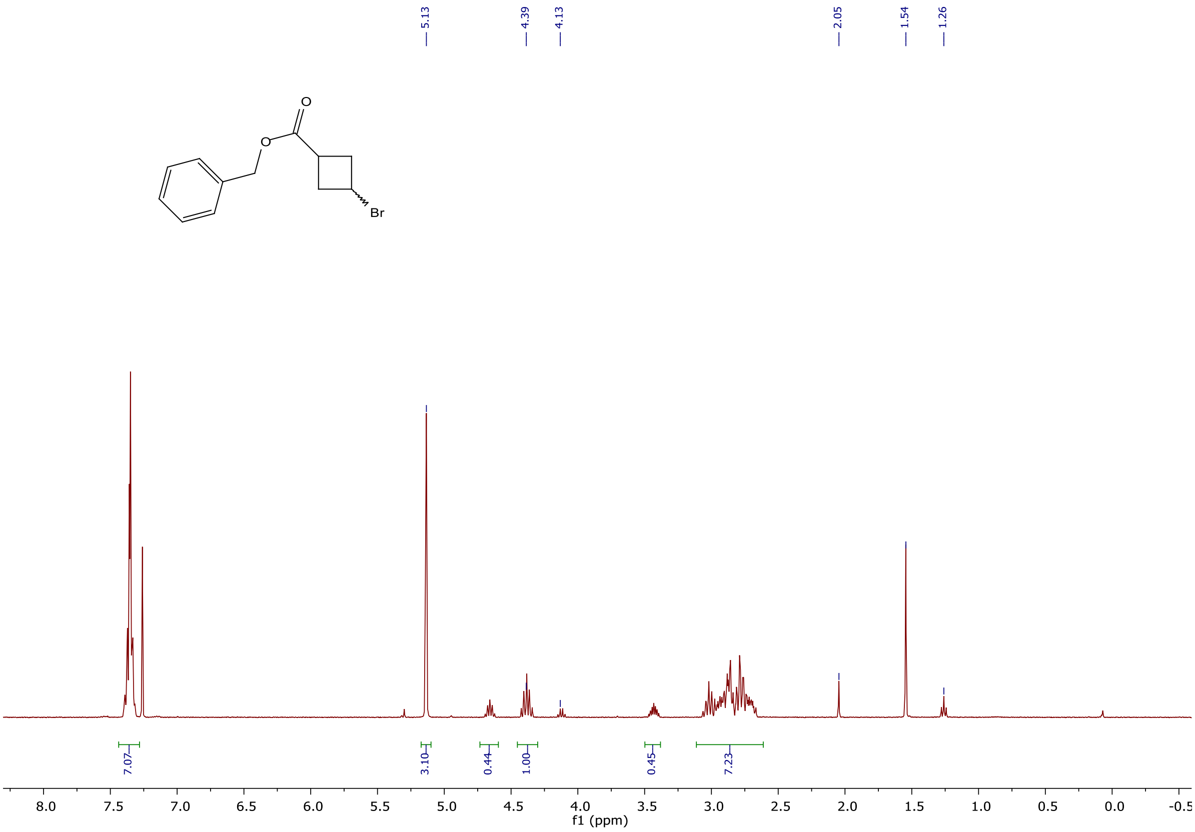

^{1}H NMR (400 MHz, $CDCl_3$) of major diastereomer of **7**

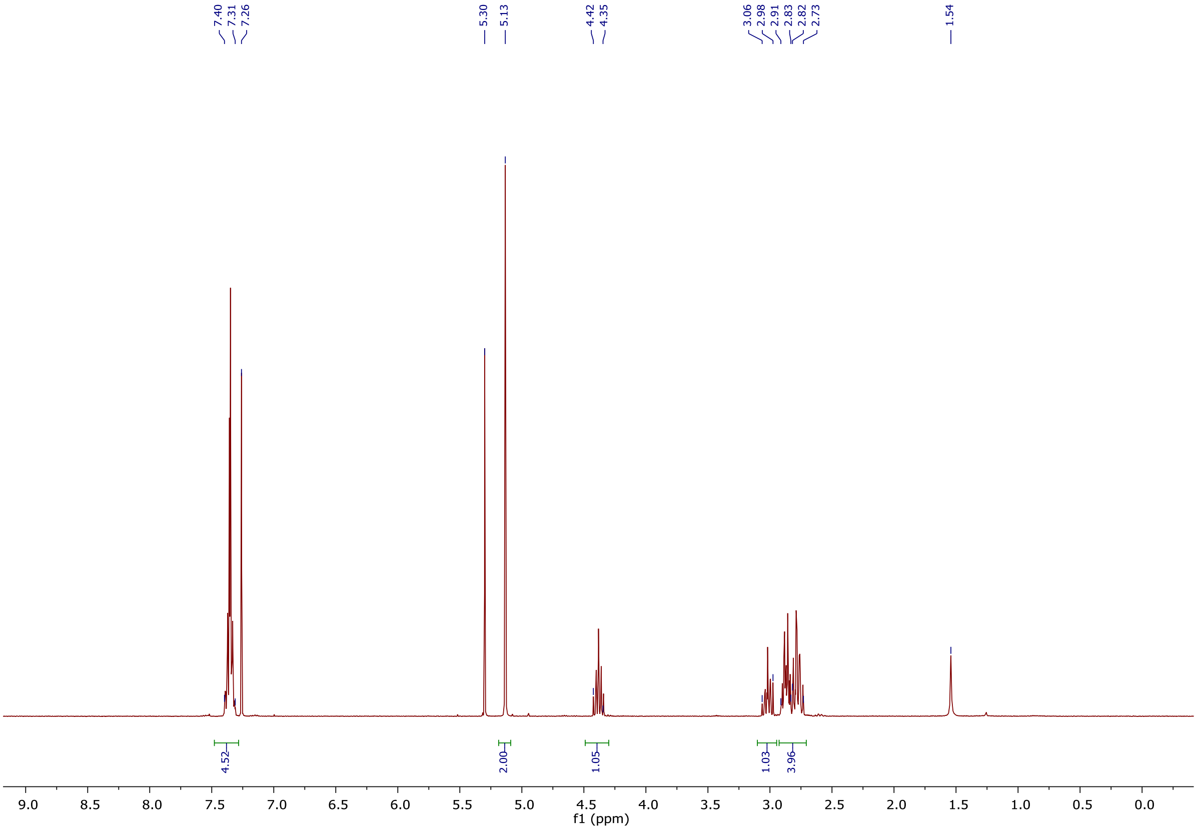

^{1}H NMR (400 MHz, $CDCl_3$) of minor diastereomer of **7**

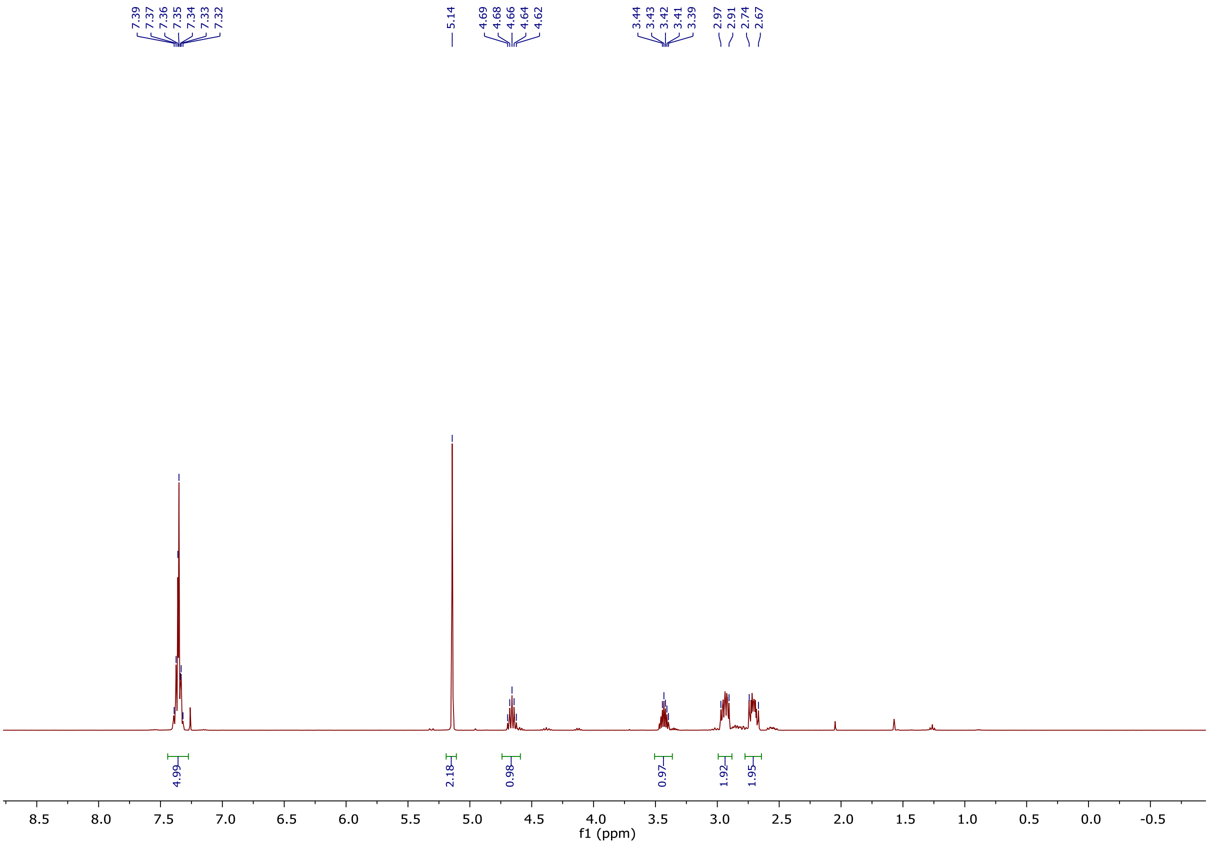

^{1}H NMR (100 MHz, CD_3OD) of **8**

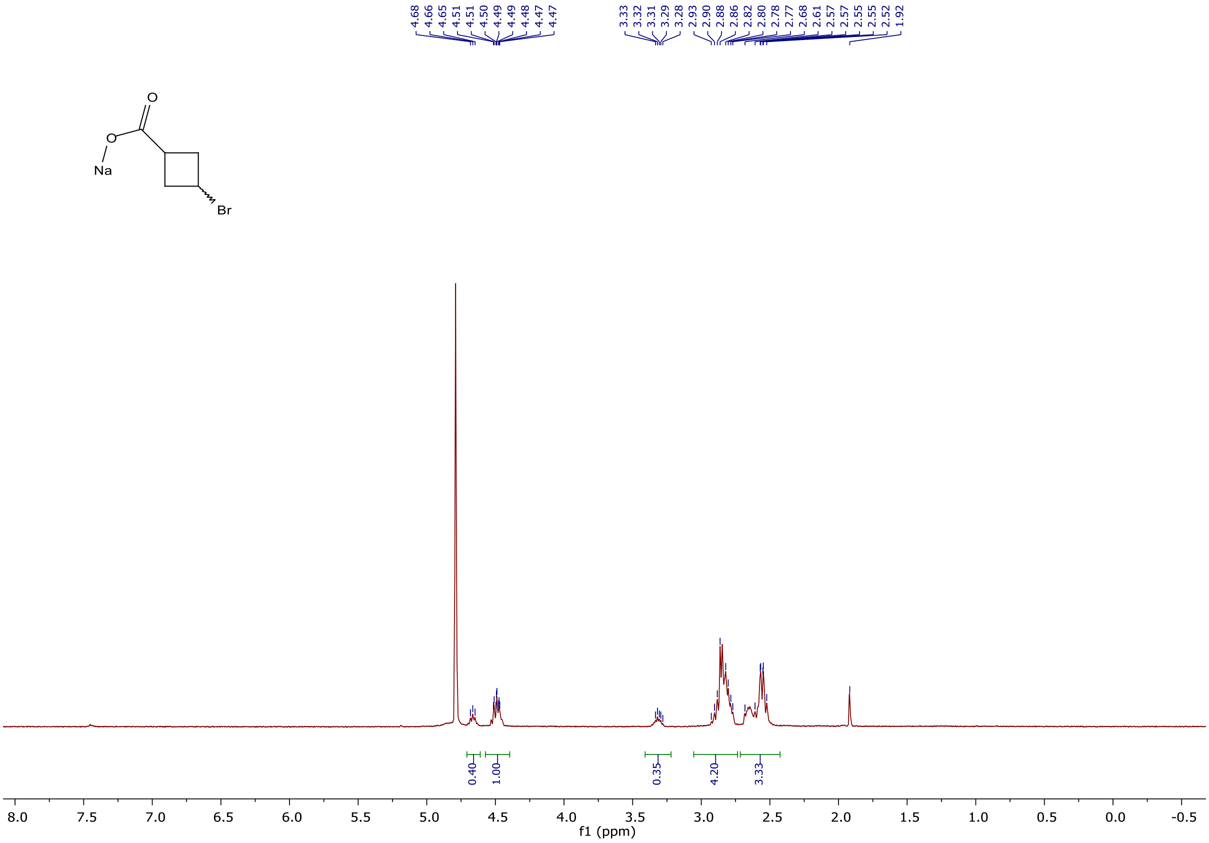

^{1}H NMR (400 MHz, $CDCl_3$) of **10**

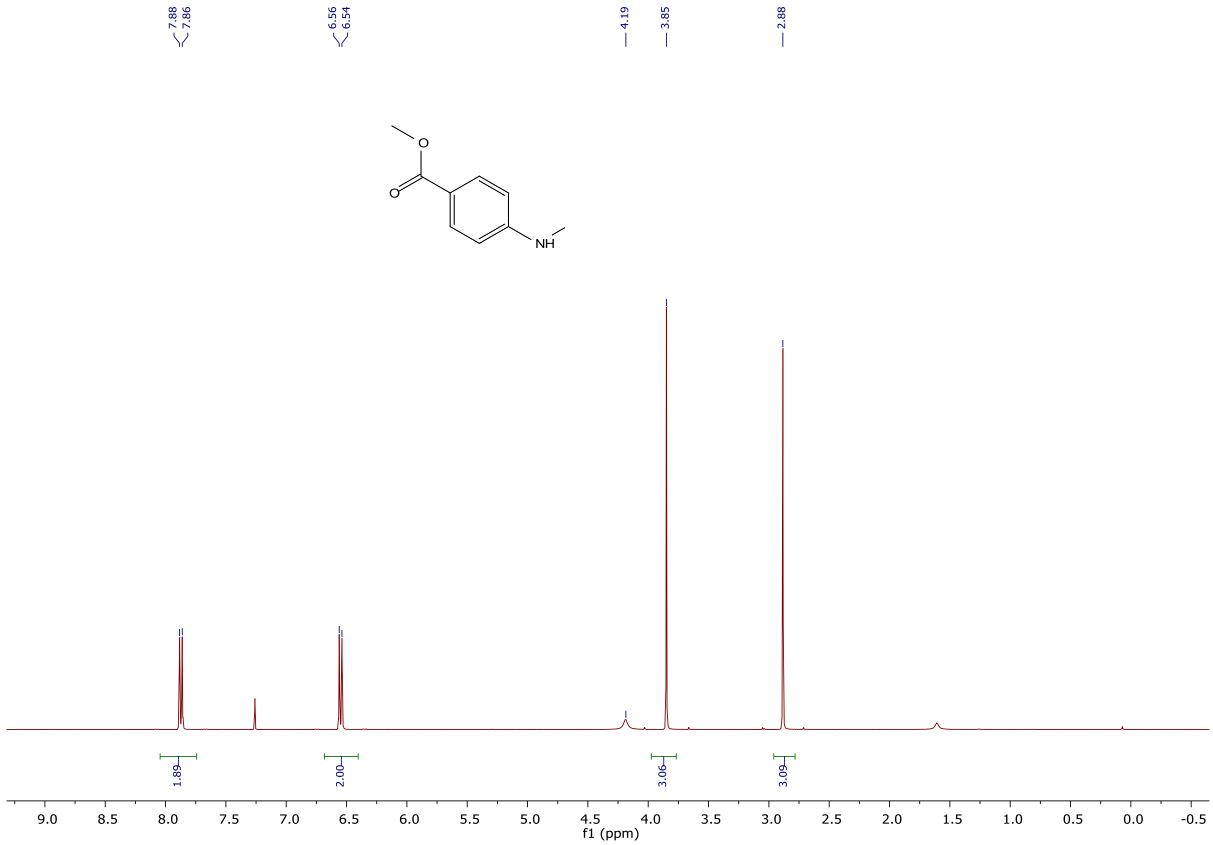

^{1}H NMR (400 MHz, $CDCl_3$) of **9**

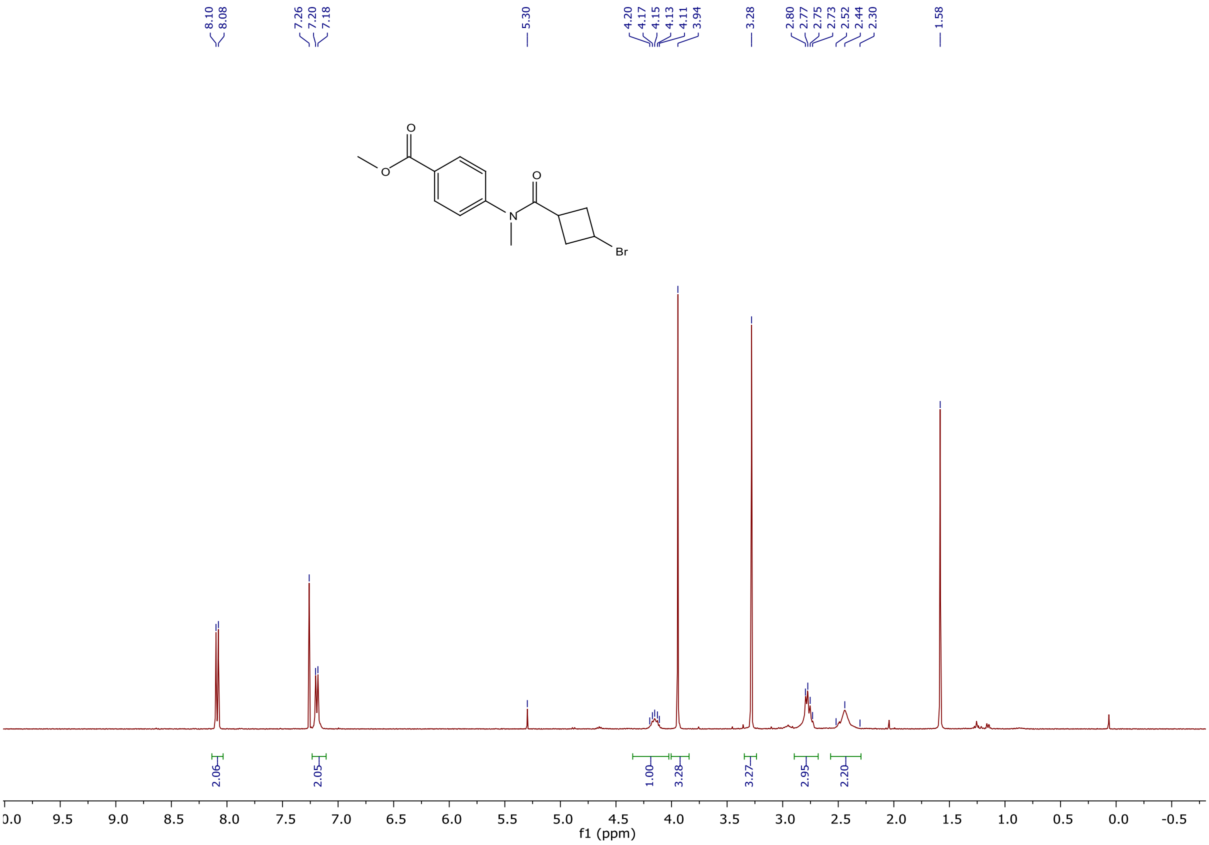

^{13}C NMR (100 MHz, $CDCl_3$) of **9**

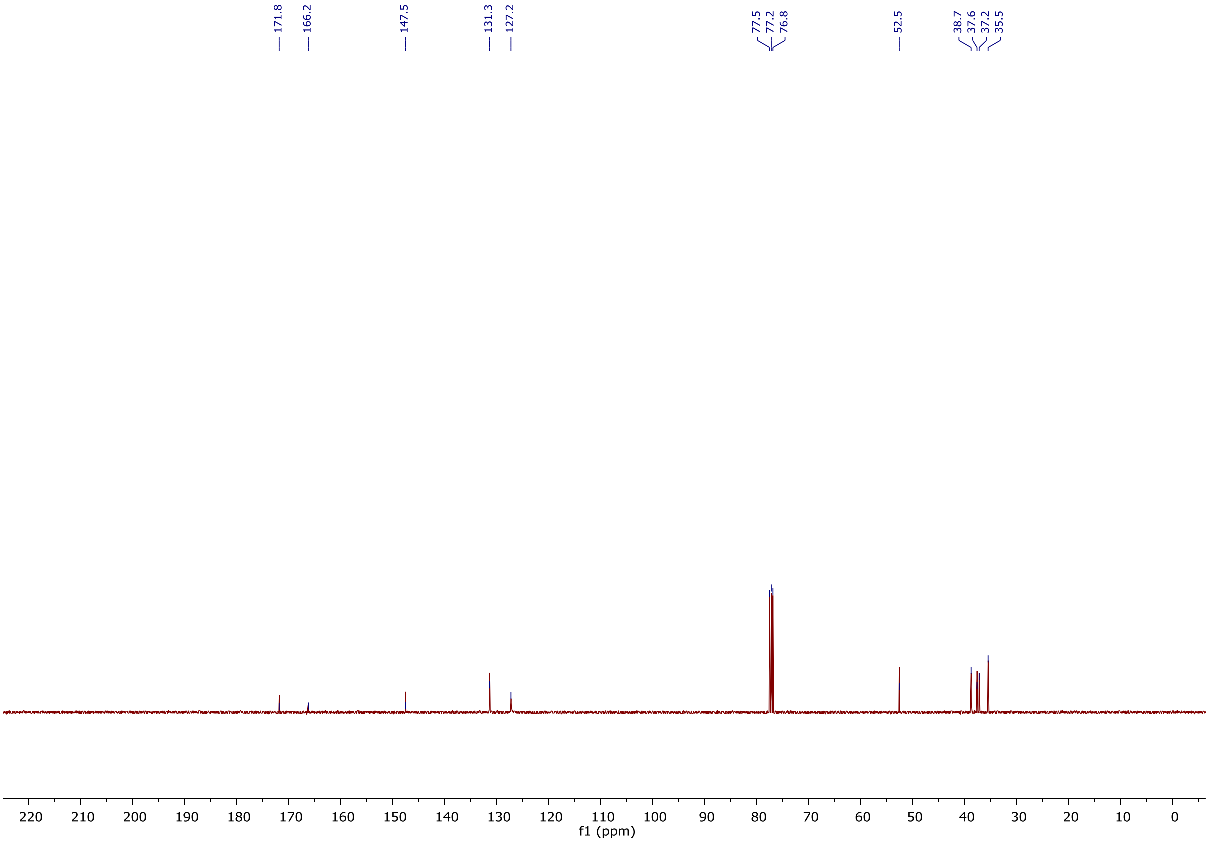

^{1}H NMR (400 MHz, $CDCl_3$) of **11**

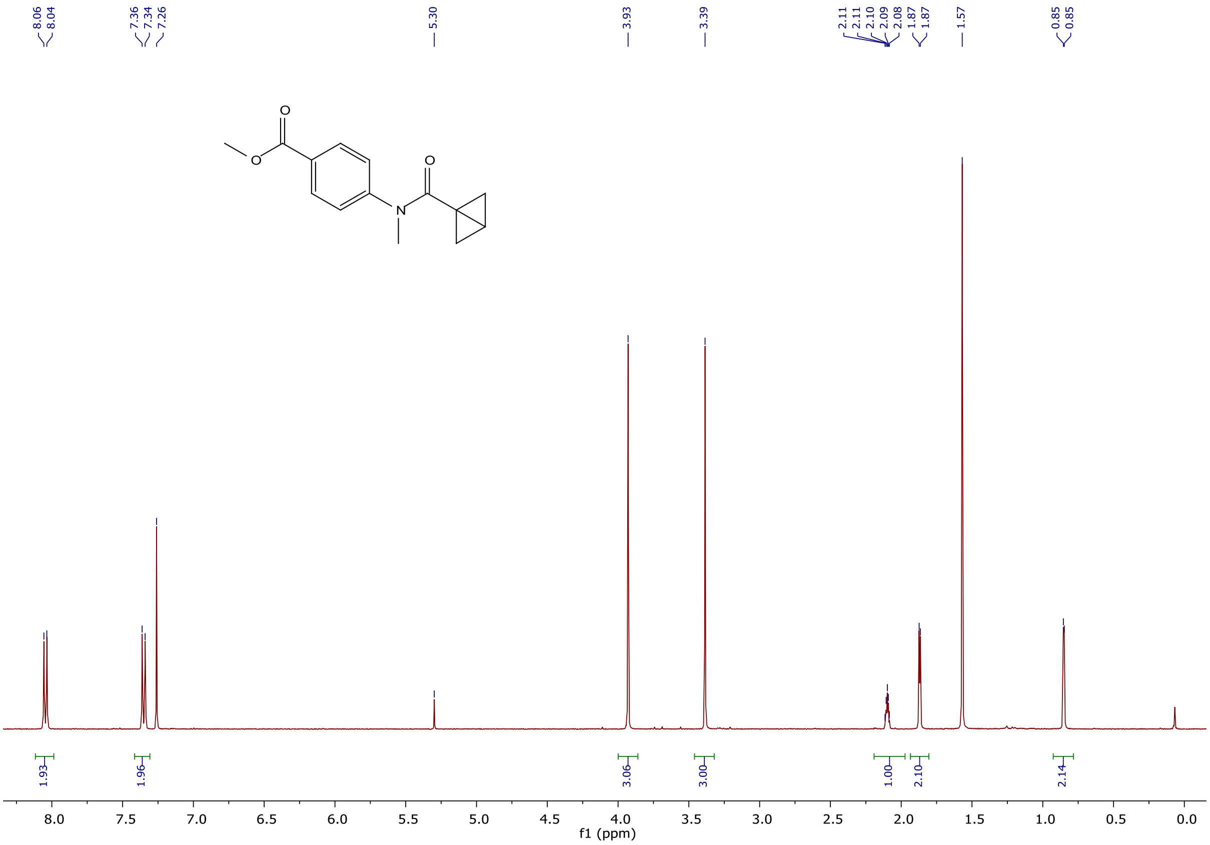

^{13}C NMR (100 MHz, $CDCl_3$) of **11**

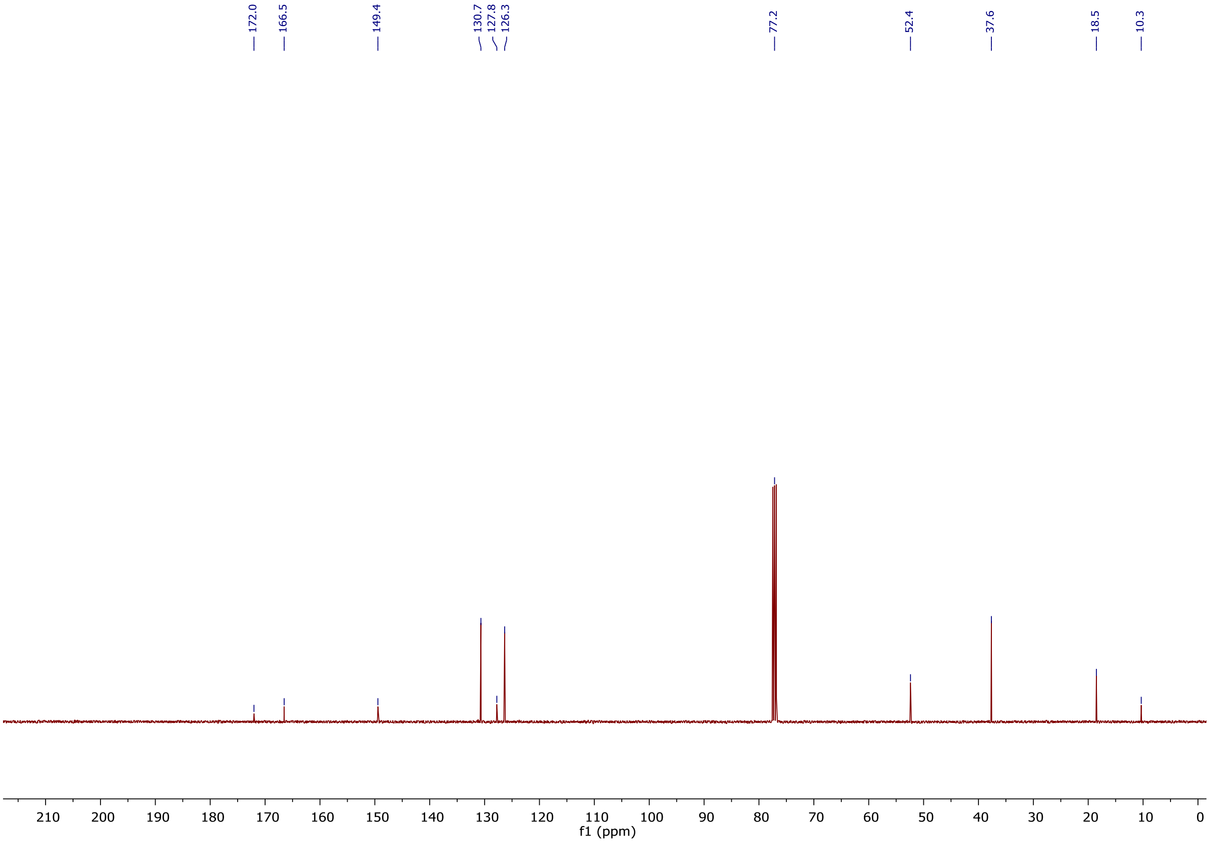

^{1}H NMR (400 MHz, CD_3OD) of **12**

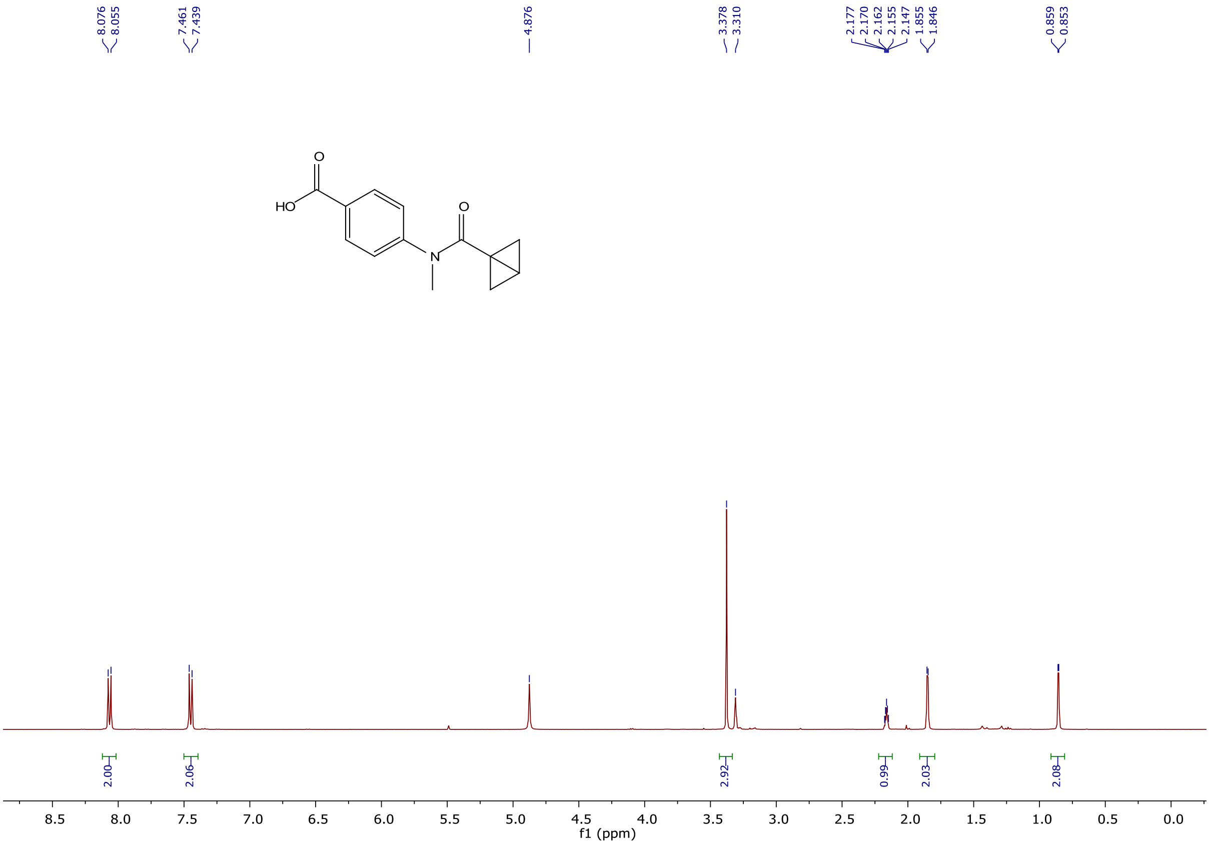

^{13}C NMR (100 MHz, CD_3OD) of **12**

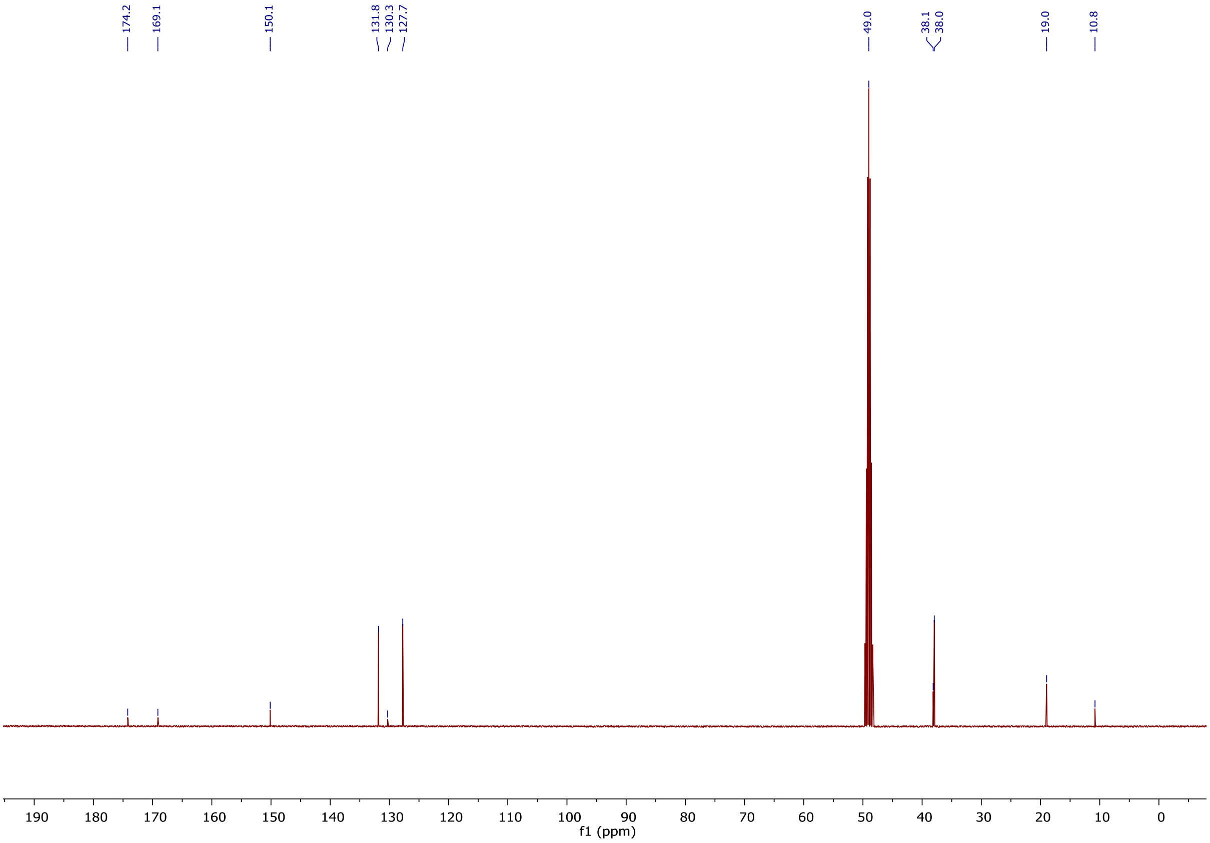

^{1}H NMR (400 MHz, $CDCl_3$) of **2**

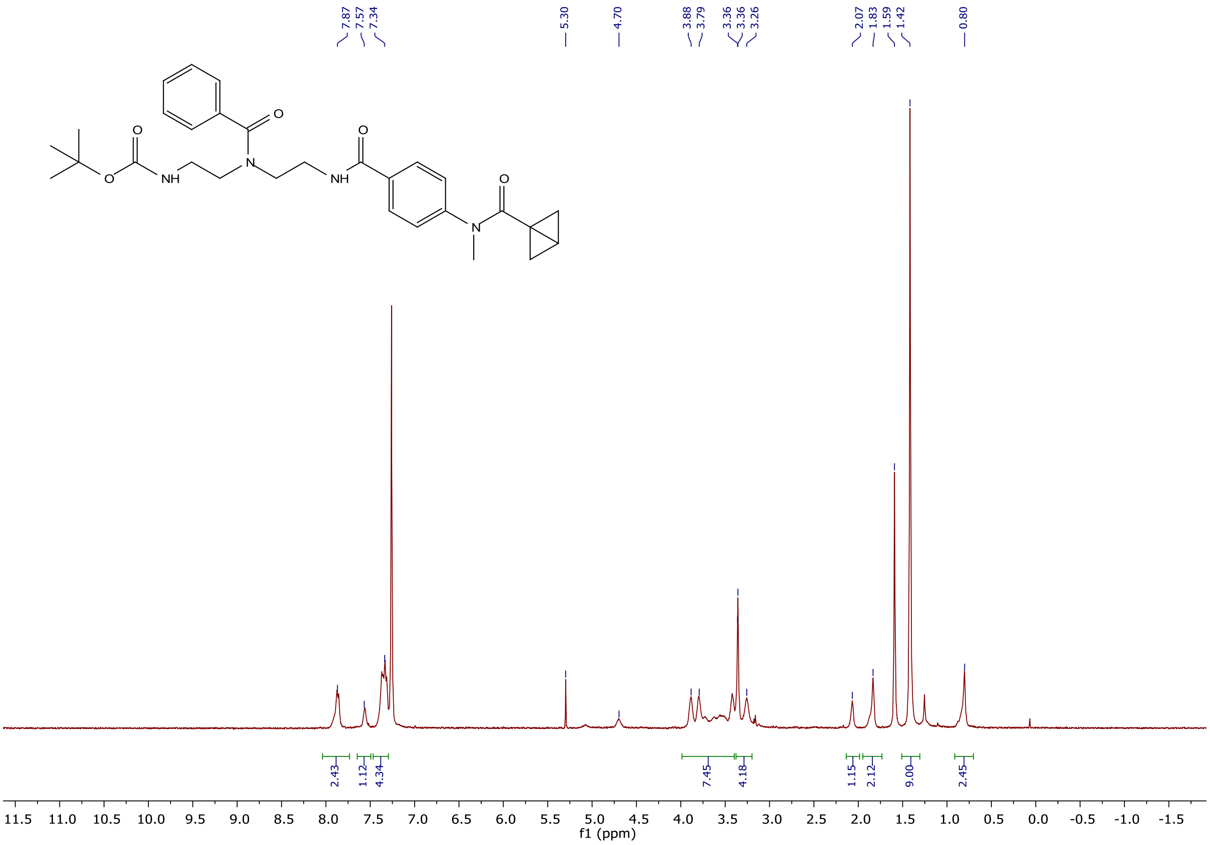

^{13}C NMR (100 MHz, CD_3OD) of **2**

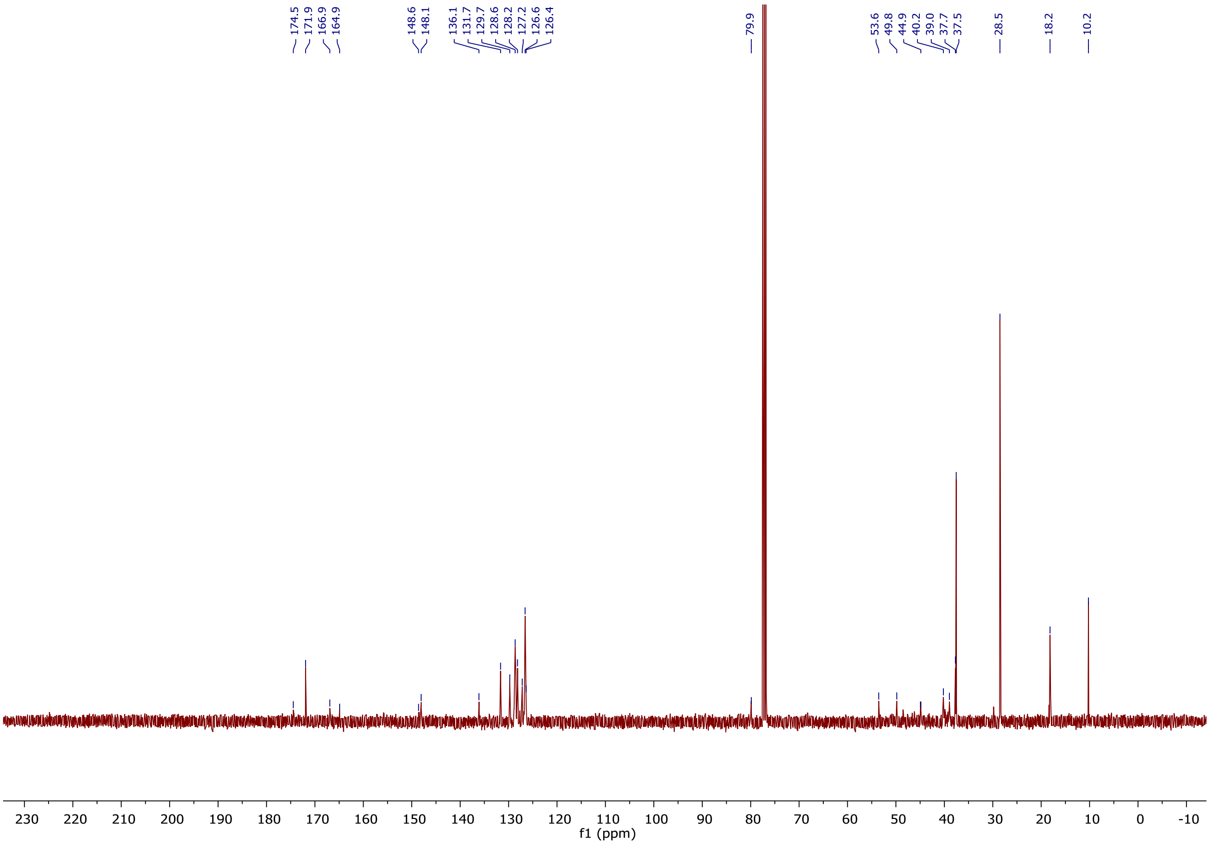

^{1}H NMR (400 MHz, $CDCl_3$) of **1a**

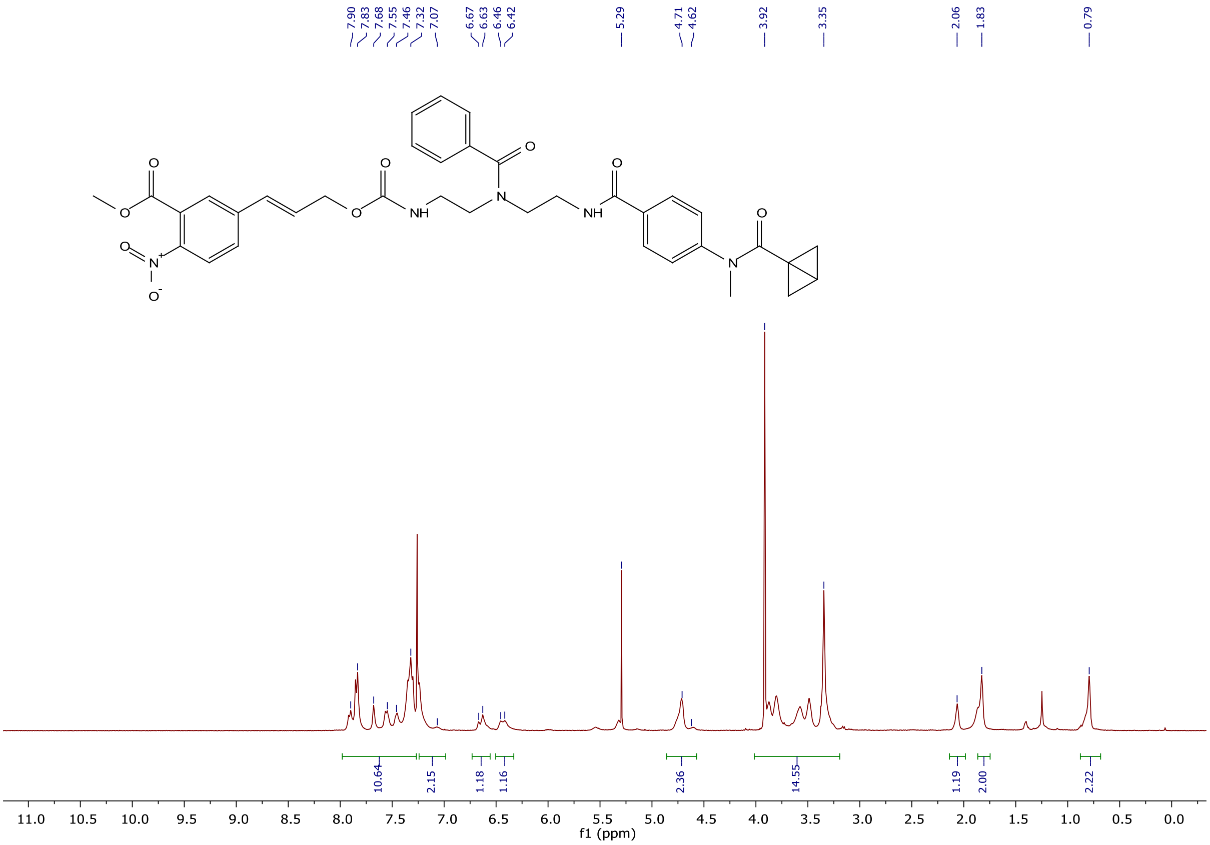

^{13}C NMR (100 MHz, $CDCl_3$) of **1a**

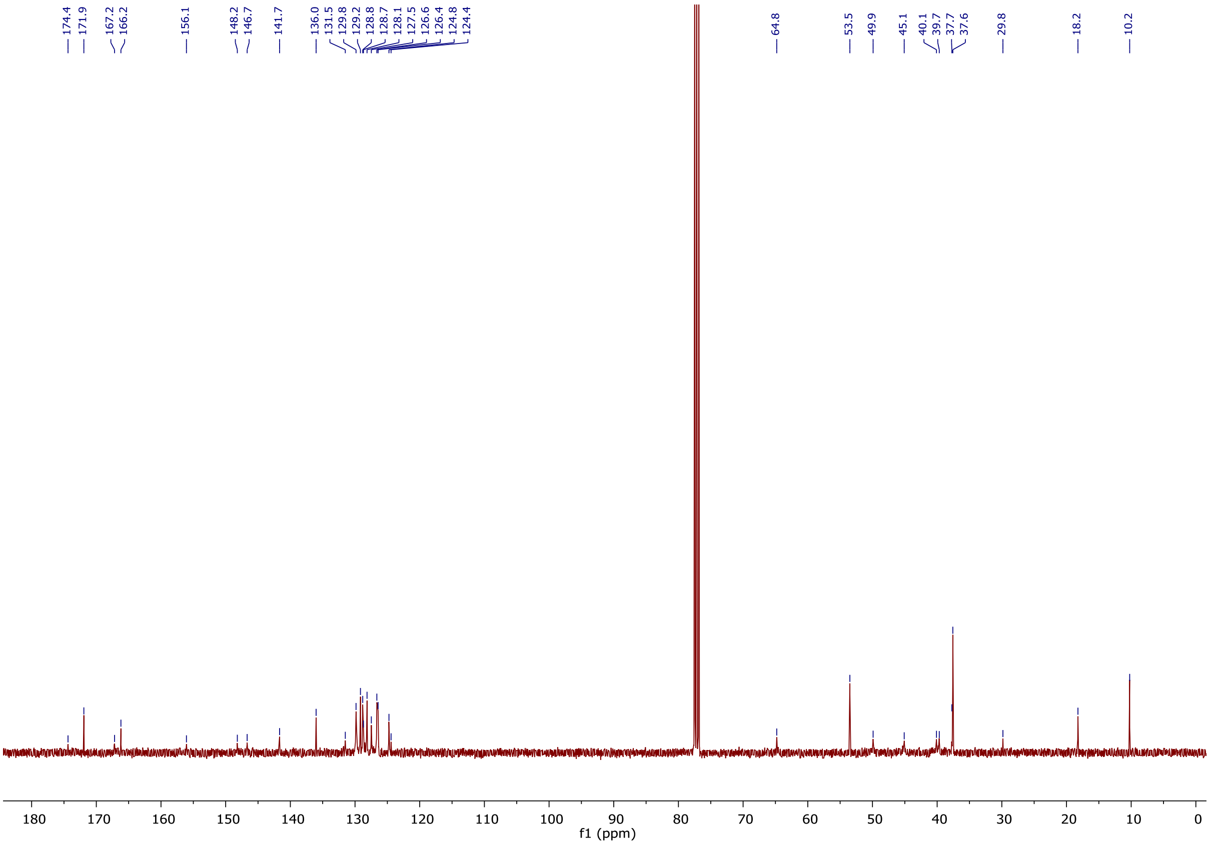

^{1}H NMR (400 MHz, CD_3OD) of **1**

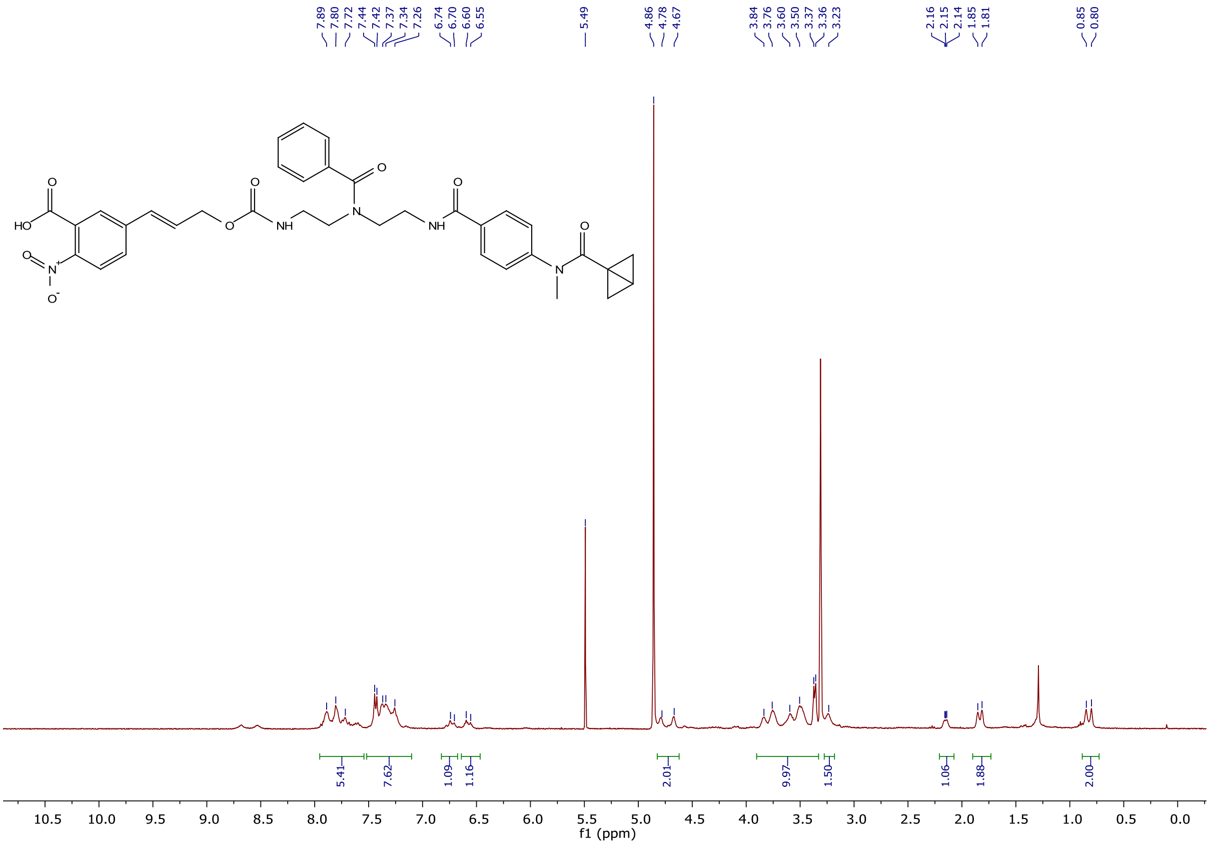

Supplement: SC-014-D3SC00224A-s001 [file SC-014-D3SC00224A-s001.pdf]
